# Brain networking analysis in migraine with and without aura

**DOI:** 10.1186/s10194-017-0803-5

**Published:** 2017-09-29

**Authors:** Marina de Tommaso, Gabriele Trotta, Eleonora Vecchio, Katia Ricci, R. Siugzdaite, Sebastiano Stramaglia

**Affiliations:** 1 Applied Neurophysiology and Pain Unit, Basic Medical, Neuroscience and Sensory System -SMBNOS- Department, Bari Aldo Moro University, Giovanni XXIII Building, Policlinico General Hospital, Via Amendola 207 A, 70124 Bari, Italy; 2 Physic Department, Bari Aldo Moro University, Bari, Italy; 3 Data Analysis Department, Faculty of Psychological and Pedagogical Sciences 1, Ghent University, Ghent, Belgium

**Keywords:** Migraine with Aura, EEG, Granger causality

## Abstract

**Background:**

To apply effective connectivity by means of nonlinear Granger Causality (GC) and brain networking analysis to basal EEG and under visual stimulation by checkerboard gratings with 0.5 and 2.0 cpd as spatial frequency in migraine with aura (MA) and without aura (MO), and to compare these findings with Blood Oxygen Level Dependent (BOLD) signal changes.

**Methods:**

Nineteen asymptomatic MA and MO patients and 11 age and sex matched controls (C) were recorded by 65 EEG channels. The same visual stimulation was employed to evaluate BOLD signal changes in a subgroup of MA and MO. The GC and brain networking were applied to EEG signals.

**Results:**

A different pattern of reduced vs increased GC respectively in MO and MA patients, emerged in resting state. During visual stimulation, both MA and MO showed increased information transfer toward the fronto-central regions, while MA patients showed a segregated cluster of connections in the posterior regions, and an increased bold signal in the visual cortex, more evident at 2 cpd spatial frequency.

**Conclusions:**

The wealth of information exchange in the parietal-occipital regions indicates a peculiar excitability of the visual cortex, a pivotal condition for the manifestation of typical aura symptoms.

**Electronic supplementary material:**

The online version of this article (10.1186/s10194-017-0803-5) contains supplementary material, which is available to authorized users.

## Background

Migraine is an incapacitating disorder of neurovascular origin consisting of episodes of headache, accompanied by autonomic and possibly neurological symptoms. The pathophysiology of migraine episodes is far from being understood, and the occurrence of aura preceding headache seems a complex mechanism related to cortical spreading depression [1, 2]. Few neurophysiological studies have compared migraine with (MA) and without aura (MO), while most of them described abnormalities of spontaneous and specially evoked brain electrical activity in the separate groups of migraine patients [3]. An abnormal response to repetitive visual stimulation, consisting of increased amplitude of steady-state visual evoked potentials (SVEPs), was observed in both MA and MO [4, 5]. Previous studies employing SVEPs at different contrast and frequency of stimulation, showed differences between MA vs MO patients, thus suggesting an involvement of the visual associative cortex in patients reporting aura symptoms [6, 7].

The study of ongoing EEG activity in basal condition and during sensory stimulation, may clarify how the migraine brain has a different reactivity, expressed by the changes of the main rhythms.under different types of stimulation. This brain behavior may predispose to the cascade of the events occurring during migraine attack, including cortical spreading depression and trigeminal-vascular system activation. In order to gain further insight into the interpretation of these phenomena, it may be necessary to investigate the dynamic interactions between brain areas, and their modulation in the presence of stimuli. An efficient measure of these interactions is the phase synchronization of the EEG signals, evaluated by investigating their phase difference. In previous studies, the phase synchronization of posterior dominant alpha rhythm (8-12.5 Hz) was lower during intermittent photic stimulation than in basal condition in healthy subjects. Conversely, in migraine without aura patients, an opposite pattern of increased alpha rhythm phase synchronization was observed [8, 9].

Methods such as correlations, spectral coherence and phase synchronization, allow to show the extent of the statistical connection of two variables, and reveal what in neuroscience is commonly referred to functional connectivity. It allows to detect common temporal features of two even distant neural populations, due to weak reciprocal interactions or shared influence of a third variable [10]. Another increasingly popular approach, effective connectivity, is based on the flow of connections and information across different brain areas. This allows to extend the insight provided by functional connectivity by telling for example which is the driver between two temporally correlated time series. Those model-based approaches can be purely data driven as Granger Causality (GC) [11–13] or biologically inspired such as Dynamic Causal Modelling [14]. In order to infer the information flow in nonlinear systems such as the brain, a flexible nonlinear generalization of Granger causality, by Kernel methods, has been recently developed [15].

These approaches represent a valuable addition to those based on correlation and synchronization analysis [8]: the results of functional and effective connectivity represent a significant added value to neuroscience, since they allow to pinpoint the temporal pattern of activation and information transfer between cortical areas [16]. In a previous study [17] we observed increased causal connections across 6 scalp derivations in beta band in MA patients compared with both MO and controls under intermittent flash stimulation, suggesting a different pattern of visual stimuli processing and cortical activation modality in patients experiencing aura symptoms. This type of causal connection may be the counterpart of a tendency to the spread of posterior cortical activation observed in MA patients observed by Functional Magnetic Resonance Imaging (FMRI) during a visual task [18]. However, the use of few derivations did not enable brain network analysis. In fact, the methods as the GC, provides simply a “map” of functional activation that can be studied in greater detail with more sophisticated tools as network analysis, the so-called “Networking” [19]. Born in computing science as an evolution of the classic graph theory, this theory deals with the description, both global and local, of the connection between the sub-components of a complex system, as one can consider the brain (in our case, the cortex only, with related areas) [19]. A number of indicators, in fact, verify if the flow of information passes unhindered through these components, if is facilitated by some areas and inhibited by others, if the network is organized in sub-structures that communicate between them or that tend to isolate themselves and so on. The networking analysis is designed for the internal dynamic of connections and the relationship between its components. The brain networking analysis may thus add knowledge to the possible differences between EEG behaviors of patients experiencing or not reporting aura symptoms, aiming to clarify the possible reason of this phenotypical characteristic.

## Objectives

This study aimed:

1) To conduct transfer entropy analysis across brain regions **under pattern reversal visual stimulation** in both types of migraine and controls, by means of multichannel EEG recording, thereby obtaining in this way information from effective connectivity patterns.

2) To evaluate subtle differences in Brain Networking between the two types of migraine.

2) To localize the brain areas involved in a possible different response to the pattern reversal visual stimuli in two subgroups of migraine with and without aura patients, observing the changes of BOLD signals by Functional Magnetic Resonance Imaging (FMRI).

## Methods

### Subjects

EEG was recorded from 19 patients experiencing aura (MA) (5 males, age range 18-44, mean 32.2 ± 7.5, education level 13.2± 1.2 years), for whom a diagnosis of typical aura with migraine headache [19] (IHCIII cod. 1.2.1) and non-migraine headache (IHCIII cod. 1.2.2) was performed [20]. Nineteen migraine without aura patients (2 males, age range 18-46, mean 33.2 ± 5.6. education level 13.8 ± 2.1 years) (IHCIII code 1.1.) [20] were also included in the study. Frequency of headache was 3.5 ± 0.8 and 2.9± 1.1.days/headache/month respectively for MO and MA patients. Migraine history lasted for 10.1 ± 4.5 years in MO and 9.1± 3-8 years in MA patients. All patients were in the interictal state, the time from the end of the last attack being at least 72 h, while an interval of at least 48 h from the next attack, ascertained by a telephonic interview. This was done in order to exclude patients in the phases preceding migraine. Females were recorded about 15 days after menses. No patient was under preventive treatment nor used symptomatic drugs in the 72 h preceding the recording session. Eleven healthy subjects (1 male, age range 18-43 mean 31.9 ± 6.5, education level 14.2 ± 1.8), matched for sex and age with the patients groups, not reporting migraine in first-degree relatives, were also included as controls. In control group, females were about 15 days after menses. The migraine groups were similar as regard to age (ANOVA: F 0.08, p 0.78), sex (chi square 0.019 p 0.89), and frequency of headache (F 2.84 p 0.11), migraine history (F 0.35 p 0.56). Patients and controls were similar for age (ANOVA F 0.11 p 0.89), sex (chi square 0.12 p 0.88) and years of school (ANOVA F 0..12 p 0.88). None of the subjects involved in the study were affected by general medical, neurological and psychiatric diseases, including anxiety and mood disorders. The protocol was approved by the Ethical Committee of Bari Policlinico General Hospital, and each subject signed an informed consent. All patients and controls gave their consent to the data publication.

### Recording and stimulation procedure

Black and white checkerboard patterns generated on a 17- in. television subtended 21 × 17° at a viewing distance of 90 cm was presented during EEG and MRI recordings. Two spatial frequencies, 0.5 and 2.0 cycle per degree (cpd), were presented. The mean luminance was 14 cd/m2. For both spatial frequencies the stimulus pattern was alternated at 5 Hz (10 reversal/s). In fact our stimulation system did not support higher stimulation frequencies. EEG data were recorded by 62 scalp electrodes, according to enlarged 10–20 system (Fp1, Fpz, Fp2, F7, F3, Fz, F4, F8,T3, C3, Cz, C4, T4, T5, P3, Pz, P4, T6, O1, Oz, O2, FC2,FC1, CP1,CP2, PO3, PO4, FC6, FC5, CP5, CP6, AF7, AF3,AFZ, AF4, AF8, F5, F1, F2, F6, FT7, FC3, FCZ, FC4, FT8,C5, C1, C2, C6, TP7, CP3, CPZ, CP4, TP8, P5, P1, P2, PO7,POZ, PO8, with impedance below 5000 Ω), referring to the nasion with the ground at Fpz. Another electrode was placed above the right eye to record the EOG. Signals were amplified, filtered (0.5 to 80 Hz, at a sampling rate of 256 Hz), and stored on a bio potential analyzer (Micromed System Plus; Micromed, Mogliano Veneto, Italy; http://www.micromed.eu.) The sampling rate was 256 Hz.

### Images acquisition

MRI scans were acquired at 1.5 Tesla (G.E. SIGNA). BOLD fMRI data were collected using gradient-echo echo planar imaging with 160 volumes at repetition time (TR)¼ 3 s. Anatomical images were acquired using a T1-weighted magnetization prepared rapid gradient echo (3D MPRAGE) at 1 mm resolution with 160 slices. A subgroup of 8 MA and MO patients (all females, MA 32.2± 5.6 years old, MO 33.3 ± 5.7 years old), submitted in the same day to the EEG recording session, were also stimulated by the same checkerboard pattern stimulation as described above, while acquiring MRI scans.

### EEG analysis

The EEG records were preliminary inspected by the first author, who was not aware of the subjects’ identity and diagnosis. An automatic artefact rejection, considering 150uV as critical value of amplitude and the similarity of EOG channel, was applied to all EEG recordings. Records or portion of records that will contain drowsiness, sleep or persistent ocular artifacts were deleted.

### Granger causality

In this study we evaluated effective connectivity by means of Granger Causality (GC) and Transfer Entropy (TE), using the nonlinear generalization of GC, by Kernel methods, presented in our previous study [14, 15, 21], which allows to infer the directional information flow in nonlinear and multivariate systems [22, 23].

In addition, the connection matrix belonging to GC and TE analysis was considered in order to build the Information Network by means of the Mat Lab(C) Brain Network Toolbox (BCT) by Rubinov & Sporns [19]. This feature allows us to reconstruct the spatial distribution of nodes (the electrodes) and links (the amount of information flowing between two nodes) of an information network and to evaluate some of its characteristic features.

The next step consisted in the evaluation of four classes of features: Integration (ability of the network to rapidly combine specialized information from distributed brain regions), Segregation (ability for specialized processing to occur within densely interconnected group brain regions),

Centrality (a measure of the ability of a given brain region to facilitate functional integration) and Resilience (ability of the network to regenerate interrupted links by-passing through different regions or connections). Finally, the results were compared with the null model provided by the BCT itself.

The statistical analysis was conducted, for each channel and for each band, after a Kolmogorov Smirnoff test application to confirm the parametric distribution of data, using the one-tail (alternating left and right) t -Student test with a Bonferroni correction for multiple comparisons equal to

$$ \mathrm{b}=\mathrm{n}\ \mathrm{couples}\times \left(\mathrm{n}\ \mathrm{stims}-1\right)=3\times 2=6 $$

In this case the universally accepted value of 0.05% c.l. is lowered to 0.05% / b (in our case, 0.008%, or 0.004% for each tail).

Statistical probability maps provided the significant differences between groups for the number of information transfer (entry in; exit out) involving all the electrodes. The color convention we used was the following: warm colors if the distinction was in favor of the first element of the comparing couple (e. g, in MA/MO case, red is referred to MA), cool colors otherwise (in the example, blue is the distinction in which MO levels are larger). The analysis of transfer entropy focused on the 3 groups (MA, MO and C), while in order to look for subtle differences between the two types of migraine, the Brain Networking analysis focused on the differences between the migraine groups. Further details of the analysis are reported in the Additional file 1 Section.

### **Image analysis** (BOLD fMRI data)

The echo planar scout image from each subject were co-registered to its anatomical image and used as the target for six-parameter, least squares rigid body realignment. The BOLD time series data were then interpolated in time to correct for the interleaved slice acquisition sequence. The volumetric beta maps of percentage signal response to visual stimulation were projected from each subject to the cortical surface atlas. Each map underwent 10 mm, two-dimensional Gaussian smoothing restricted to the cortical sheet. A map-wise, random-effects analysis across subjects was used to model the effects of group (MA vs MO) on cortical response. The two groups of subjects were compared using t test for all the visual stimulation pattern.

## Results

We verified that the data distribution for each channel for the considered bands (delta 0.5-3-5 Hz, theta 4-7 Hz, alpha (8-12 Hz, beta 13-30 Hz) were parametric, according to the Kolmogorov-Smirnov test.

### Analysis-effective connectivity: Transfer entropy

**Fig. 1:**
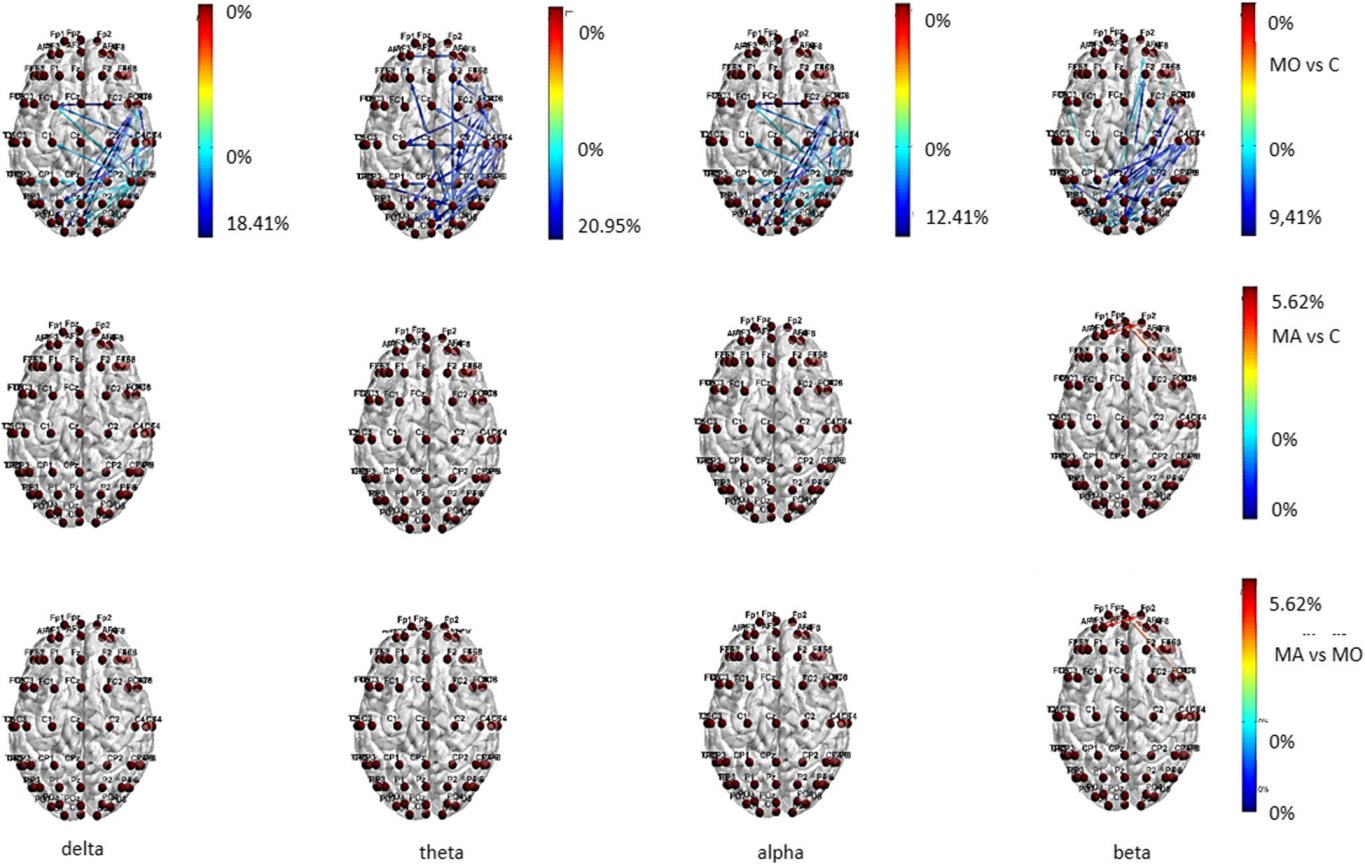
Statistical differences in information transfer and their relative directions among nodes in resting state EEG bands are depicted in the Migraine with aura (MA), Migraine without aura (MO) and Controls (C) groups . Hot colors refer to a statistical prevalence of information transfer in the first element of the comparison, and cold colors for the vice-versa. The color intensity is graduated on the relative percent difference

BASAL EEG There were some differences in behavior between the MA and MO patients, considering that MO patients showed reduced functional connections in the temporal-parietal posterior regions in all bands, and also in frontal regions in theta band, while this pattern was absent in MA patients (Fig. 1) In beta band MA patients showed increased information transfer compared to controls and MO patients (Fig. 1) in both input and output direction. (Fig. 1).

### VISUAL STIMULATION- 0.5 cycles/degree spatial frequency

**Fig. 2:**
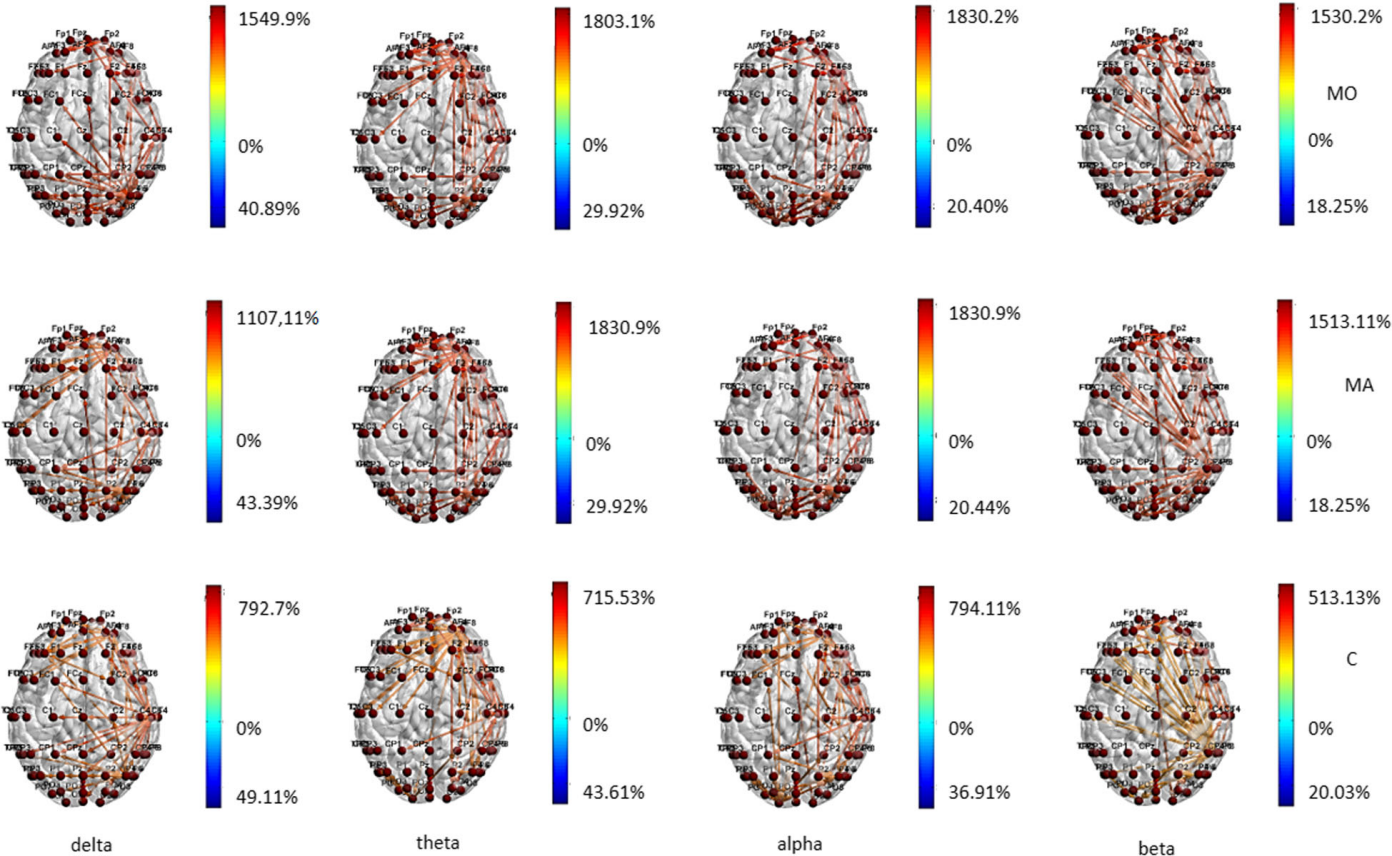
Statistical differences in information transfer and their relative directions among nodes are depicted for the comparison of EEG bands under 0.5 cpd spatial frequency visual stimulation vs resting state condition in the Migraine with aura (MA), Migraine without aura (MO) and Controls (C) groups . Hot colors refer to a statistical prevalence of information transfer in the 0.5 cpd spatial frequency visual stimulation condition, cold colors for a prevalence in resting state. The color intensity is graduated on the relative percent difference. (further data on statistical analysis are available in the Additional file 1: section, chapter 5.6.1)

During visual stimulation with 0.5 cycles/degree size checkerboard, significant changes in transfer entropy occurred in comparison to basal EEG in all the considered bands in the three groups (Fig. 2). The direction of information transfer was from the parietal-occipital regions toward the frontal ones (Fig. 2).

**Fig. 3:**
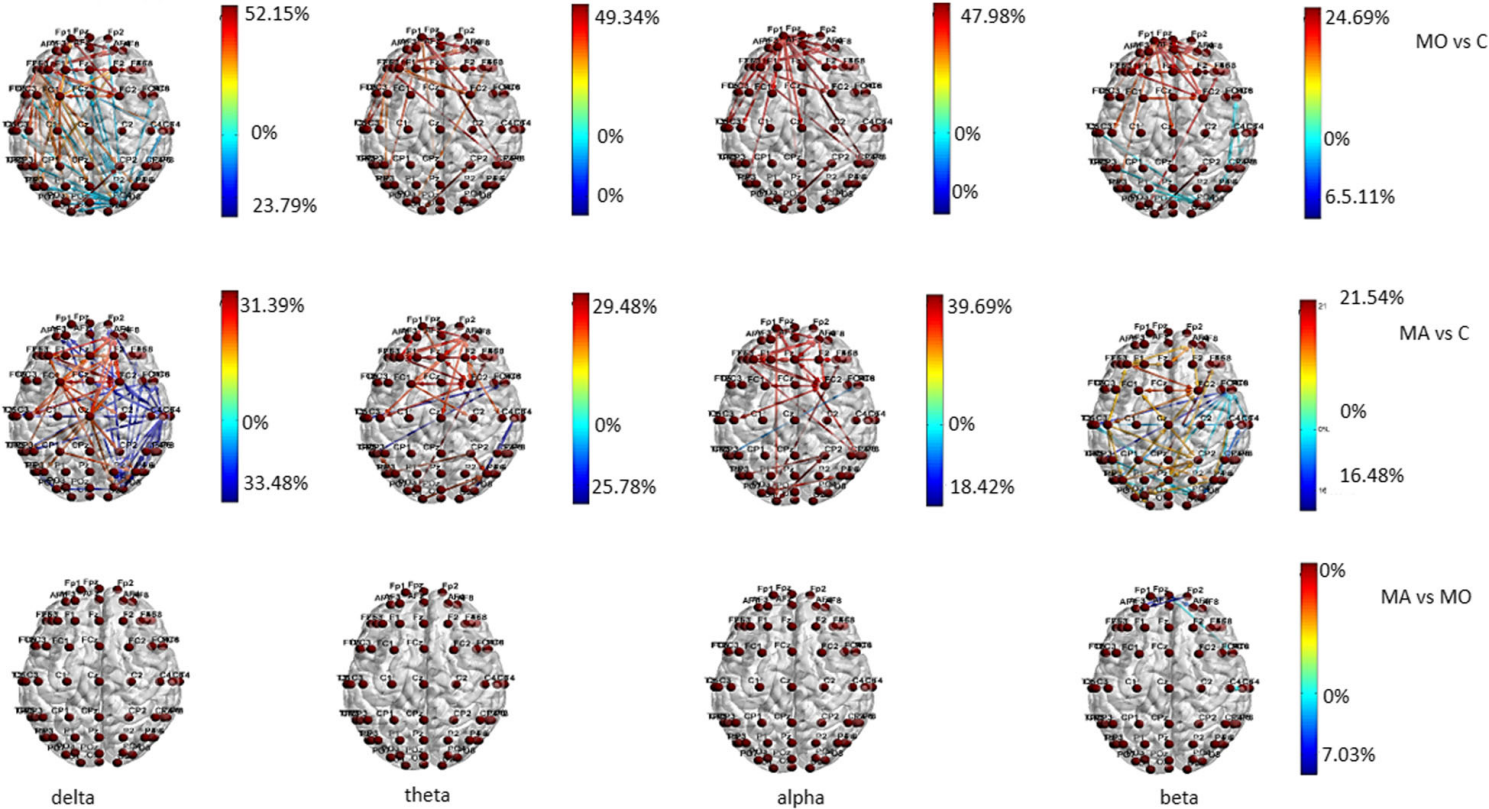
Statistical differences in information transfer and their relative directions among nodes during 0.5 cpd spetial frequency are depicted in the Migraine with aura (MA), Migraine without aura (MO) and Controls (C) groups . Hot colors refer to a statistical prevalence of information transfer in the first element of the comparison, and cold colors for the vice-versa. The color intensity is graduated on the relative percent difference. (further data on statistical analysis are available in the Additional file 1: section, chapter 5.6.1)

Both migraine groups showed increased connections in frontal regions in all the considered bands, when compared to controls, who activated preferentially the temporal-parietal regions, especially in delta band (Fig. 3). In MO patients the information flow across the frontal regions was more evident than in MA group, at least in beta band (Fig. 3). The latter showed a richness of connection in the central regions.

**Fig. 4:**
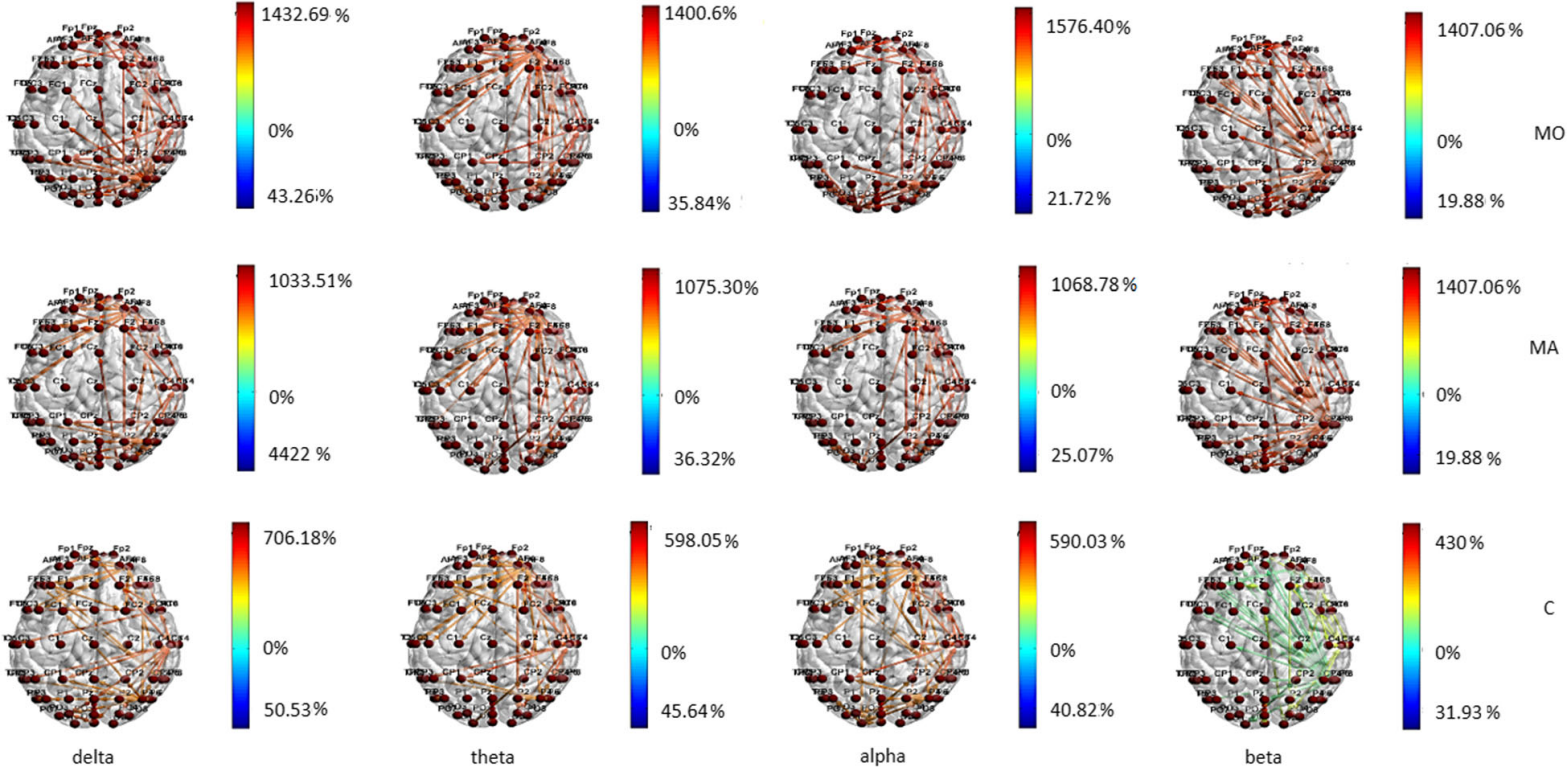
Statistical differences in information transfer and their relative directions among nodes are depicted for the comparison of EEG bands under 2 cpd spatial frequency visual stimulation vs resting state condition in the Migraine with aura (MA), Migraine without aura (MO) and Controls (C) groups . Hot colors refer to a statistical prevalence of information transfer in the 0.5 cpd spatial frequency visual stimulation condition, cold colors for a prevalence in resting state. The color intensity is graduated on the relative percent difference. (further data on statistical analysis are available in the Additional file 1: section, chapter 5.6.1)

Two cycles/degree spatial frequency. A similar trend of information flow between temporal-parietal and frontal regions was also present for EEG related to 2 cycles/degree checkerboard in migraine groups, with MO group activating preferentially the frontal-polar region and MA patients the frontal-central ones. This phenomenon was less evident in controls, especially in beta band (Fig. 4).

**Fig. 5:**
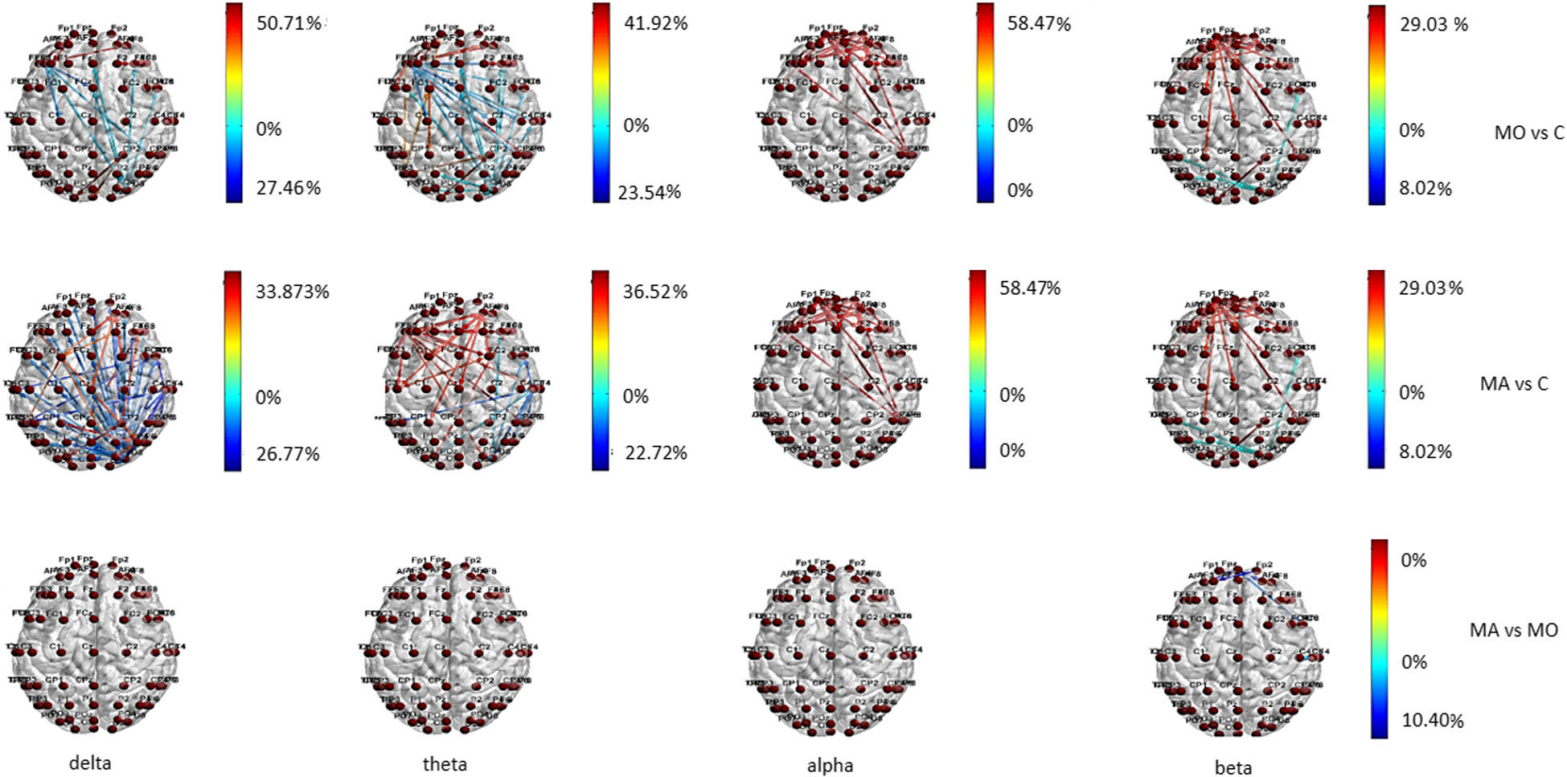
Statistical differences in information transfer and their relative directions among nodes during 2 cpd spetial frequency are depicted in the Migraine with aura (MA), Migraine without aura (MO) and Controls (C) groups . Hot colors refer to a statistical prevalence of information transfer in the first element of the comparison, and cold colors for the vice-versa. The color intensity is graduated on the relative percent difference. (further data on statistical analysis are available in the Additional file 1: section, chapter 5.6.1)

In MA group, the transfer entropy in out direction from the frontal toward the central regions was more expressed than in controls (Fig. 5) in all the considered bands. In beta band the MA group showed a reduced information flow within the frontal-polar derivations in comparison to MO patients (Fig. 5).

### Brain networking analysis

**Fig. 6:**
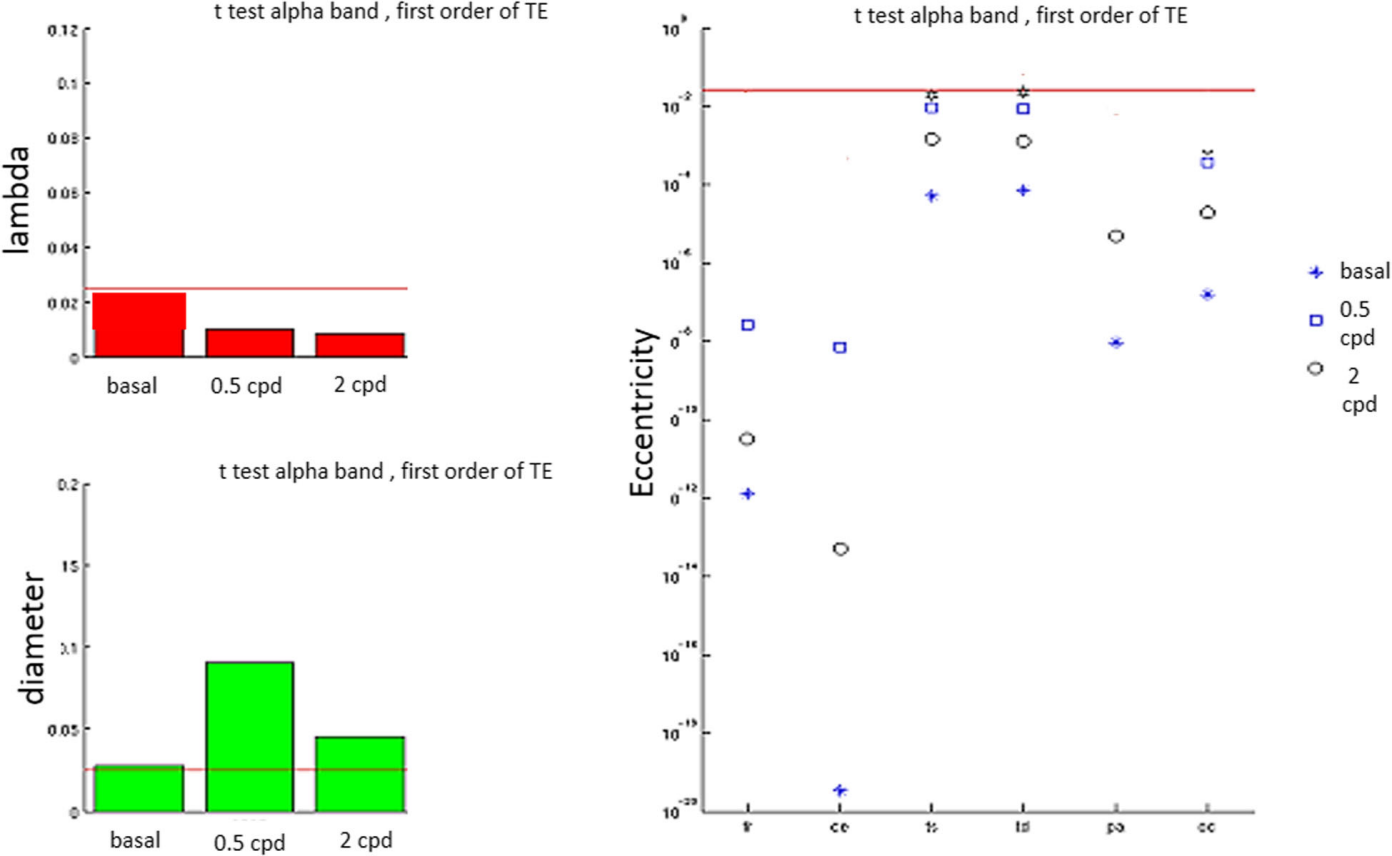
ANOVA tests on Characteristic Path Length features of Migraine with Aura versus Migraine Without Aura. The first column shows the Student t-test on the “lambda” and “diameter” factors (which stem for the medium path length and its distribution) in basal condition and during 0.5 cpd and 2 cpd spatial frequency visual stimulation. The lambda is clearly smaller for MA, as one can argue observing the “eccentricity” (distribution of the minima, right column) at all the stimulations and for all cortical bands (alpha band is here represented). The red horizontal line stems for the t-test confidence level (Bonferroni corrected) under which the probability of having distinct population is relevant

Integration Measures - Characteristic Path Length The analysis of the Characteristic Path Length (CPL) showed that MA patients were more segregated (i.e. less incline to share information) and less integrated and efficient than the MO ones, having a smaller path length (i.e., a greater distance) between nodes and a greater clustering coefficient, at least during visual stimulation (Fig. 6).

**Fig. 7:**
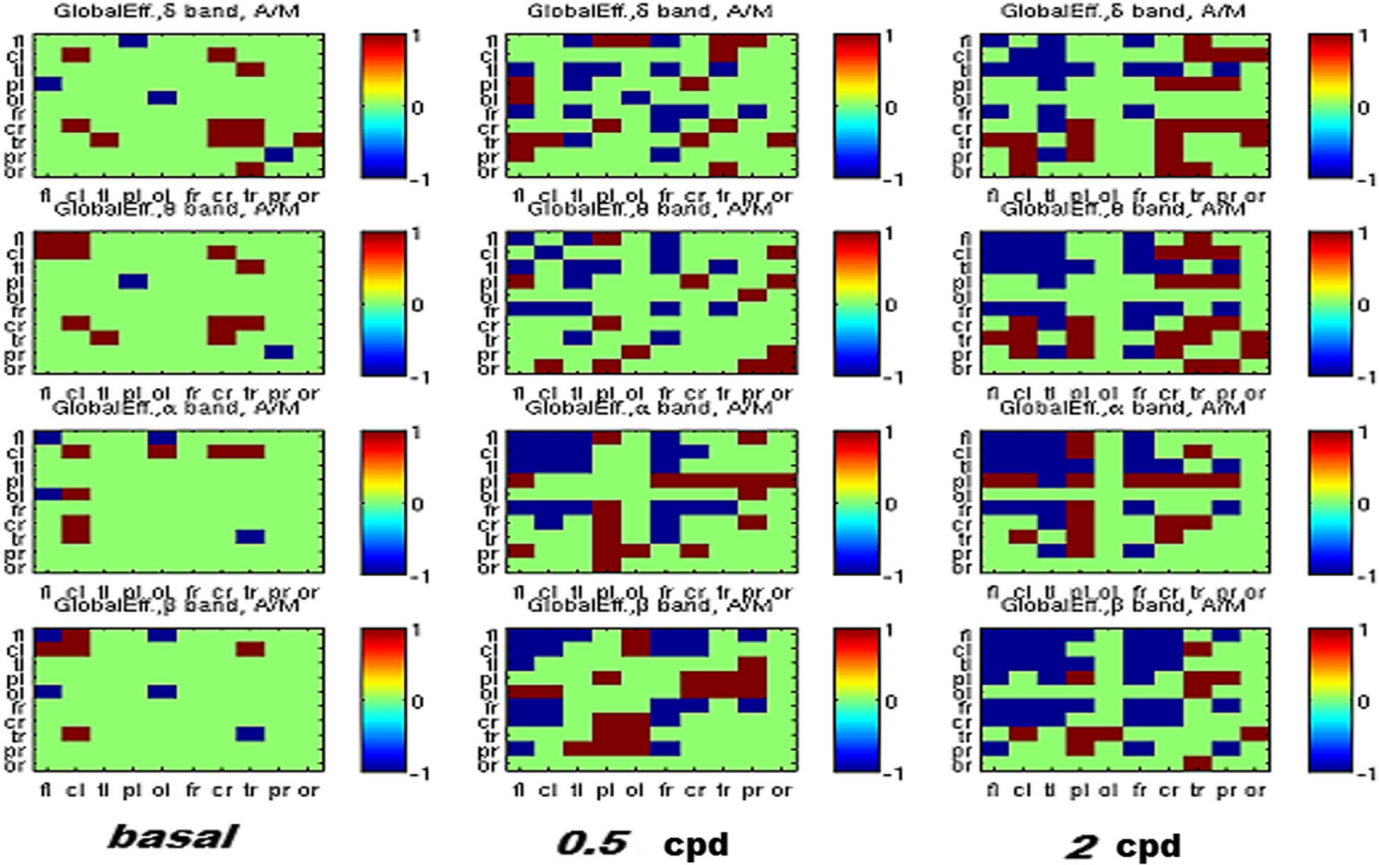
Comparison of global efficiency in Migraine with aura (MA) and Migraine Without Aura populations: the matrix elements in red (+1) account for larger values in MWA, while those in blue (−1) account for larger values od MWoA. Green cells mean no distinction. (further data on analysis are available in the Additional file 1: section, chapter 5.6.2) The EEG electrodes were merged into 4 main scalp zones: fl, frontal, cl, central, tl, temporal, pl, parietal, ol, occipital

Global efficiency The global efficiency in different bands during visual stimulation with both spatial frequency, showed that MO patients increased the intra-hemispheric global efficiency with respect to the MA, especially in frontal-central areas. At the same time, the MA showed a larger efficiency in sorting information from the left to the right parietal-occipital areas (higher inter-hemispherical efficiency) (Fig. 7).

**Fig. 8:**
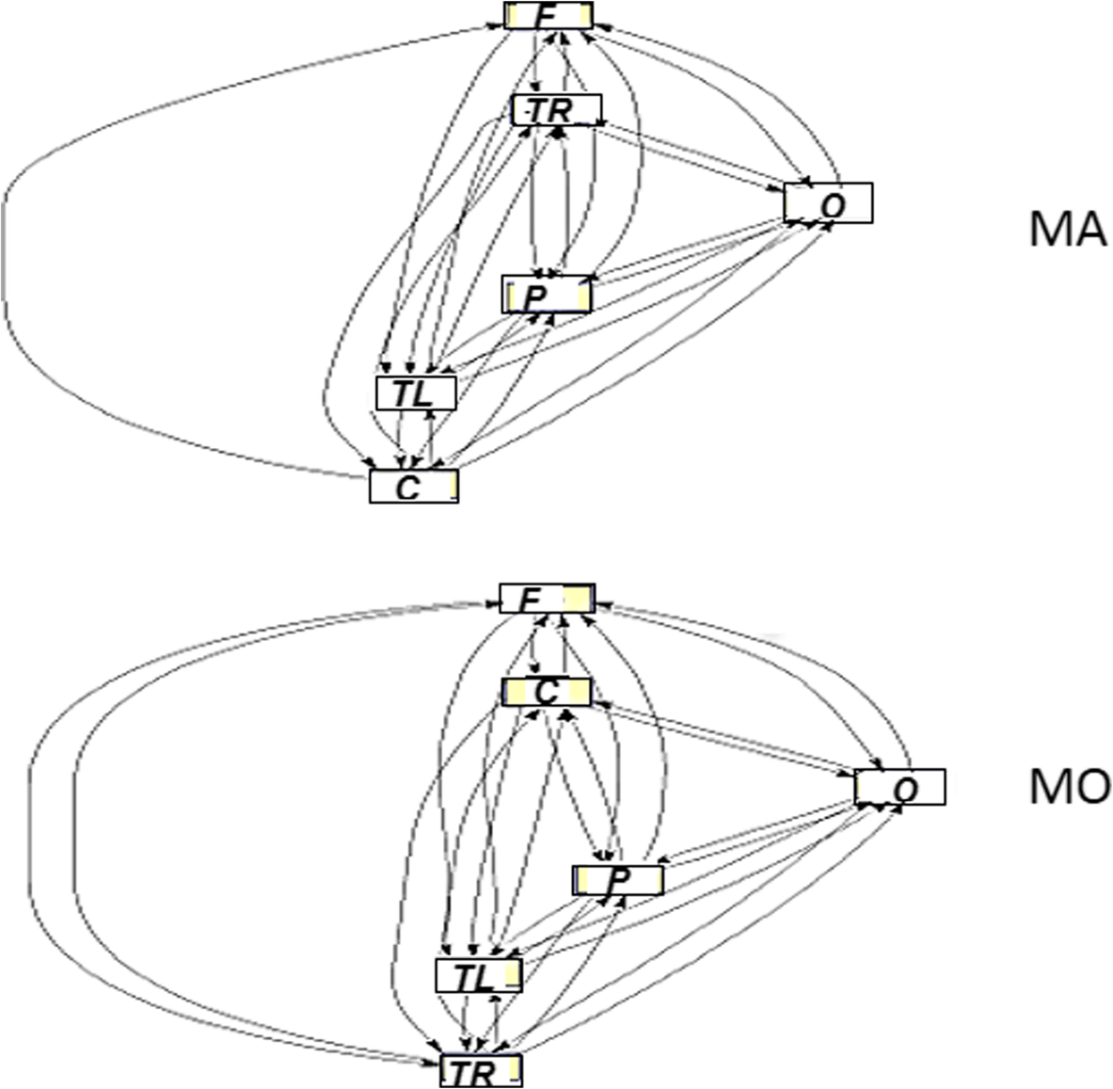
Network diagrams for Migraine with aura (MA) and without aura (MO) for alpha band at 2 cpd stimulation. The by-pass role of the occipital area (O) in the MA in the anterior / posterior connection emerges. (further data on analysis are available in the Supplementary section, chapter 5.6.2) F: Frontal; C: Central; P: Parietal; RT: Right Temporal; LT: Left Temporal

Centrality measures. The analysis of the betweeness centrality started from the Vertex variant (VBC), and clearly showed how, in the comparison between the two phenotypes of migraine, the stimulation with both spatial frequencies caused the parietal-occipital areas to be more central in MA compared to MO of about 35% in the higher frequency bands (alpha and beta). Even the analysis of the Edge variant (EBC) clearly showed an increased centrality of connections between parietal-occipital regions to almost all other areas in the MA group, the posterior areas having a “bypass” function for the information. This was indicative of the fact that the parietal-occipital areas of MA behave essentially as local hubs of the network, or as areas facilitating the functional segregation of the posterior areas of the cortex (Fig. 8).

### Bold signal

**Fig. 9:**
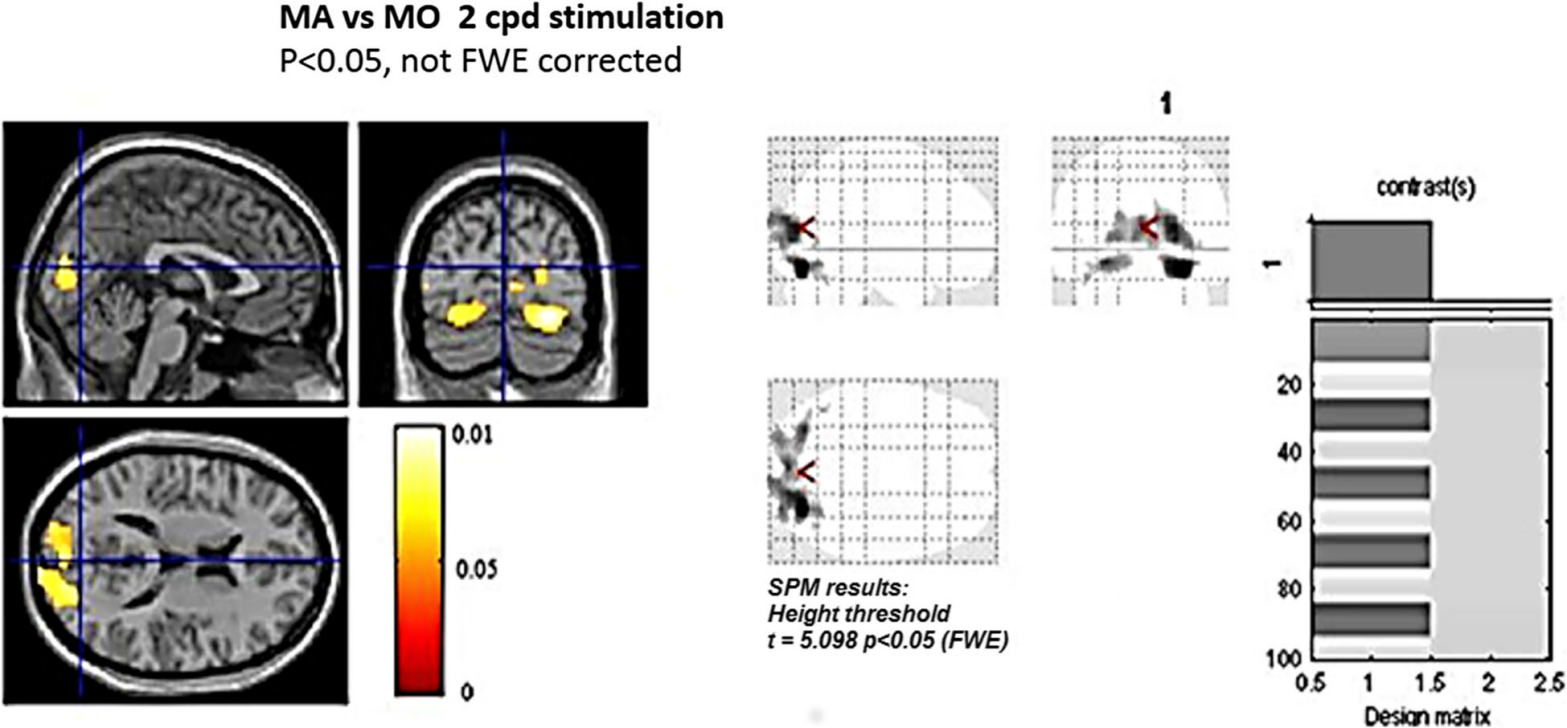
Statistical probability maps reporting the comparison of bold signal changes in migraine with aura vs migraine without aura sub groups during 2 cps spatial frequency visual stimulation

The map-wise, random-effects analysis showed that the 2 Hz mm spatial frequency stimulation caused a significant increment of bold signal in bilateral primary visual and extra striate cortex in MA compared to MO patients (Fig. 9). This result approached the statistical significance for the 0.5 cpd spatial frequency.

## Discussion

The present innovative analysis conducted on the background EEG activity in migraine patients with and without aura, confirmed evident differences of EEG rhythms in respect to controls, even in basal conditions, and more clearly during visual stimulation. Subtle differences emerged also between the two types of migraine in terms of transfer entropy and brain networking, which may be discussed in light of the phenotypical characteristic of the presence or absence of aura symptoms perception, as well as in regard to the anatomical differences of cortical activation observed by FMRI.

Transfer entropy. Reduced causal connections characterized migraine without aura groups as compared to controls, even in basal EEG, with special regard to the temporal-parietal regions. This pattern was not evident in MA patients, who conversely showed that the frontal regions were more connected as compared to patients not experiencing aura symptoms. The reduced transfer entropy with low density of information flow across the temporal-parietal regions observed in MO patients, may be the counterpart of the increased synchronization of EEG rhythms reported in previous studies [9]. The different pattern of reduced synchronization and increased causality and transfer entropy in beta band, present in MA, was reported in another study [17], and could confirm subtle differences in connectivity pattern among the two migraine phenotypes, which may emerge even in resting state, if a multichannel recording is employed. Few studies have compared MA and MO patients taking into consideration the connectivity analysis of basal EEG rhythms. Altered coherence of low frequency EEG was observed in a MEG study of migraine without and with aura, the latter type of migraine presenting with a pattern of reduced inhibitory connections in theta band [24]. The different signals, MEG vs EEG, and the different methods employed to study connectivity, coherence vs granger causality, can justify the different results obtained in the two studies, though both of them reported a different pattern of causal connections in migraine vs controls, and reduced inhibitory connections in migraine with aura. The most of connectivity studies in migraine were performed by functional connectivity analysis in resting state FMRI, though the time resolution of EEG signal better enables the reconstruction of dynamic causal connections across different scalp regions. However, the general impression emerging from migraine connectivity studies is that very different and hardly comparable recording and analysis methods are used. In any case, the presence of connectivity modifications in the asymptomatic phase of migraine is a very robust data [25–29], although the differences between the two forms of migraine are less clear. Coppola et al. [30] observed a reduced function of oscillating networks across the brainstem, the thalamus and the cortex in the default mode network and the visual spatial system in migraine without aura compared with controls, a finding that may be comparable with our observation of reduced causal connections in almost all bands in the MO group. Tedeschi et al. [31] reported a pattern of increased functional connection within the visual network in migraine with aura in comparison with migraine without aura and controls. Our connectivity study on multichannel EEG can confirm a tendency toward reduced information flow in migraine without aura patients even in resting state, with subtle differences from migraine with aura, characterized by an increased amount of information transfer in high frequency –beta-oscillations. Considering that the only difference between the two forms of migraine is the perception of visual or sensory symptoms preceding headache, it is probable, as previously hypothesized [17] that in the form of migraine without aura, inhibitory mechanisms within neuronal networks can block the progression of the bioelectric phenomenon preceding the attack.

During visual stimulation with checkerboard pattern, both migraine groups exhibited a richness of cortical connections with a clear different information transfer in the frontal regions, both in in and out direction, in respect to controls. The organization of the inter-connections among the scalp electrodes followed a model completely different from that exhibited by non-migraine patients, and the distribution of the connections was also different between the 2 forms of migraine, also if the comparison approached but not satisfied the statistical significance. In respect to controls, migraine with aura patients activated preferentially the frontal-central regions in all the bands, while the connections were more concentrated in the frontopolar regions in migraine without aura patients, with a pattern particularly evident with the small size-checkerboard, which was also reported by Shibata et al. to differentiate the 2 form of migraine, at least for steady state visual evoked potentials amplitude [6, 7] However, despite the architecture of the connections between the 2 forms of migraine was different, the only statistical significance that emerged was in favor of a prevalent activation of frontal-polar regions in beta rhythm in migraine without aura. Considering the amount of statistical comparisons, we can also suppose that an enlargement of cases series might enable to show a statistical difference between the 2 models of connections characterizing the 2 forms of migraine. This type of visual stimulation did not cause an opposite pattern of connections inhibition and activation, respectively observed in MO and MA patients in our previous experiment with an intermittent luminous stimulation [17]. In the present multichannel recording, this opposite connections behavior emerged in the resting state EEG, while during the stimulation with the checkerboard, also the MO patients displayed active cortical inter-connections. The intermittent flash stimulation drives the basal EEG rhythm, causing the oscillating network within the resting visual system to adapt themselves to the frequency of stimulation. An idling condition can thus appear for a prevalence of inhibitory mechanisms within the visual system [32]. The checkerboard pattern might generate a disruption of the default visual network, with a specific activation of striatal and extra striatal cortex [6, 7] and a consequent flow of information across the brain regions, which followed a different behavior in the two types of migraine, as was also confirmed by the brain networking analysis.

Brain networking analysis In MA patients, the networking model seemed to be more concentrated within the parietal-occipital cluster of connections, especially under high spatial frequency stimulation, with a more evident segregation of information into the posterior areas. The characteristic path length was in fact shorter in MA patients, as an effect of segregation of connections within the posterior regions. In MA patients, also the centrality of connections was more concentrated into the bilateral parietal-occipital regions, which were mutually linked and clustered. The biological significance of this complex paradigms is quite difficult to be explained, as networking analysis uses innovative concepts of brain connectivity. The present FMRI findings confirmed the well-established pattern of prevalent activation of primary and secondary visual cortex in migraine with aura compared to patients without aura [18, 33]. Our study was focused on innovative EEG analysis, and the acquisition of standard FMRI data in a subgroup of patients was only realized to indicate the brain areas with a different behavior in the two forms of migraine, at least under visual stimulation. In our subgroup of patients, the 2 cpd spatial frequency was able to determine a relevant difference between migraine with and without aura in term of visual cortex activation, thus confirming to be a type of visual stimulation able to reveal subtle differences between the two forms of migraine [6, 7].

Study limitations. The study has many flaws, the most linked with the complexity of the methodological approach. We were not able to merge the connectivity measures into a single model to be applied to single cases, in order to correlate the clinical features as the severity and duration of migraine and the specific aura symptoms with the brain networking pattern. The FMRI was not performed in all cases, for technical problems, so also in this case the association of the EEG connectivity model to the single FMRI feature was not possible.

General comments of results in light of migraine pathophysiology and clinical significance. The brain networking analysis, based on the connectivity models, may represent a way to explain the brain functions and neurological disorders. It can add knowledge about the complex mechanism of migraine, as the different way of brain interconnection may be an epiphenomenon of the altered neuronal excitability affecting migraine brain [34]. The neurophysiological methods traditionally employed to test the different neuronal functioning in migraine, may be implemented by time-related connectivity analysis, useful to confirm that migraine brain has a different way of function even in the inter-ictal phase, and that subtle differences exist between patients with or without aura symptoms perception. The mode of cortical connections characterizing migraine with aura patients, especially in the occipital areas, may be a facilitating factor for the progression of the acute electrophysiological phenomena subtending the perception of aura symptoms [1, 2]. In fact, the different pattern of brain connectivity and networking observed in the two forms of migraine, can be linked with the phenotypical difference consisting in the perception or not of aura symptoms. The patients we selected in the MA group, were exclusively affected by attacks preceded by typical aura symptoms, and we also avoided with particular attention to include patients with minimal visual and sensory symptoms preceding headache in the MO group. However, the future onset of aura symptom in MO patients cannot be excluded, as the mixed forms are very frequent, with a possible overlapping of the neurophysiological pattern of connectivity in the two forms of migraine, as demonstrated by the similarities observed in the EEG in resting state and under visual stimulation. In both migraine groups, the model of transfer entropy under visual stimulation was very different from the control one, indicating a richness of causal connections across the scalp derivations consequent to the visual cortex stimulation, with a common ground of brain hyper-activation that could explain the possible coexistence of the two forms of migraine in the same case, as is commonly observed in clinical practice [35].

## Conclusions

The present results could confirm that migraine with and without aura phenotypes share a common way of brain response to visual stimuli, though a peculiar parietal-occipital cortical inter-connection mode characterizes patients experiencing aura symptoms. The homogeneity of patients for clinical features, indicates that the perception of aura symptoms was the main factor determining the neurophysiological differences between the two migraine groups. The wealth of information exchange in the parietal-occipital regions may be a sign of the peculiar excitability of the visual cortex, a pivotal condition for the manifestation of typical aura. Presently, we have no data to explain the biological basis of this phenomenon, if it may be genetically supported, and the intrinsic and extrinsic factors favoring such different brain connections. The next attempt would be the extraction of a discriminating connectivity parameter, which could be associated to the clinical outcome and possibly to the response to acute and preventive treatments.

## Additional file

Additional file 1: Detailed Brain Networking Analysis. (PDF 3283 kb)
