# Supplementary material for: Brain networking analysis in migraine with and without aura
Source: J Headache Pain. 2017 Sep 29;18(1):98. doi: 10.1186/s10194-017-0803-5 (PMC5622013; doi:10.1186/s10194-017-0803-5)
Supplement: Additional file 1: — Detailed Brain Networking Analysis. (PDF 3283 kb) [file 10194_2017_803_MOESM1_ESM.pdf]

BRAIN NETWORKING ANALYSIS IN MIGRAINE WITH AND WITHOUT AURA

by M. de Tommaso et al.

SUPPLEMENTARY MATERIAL

Contents

Introduction **3**

1 Introdution to Information Theory **7**

1.1 Basics of Information Theory 7

1.2 Application to medicine: nomenclature 8

1.3 Information and Entropy 10

1.4 Transfer Entropy 14

1.5 Strategies for *pf* estimation 17

1.5.1 Method of *variable-width binning* 17

1.5.2 Kernel Density Estimation 20

1.5.3 Darbellay-Vajda partitive algorithm 24

1.5.4 Comparison of the algorithms 26

1.6 Multivariate TE 27

1.7 Cross-Correlation 28

1.8 Synchronization Entropy 31

2 Time-Frequency analysis of signals **35**

2.1 Introduction 35

2.2 Fourier Analysis 35

2.3 Wavelet Analysis 38

2.4 Mother Wavelet 42

2.4.1 Haar Mother Wavelet 45

2.4.2 Mexican Hat 46

2.4.3 Morlet Wavelet 47

2.4.4 Comparison of the mother wavelet 49

3 Causality **51**
3.1 Introduction 51
3.2 Granger Causality: the linear model 52
3.3 Granger Causality: nonlinear model 57
3.3.1 Complete Multivariate Model (MVAR) 61
3.4 Equivalence between Transfer Entropy and Granger Causality 64

4 Brain Networking **67**
4.1 Introduction 67
4.2 Networking Principles 68
4.3 The threshold problem 72
4.4 Caracteristic features of BN 74
4.4.1 Basic Measures 75
4.4.2 Functional Segregation measures 77
4.4.3 Functional Integration measures 81
4.4.4 Small-world Connectivity 83
4.4.5 Motifs of the network 85
4.4.6 Centrality measures 86
4.4.7 Resilience Measures 89

5 EEG Data analysis **93**
5.1 Introduction 93
5.2 Characterization of EEG and *10-20* system 94
5.3 Frequency Bands 96
5.4 Data pre-processing 99
5.5 Potentials evoked by visual stimulation (SVEP): flashing light 99
5.5.1 Analysis of the spectral power 101
5.5.2 Analysis of functional connectivity: Synchronization Entropy 101
5.5.3 Analysis of effective connectivity: Granger Causality 104

5.5.4 Conclusions 106
5.6 Evoked potentials from photic checkerboard 106
5.6.1 Analysis of effective connectivity: Transfer Entropy 108
5.6.2 Analysis of Brain Network 112
5.6.3 Conclusion 125
5.7 Evoked potentials by laser painful stimulation 127
5.7.1 Wavelet Analysis 128
5.7.2 Analysis of effective connectivity: Transfer Entropy 130
5.7.3 Functional Analysis: Synchronization Entropy 134
5.7.4 Brain Network Analysis 138
5.7.5 Conclusion 143

Conclusions **147**

Bibliography **149**

Introduction

Computational neuroscience is the study of brain function in terms of the information processing properties of the structures that make up the nervous system. It is an interdisciplinary science that links the diverse fields of neuroscience, cognitive science, and psychology with electrical engineering, computer science, mathematics and physics.

Computational neuroscience is distinct from psychological connectionism and from learning theories of disciplines such as machine learning, neural networks, and computational learning theory in that it emphasizes descriptions of functional and biological real systems and their physiology and dynamics. These models capture the essential features of the biological system at multiple spatial-temporal scales, from membrane currents, proteins, and chemical coupling to network oscillations, columnar and topographic architecture, and learning and memory and computational models are used to frame hypotheses that can be directly tested by biological and/or psychological experiments.

The term "computational neuroscience" was first introduced in 1985 by Eric Schwartz, who organized a conference in Carmel, California, at the request of the Systems Development Foundation to provide a summary of the current status of a field which until that point was referred to by a variety of names, such as neural modeling, brain theory and neural networks. The proceedings of this definitional meeting were published in 1990 as the book "Computational Neuroscience".

The first open international meeting focused on computational neuro-

science was organized by James M. Bower and John Miller in San Francisco, California in 1989 and has continued each year since as the annual CNS meeting [3]. The first graduate educational program in computational neuroscience was organized as the Computational and Neural Systems Ph.D. program at the California Institute of Technology in 1985.

Research in computational neuroscience can be roughly categorized into several lines of inquiry, from memory and synaptic plasticity to network behavior, from learning behavior to sensory processing. But what is particularly important is the fact that most computational neuroscientists collaborate closely with experimentalists and neurologist in analyzing novel data and synthesizing new models of biological phenomena.

The present line of research is embedded in a particular branch of computational neurosciences called "neuroinformatics", a research field concerning the organization of neuroscience data by the application of computational models and analytical tools. These areas of research are important for the integration and analysis of increasingly large-volume, high-dimensional, and fine-grain experimental data. Neuroinformaticians provide computational tools, mathematical models, and create interoperable databases for clinicians and research scientists: neuroscience, infact, is a heterogeneous field, consisting of many and various sub-disciplines (eg, cognitive psychology, behavioral neuroscience, behavioral genetics and so on), and in order for our understanding of the brain to continue to deepen, it is necessary that these sub-disciplines are able to share data and findings in a meaningful way: Neuroinformaticians facilitate this, combining informatics research and brain research and providing benefits for both fields of science. On one hand, informatics facilitates brain data processing and data handling, by providing new electronic and software technologies for arranging databases, modeling and communication in brain research. On the other hand, enhanced discoveries in the field of neuroscience will invoke the development of new methods in information technologies.

In this context, the improved efficiency of processors and, by consequence, of the calculus velocity has driven computational neuroscience, and "neuroinformatics" in particular, to deal with new possibilities and new tools, such as Granger Causality, Transfer Entropy indicators that, even born in different context (econometrics, wether analysis and so on), has become important landmarks of this research branch and of fundamental utility in extracting neural dynamics from biological data, as every other kind of temporal data analysis.

Moreover, the Brain Networking has become, in the last three years, a fundamental tool to investigate dynamics between biological system, but the present work is, as a matter of fact, probably the first attempt to apply such kind of measures at human brain dynamics in such a massive way, being, so far, only applied to cortical activity of macaques or other species of primates.

If the results of the present analysis will be confirmed in the larger context of Neurology, the tools developed so far will recive a further incentive to be considered not only fundamental physical and mathematical tools, but even important investigation instruments to help medicians and neurologists to investigate and discover real features of brain dynamics.

Chapter 1

Introdution to Information Theory

1.1 Basics of Information Theory

To introduce the most fundamental concepts of information theory (IT), it is first necessary to define the concepts of simple and conditional probability.

Let, therefore, $p(x, y)$ the probability for two events, x and y, to occur simultaneously. If the two events are independent (or "unrelated", such as the simultaneous launch of two dice), then:

$$p(x, y) = p(x) \cdot p(y) \tag{1.1}$$

or better, the probability is equal to the product of the probabilities related to individual events. This, however, is the simplest case and it is not of interest to us. In fact, if we had to calculate the probability of extracting in sequence specific cards from a deck, without the first coat has been reintroduced into it, (1.1) would not be usable. In this case we prefer to define the "conditional probability", or the probability that an event y occur if the event x already occurred:

$$p(y|x) = \frac{p(x, y)}{p(x)} \tag{1.2}$$

From (1.2) we can obtain:

$$p(x, y) = p(y|x) \cdot p(x) \tag{1.3}$$

which defines the "composed probability", that is the generalization of (1.1) to the case of non-independent events.

A simple property we can enunciate and that will be useful later is the following:

$$p(x) \cdot p(y|x) = p(y) \cdot p(x|y) \tag{1.4}$$

If we introduce the inequality signs ($\leq$ and $\geq$), we can also define the concept of "correlation" between two events. We will say, in fact, that

$$p(x|y) \geq p(x) \qquad\qquad (\ or\ \ p(x|y) \leq p(x)\ ) \tag{1.5}$$

if the two events are correlated positively (negatively), or if the occurrence of y makes it more (less) likely the occurrence of x. The converse is also true, ie if $p(x|y) \leq p(x)$, then the occurrence of the x event is favored by the occurrence of y.

It is important to note, for completeness, that the fact that two events are correlated or not does not imply that one of them *causes* the other.

1.2 Application to medicine: nomenclature

The probabilistic concepts expressed so far can be reconsidered and revised in medical field for future convenience. In this case, the composed probabilities seen previously assume different names.

If we denote by M the patient suffering from a given disease, with $\bar{M}$ the healty patient and $T^{\pm}$ the success or failure of the test for the diagnosis of disease which M is a carrier, then

- $P(M)$ is called *incidence rate of the disease*;
- $P(T^-|\bar{M})$ is the *specificity* of a test;
- $P(T^+|M)$ is the *sensitivity* of a test;
- $P(M|T^+)$ and $P(\bar{M}|T^-)$ are the *predictive values* of the test.

Leveraging (1.5) we can say that

$$P(M|T^+) = P(M) \cdot \frac{P(T^+|M)}{P(T^+)} \tag{1.6}$$

It remains to understand what $P(T^+)$ is. It is, obviously, the probability that the test gives a positive result, or better represents the *accuracy* of a test.

Based on the general properties of the probability theory, we will have:

$$P(T^+) = P(M) \cdot P(T^+|M) + P(\bar{M}) \cdot P(T^+|\bar{M}) \tag{1.7}$$

and

$$P(T^+|\bar{M}) + P(T^-|\bar{M}) = 1 \tag{1.8}$$

The probabilities $P(T^+|\bar{M})$ and $P(T^-|M)$ represent the occurrence probability, respectively, of the so-called *false positives* (FP) and the *false negative* (FN).

Putting together equations (1.6), (1.7) and (1.8), we can obtain the expressions for the *predictive values*:

$$P(M|T^+) = \frac{[P(M) \cdot P(T^+|M)]}{[P(M) \cdot P(T^+|M) + P(\bar{M})(1 - P(T^-|\bar{M})]} \tag{1.9}$$

$$P(\bar{M}|T^-) = \frac{[P(\bar{M}) \cdot P(T^-|\bar{M})]}{[P(\bar{M}) \cdot P(T^-|\bar{M}) + P(M)(1 - P(T^+|M)]} \tag{1.10}$$

that are, as it is evident from the definition, not numerically complementary. If, for example, a certain pathology has an incidence rate on the population, $P(M)$, equal to 0.003 % and the carried test presents sensitivity and specificity, respectively, of 0.999 and 0.998 %, the predictive value of the test will be

$$P(M|T^{+}) = 0,6 \rightarrow 60\% \qquad P(\bar{M}|T^{-}) = 0,999 \rightarrow 99,9\%$$

Ultimately, the positive outcome of the test corresponds to a reliability of 60 %, while for the negative outcome it has a reliability of 99.9 %.

That being so, we can move on to define the bases of the *information* physical concept.

1.3 Information and Entropy

Let a generic event x, which the probability $p(x)$ that may occur is associated with. We define as *information*, $I(x)$, corresponding to x, the amount

$$I(x) = -log[p(x)] \tag{1.11}$$

The reasons why we have chosen this particular structure for (1.11) are obvious: the combination of the sign *minus* and logarithm is to indicate that the less likely is the occurrence of the event x, the more the information it will bring with; the contrary happens for very probable events, which bring with them little information. Furthermore, the fact that a probability can not exceed, numerically, the unit value translates into the fact that the information cannot be negative in any case, in agreement with the second law of thermodynamics; in addition, it is defined in such a way that a sure event, $p(x) = 1$, is correlated to any information. $I(x)$, finally, is a monotonic function strictly decreasing and continuous (no “probability jumps”).

Closely related to the concept of information, there is a particular form of entropy, called *information entropy*, which measures the average information content of a given source. Its definition is not unique and depends on the scope and usage of the particular conceptual area. Some definitions, in fact, concieve the information entropy as a measure of the amount of uncertainty present in a random signal, other as a measure of the information contained therein, or again as the minimum descriptive complexity of a random variable, ie the lower limit of data compression without any loss of information: *Kullback entropy* and *conditional mutual entropy* (of which we will not deal with in this work) are an obvious example.

Even for what concerns its operational definition, there is a wide choice of possibilities. However in this line of research we will refer to the concept of information entropy according to Shannon [1].

Be given, therefore, an ordered set $X = \{x_1, x_2, ..., x_n\}$ of events. According to Shannon definition, the information entropy for this set will be:

$$H = \sum_{x \in X} p(x) \cdot I(x) = -\sum_{x \in X} p(x) \cdot log[p(x)] \tag{1.12}$$

where $p(x)$ is the usual probability contained in x and the sum is extended to all elements of the X sequence.

The fact that the proability related to a particular event may also be zero (which would diverge the logarithm) is not a critical point of theory, as the divergence can be easily avoided recalling the basic limit

$$\lim_{x \to 0} x \cdot \log(x) = 0 \tag{1.13}$$

Furthermore, the entropy function shows a maximum in correspondence the probability value $p = 1/2$ (Figure 1.1).

As evident, we have not indicated which basis the logarithm uses, which is also important for the numerical entity of entropy. The different choices,

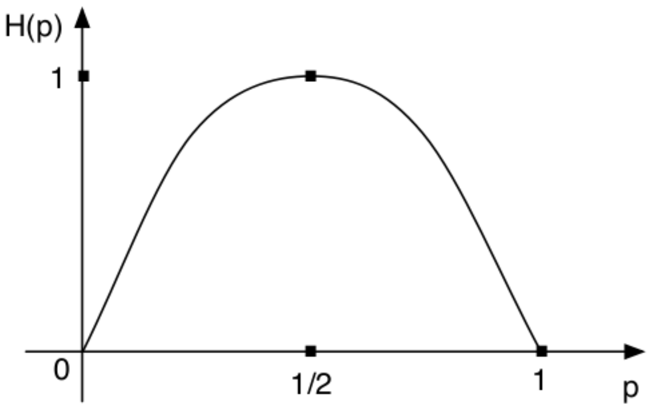

Figure 1.1: general trend of Entropy function $H(p)$

however, are not critical since, as known, it is always possible to change the basis of a logarithm into another: the numerical factor that, in this case, distinguishes the entropy values in different bases is simply the scale factor that binds the *entropy units* in the different logarithm bases. The most commonly used bases are:

1. base 2 $\rightarrow$ unity: *bit*
2. base e $\rightarrow$ unity: *nat*
3. base 10 $\rightarrow$ unity: *Hartley*

If we want to generalize and, instead of a single set of data, *two* set of events are available, $X = \{x_1, x_2, ..., x_n\}$ and $Y = \{y_1, y_2, ..., y_n\}$, the Shannon information entropy will be differently calculated depending on whether the two sets X and Y are "related" (or, more precisely, *not independent*) or not. In the first case we have

$$H_D = -\sum_{x \in X} \sum_{y \in Y} p(x, y) \cdot log[p(x, y)] \tag{1.14}$$

in the second, however, we will have that (1.14) turns as follows:

$$H_I = -\sum_{x,y} p(x,y) \cdot log[p(x) \cdot p(y)] \tag{1.15}$$

According to (1.14) and (1.15), we can define an certain number of combinations that define, in different ways and case by case, the concept of "mutual information" m_i, a measure of the amount of information *shared* by two systems.

A working definition, useful for assessing m_i, is the one that identifies it as the difference between the entropies H_I and H_D of the two systems, or as the difference between the case in which the two systems are independent, and that of the case in which they are not:

$$M_I = H_I - H_D = \sum_{x,y} p(x,y) log \left[\frac{p(x,y)}{p(x)p(y)} \right] \tag{1.16}$$

The mutual information, however, is not entirely suitable for our purposes, because it does not provide anything of *predictive*: it does not anticipate the future dynamics of the systems X and Y, nor provides a probability that the (x_{n+1})-th event can occur on the basis of the sequences X and Y. The reason for this lies in the very structure of (1.16), that is invariant for the exchange $X \leftrightarrow Y$. In addition, the (1.16) gives no information about the directionality of the information flow, if it moves from X to Y or vice versa.

Moreover, the MI is only a "static" measure of the amount of information shared by two time series, completely ignoring the *direction* of the information the same.

This complications can be prevented or by using suitable time shifts of the series X with respect to Y, (the so-called M_{shift}) which allows us to study the variation of MI (and, therefore, the flow of information) in function of the displacement of a time series with respect to the other or, vice versa, according to the Schreiber idea [2], by defining a new type of entropy that somehow "captures" the dynamics of the system based on the changing rate

of (1.15).

1.4 Transfer Entropy

Let supposed the existence of two events series in strict chronological order: $X = \{x_1, ..., x_n\}$ and $Y = \{y_1, ..., y_n\}$, and suppose we want to find any correlation between the two sequences and the "future" element x_{n+1} of the first series. The amount of additional information that would serve to represent (or, equivalently, to *predict*) such a value, is

$$h_1 = -\sum_{x_{n+1}} p(x_{n+1}, x_n, y_n) \cdot log[p(x_{n+1}|x_n, y_n)] \tag{1.17}$$

If the observation value is independent from y_n, then:

$$h_2 = -\sum_{x_{n+1}} p(x_{n+1}, x_n, y_n) \cdot log[p(x_{n+1}|x_n)] \tag{1.18}$$

While the (1.17) is the entropy of the two systems, the (1.18) represents the rate of additional entropy assuming that x_{n+1} is independent of y_n.

We define the *transfer entropy* (hereafter simply TE) as the difference between the two previous amount: $H_2 - H_1$. In other words, the TE is configured as the difference between two different entropy rates: the first related to the amount of information necessary to deduce the element x_{n+1} on the basis of all its known preceeding elements, the second related to the information necessary to deduce x_{n+1} according to *both* the sequences X and Y preceeding it.

Equivalently, the TE can also be understood as a measure of how the uncertainty in the prediction of x_{n+1} on the basis of the only elements of X is reduced by the introduction of Y in the calculation.

Based on the above definition, the TE has a functional form of the type :

$$\begin{aligned} S =& h_2 - h_1 = -\sum p(x_{n+1}, x_n, y_n) log[p(x_{n+1}|x_n)] \\ &+ \sum p(x_{n+1}, x_n, y_n) log[p(x_{n+1}|x_n, y_n)] = \\ =& \sum p(x_{n+1}, x_n, y_n) log \left[\frac{p(x_{n+1}, x_n, y_n)}{p(x_{n+1}, x_n)} \right] \end{aligned} \tag{1.19}$$

According to (1.19), the TE has the minimum value (zero) when the final state is independent of the second time series:

$$p(x_{n+1}|x_n) = p(x_{n+1}|x_n, y_n) \tag{1.20}$$

and may assume at most the unit value. It can not, under any circumstances, be negative, as the relation $p(x_{n+1}|x_n) \geq p(x_{n+1}|x_n, y_n)$ is always true.

The logarithm used in (1.19) is natural or decimal based. If, however, one may wish to take the base 2, the TE would assume a particular value: its units would become the *bit* and the TE the same could be interpreted as the amount of bits that must be extracted from the set X and Y to encode the information carried from x_{n+1}.

The previous formulation, however, is not complete because, as it is obvious, there is no symmetry for the exchange $x \leftrightarrow y$ (or, as we will say from henceforth, for exchange of the two channels, X and Y). We will have, therefore, to reconsider the TE so as to include both of the following cases:

$$T_{Y \to X} \equiv T(Y, X) = \sum \left[p(x_{n+1}, x_n, y_n) log \left(\frac{p(x_{n+1}|x_n, y_n)}{p(x_{n+1}|x_n)} \right) \right] \tag{1.21}$$

$$T_{X \to Y} \equiv T(X, Y) = \sum \left[p(y_{n+1}, x_n, y_n) log \left(\frac{p(y_{n+1}|x_n, y_n)}{p(y_{n+1}|y_n)} \right) \right] \tag{1.22}$$

Finally, by substituting in (1.21) and (1.22) the relations already seen in (1.2)

$$p(x_{n+1}|x_n, y_n) = \frac{p(x_{n+1}, x_n, y_n)}{p(x_n, y_n)}$$
$$p(x_{n+1}|x_n) = \frac{p(x_{n+1}, x_n)}{p(x_n)}$$

we get the equivalent forms:

$$T(Y, X) = \sum \left[p(x_{n+1}, x_n, y_n) log \left(\frac{p(x_{n+1}, x_n, y_n)p(x_n)}{p(x_{n+1}, x_n)p(x_n, y_n)} \right) \right] \tag{1.23}$$

$$T(X, Y) = \sum \left[p(y_{n+1}, x_n, y_n) log \left(\frac{p(y_{n+1}, x_n, y_n)p(y_n)}{p(y_{n+1}, y_n)p(x_n, y_n)} \right) \right] \tag{1.24}$$

In practice, the value of T is a measure of how many digits of the series X can be calculated back to from elements of Y and vice versa. In this way, the TE is better suited than m_i and M_{shift} to determine quantity and direction of the information flow, as it is, always according Shreiber, "a real *directional* and *dynamical* measurement of information transfer" [3].

The relations from (1.21) to (1.24) can be greatly refined by entering informations about the "temporal window" within which the TE is calculated (ie the time interval within which it is assumed that the series of elements x_i are affecting the elements of the Y channel and vice versa, the so-called *lag*, δ), and the *model order*, or the actual number of elements of the set X and Y which are supposed to "influence" the element x_{n+1}.

This informations can be inserted in the previous relations in the following way:

$$T_{k,l}(Y, X) = \sum_{x_{n+1}, x_n^{(k)}, y_n^{(l)}} \left[p(x_{n+1}, x_n^{(k)}, y_n^{(l)}) log \left(\frac{p(x_{n+1}|x_n^{(k)}, y_n^{(l)})}{p(x_{n+1}|x_n^{(k)})} \right) \right] \tag{1.25}$$

$$T_{k,l}(X, Y) = \sum_{y_{n+1}, x_n^{(k)}, y_n^{(l)}} \left[p(y_{n+1}, x_n^{(k)}, y_n^{(l)}) log \left(\frac{p(y_{n+1}|x_n^{(k)}, y_n^{(l)})}{p(y_{n+1}|x_n^{(k)})} \right) \right] \tag{1.26}$$

in which $x_n^{(k)}$ and $y_n^{(l)}$ represent, respectively, the k values preceeding, at regular intervals, x_n and the l value preceeding the y_n elements. The *lag* enters in previous relations when we choose the time distance between (x_n, y_n) and x_{n+1}, different from unit.

If we consider the fact that, generally, k and l are set equal to each other:

$$k = l$$

and that the m values of X and Y are equidistant from each other by the same amount τ (the "delay"), the total width of the time window on which each element of the sum is evaluated is $\Delta + m\tau$. In this context, m is called the *model order*.

If in (1.25) and (1.26) we do not take the sum on time windows, the TE series of values is, in any case, the time behavior of TE between channels X and Y.

As it can be seen from the simple observation of (1.25) and (1.26), the focus of the entire calculus is an estimate of the probability functions (*pf*) $p(x_{n+1}|x_n, y_n)$ and $p(y_{n+1}|x_n, y_n)$.

1.5 Strategies for *pf* estimation

Several methods can be used for the estimation of the *pf*, each with its limitations and its complexity.

Here we will present a few, in order of increasing theoretical and computational complexity.

1.5.1 Method of *variable-width binning*

Let a fixed number m of events for each channel X and Y (m is as always the order of the model) preceeding, equidistant among them of a quantity τ, the

x_t element. The two series of events (simultaneous and temporally ordered) $X_p = \{x_{tm\tau}, ..., x_{t-\tau}\}$ and $Y_p = \{y_{tm\tau}, ..., y_{t-\tau}\}$ constitute the "past" of the x_t event. Let δ (*lag*) the temporal distance between x_t and the most recent series elements representing its past, $x_{t-\tau}$ and $y_{t-\tau}$.

The first step for the estimation of the *pf* plans to allocate the series of events $X_p \otimes Y_p \otimes x_t$, (in this case, $\otimes$ represents the *vectorial concatenation*, or the cartesian product, of the three time series X, Y and x_t) in a $(2m+1)$-dimensional space (called "events space"), whose dimensions are precisely the $2m$-uples of events preceding x_t and x_t the same (fig. 1.2 - A and B panels).

For each combination of $2m+1$ vector elements there corresponds a point in the events space, which starting value is zero and is incremented by 1 everytime the given combination is repeated. As we move forward with the time window ($\delta + m\tau$ samples large) in which it is believed that the channel Y will affect the channel X, the events space starts populating more or less uniformly, depending on whether the channel Y affects or not X (figure 1.2 - B).

In case m equals 1, the events space is three-dimensional, otherwise, in case $m \geq 2$, the events space is an hypervolume of $2m+1$ dimensions. Moreover, in case we want to consider only two channels, X and Y, the analysis is called *bivariate*, and if the "past" of x_t is extended to more than two channels (say X, Y and Z) the analysis (the one we will really apply to our electroencefalographic data) is called *multivariate*, and will be discussed more in detail at the end of this section.

At this point, the conditional probabilities appearing in (1.25) and (1.26) can be estimated in two different ways: by directly summing the probabilities found (whose module is equal to the value the given point assumes with respect to the total number of non-null points of the whole space), or by passing from this to the probability space, which is constructed basing on the first and sharing the same number of dimensions, but with a finite exten-

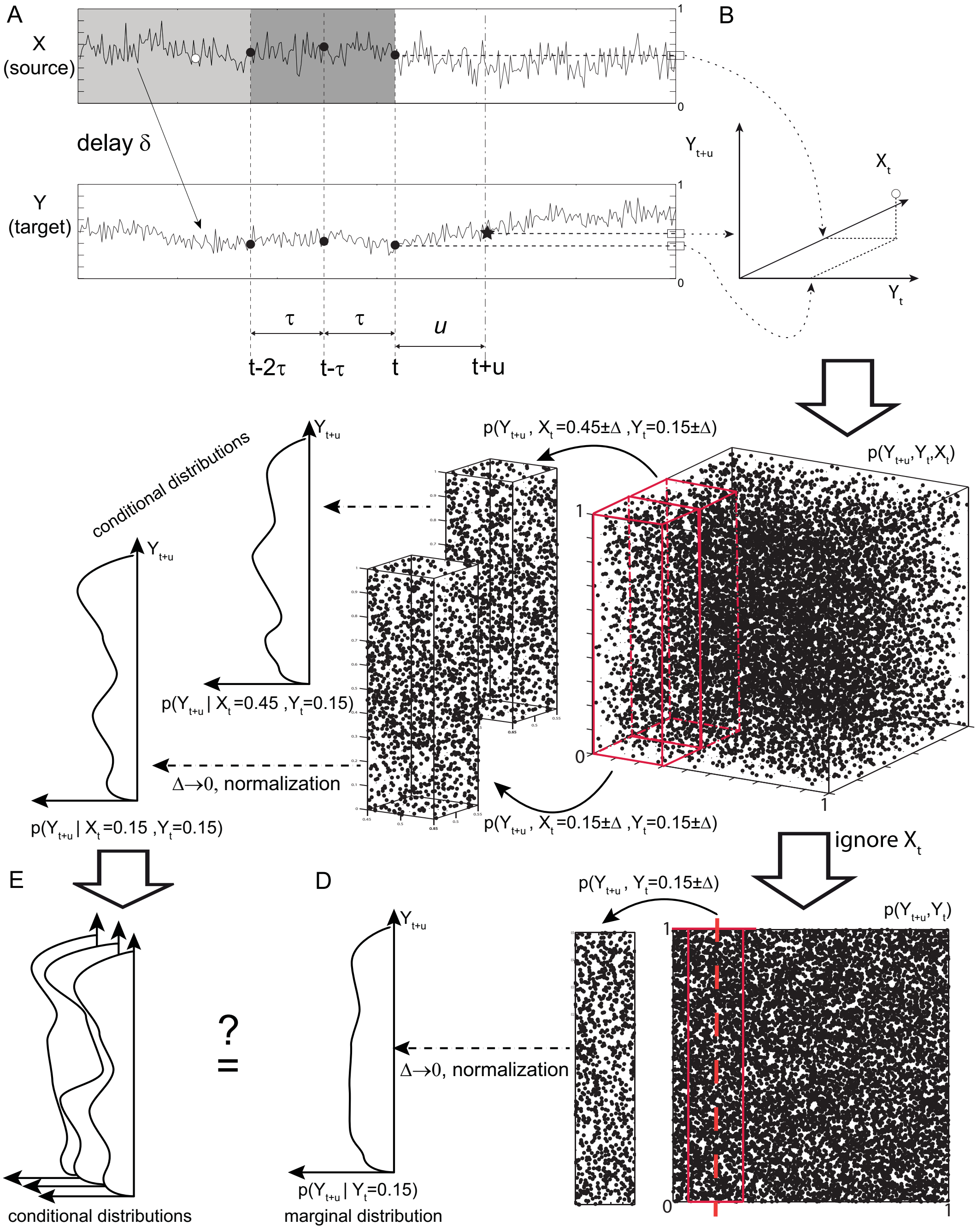

Figure 1.2: General scheme for *pf* estimation [4].

sion and unit volume, the probability being limited to values between 0 and 1 (figure 1.2 - C). Each point of this space represents the fraction of events presenting the probability $p(\vec{X_p}, \vec{Y_p}, x_t)$ to occur. It is preferred, in addition, to reorder the events in this space so that the probability is increasing as one moves from left to right (method of the *ordinal sampling*). The reason for this choice lies the fact that TE is well defined only if the *pf* presents no singular points, or better if there are not points which distributionis is Dirac Delta type, which, in fact, is highly likely to happen in the volume occupied by the points of the sample space.

An equivalent way to do this [4, 5] consists in normailzing the initial data to fit the interval $[0, 1]$, so that the space of events is automatically finished and unit volume. In this case the composed probabilities (1.25) and (1.26) can be derived by setting an interval (*bin*) common to all dimensions, Δ, and considering in turn the basic volumes $(X_p^i \pm \Delta \cdot Y_p^k \pm \Delta)$ and unit height (i and k are indices that run along the space dimensions).

In this case, the composed probablities $p(x_t|\vec{X_p}, \vec{Y_p})$ can be obtained by adding together, at different heights, nonzero elements on the cutting plane identified by the pair $(\vec{X_p}, \vec{Y_p})$, normalizing and reducing to zero the interval Δ. As well, different *pfs* are obtained as a function of X_p and Y_p (figure 1.2 - E). Similarly, the probability $p(x_t, X_p)$ is obtained *marginalizing* the dimension spanned by Y_p (ie, summing over all the Y_p values), no longer working on volumes but on surfaces centered around X_p and large 2Δ (figure 1.2 - D).

1.5.2 Kernel Density Estimation

The Kernel Density Estimation method (KDE) is used to estimate the *pf* by adding together individual distributions centered on each element of the series.

Considering, in fact, the events space of the previous case, we can imagine that in each volume Δ wide, centered on the generic point $\{\tilde{x}_t, \tilde{x}_{t-\tau}, \tilde{y}_{t-\tau}\}$

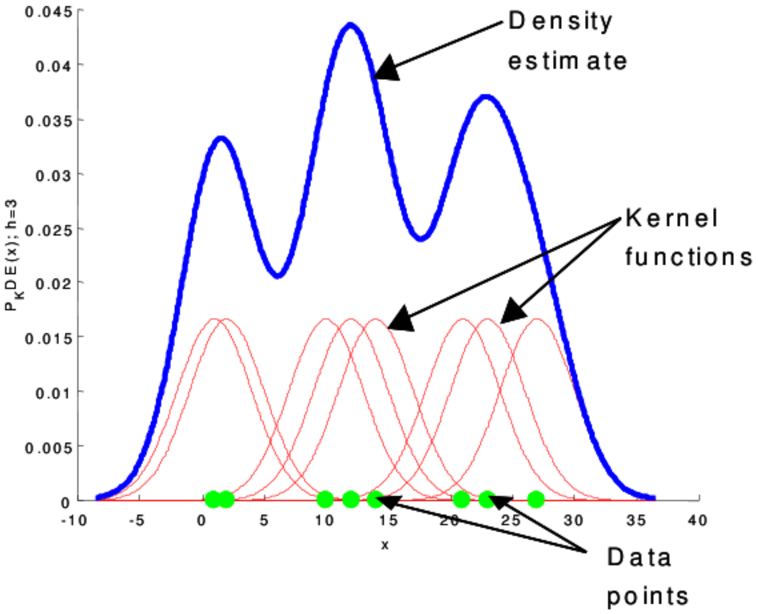

Figure 1.3: the distribution in blue is calculated, by means of KDE method, summing the distributions relative to each data (in red).

of the (three-dimensional) sample space, there are P points, whose distribution is described by generic function $K(x_t, x_{t-\tau}, y_{t-\tau})$ (called *kernel* of the model). K depends not only on space coordinates, but also by a number of parameters, some of which have well-defined values, while others must be chosen *ad hoc*, in such a way as to make the shape of the distribution as close as possible to the real data. In addition, due to issues related to probabilities normalization, it has to show some peculiar features, such as the rapid decrease estranging from the distribution center[1] and must meet the following three conditions:

[1]with relation to this peculiarity, the concept of *distance* is crucial to the estimation of the *pf*, so that the previously seen method of sorting points according to their increasing probability can not be adopted.

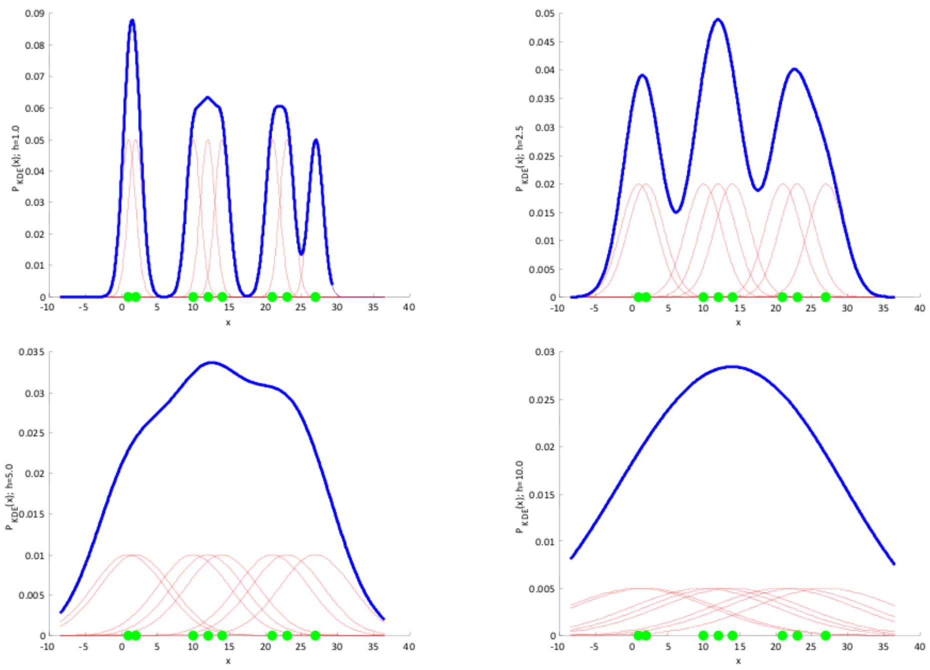

Figure 1.4: in the top left, kernels with poor h values, while at the bottom, kernels with too large h values.

$$(a) \int K(u)du = 1 \qquad (b) \int uK(u)du = 0 \qquad (c) \int u^2 K(u)du \, < \, +\infty \tag{1.27}$$

The probability density is estimated [4] by normalizing the following expression:

$$\begin{aligned} p(\tilde{x}_t, \tilde{x}_{t-\tau}, \tilde{y}_{t-\tau}) \approx \frac{1}{P} \sum_{j=1}^{P} \frac{1}{h_{x_t} h_{x_{t-\tau}} h_{y_{t-\tau}}} \cdot K\left(\frac{\tilde{x}_t - x_{t,j}}{h_{x_t}}\right) \cdot \\ \cdot K\left(\frac{\tilde{x}_{t-\tau} - x_{t-\tau,j}}{h_{x_{t-\tau}}}\right) \cdot K\left(\frac{\tilde{y}_{t-\tau} - y_{t-\tau,j}}{h_{y_{t-\tau}}}\right) \end{aligned} \tag{1.28}$$

where j is the index identifying all points within the elementary volume and $h_{(.)}$ is the characteristic width of the distribution along the axis indicated by the subscript. This width has an universally accepted value [6] equal to

$$h_{(.)} = 1,06\hat{\sigma}P^{-1/5} \tag{1.29}$$

where $\hat{\sigma}$ is the standard deviation of the sample along the direction defined by the subscript of h. This expression for h can be effectively replaced by another [6] which considers, rather than the standard deviation, the *interquartile range* (IQR), or the width of the range containing the middle half of the observed values, assuming that the sample has normal distribution:

$$h_{(.)} = 0,9\alpha\hat{\sigma}P^{-1/5} \quad , \quad \alpha = min\left(\hat{\sigma}, \frac{IQR}{1,34}\right) \tag{1.30}$$

The choice of the h value is of primary importance, because too small values can lead to *pfs* characterized by peaks or singular points, while too high values can lead to poorly differentiated distributions (figure 1.4).

Concerning $K(\cdots)$, indeed, there are several functional forms that can be used [7]; the most common are shown in the following table:

kernel	K(u)
Epanechnikov	$\frac{3}{4}(1-\frac{1}{5}u^2)/\sqrt{5} \quad if \quad \lvert u\rvert < \sqrt{5}$<br>$0 \quad otherwise$
Biweight	$\frac{15}{16}(1-u^2)^2 \quad if \quad \lvert u\rvert < 1$<br>$0 \quad otherwise$
triangolare	$1-\lvert u\rvert \quad if \quad \lvert u\rvert < 1$<br>$0 \quad otherwise$
rettangolare	$\frac{1}{2} \quad if \quad \lvert u\rvert < 1$<br>$0 \quad otherwise$
gaussiano	$\frac{1}{\sqrt{2\pi}}e^{-\frac{u^2}{2}}$
...	...

The most widely used distribution for the kernel is the Gaussian distribution with all its variants (unimodal, bimodal, etc..), which for $h_{(.)}$ tending to zero reduces to a Dirac delta distribution.

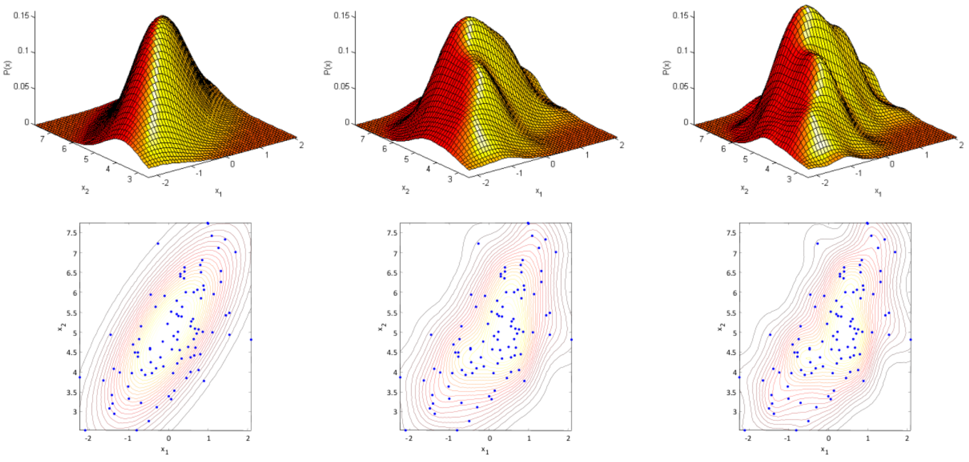

Figure 1.5: left, 100 points distributed according to a generic Gaussian; center and right the same distribution reconstructed with two unimodal Gaussian kernel: the first with $h = 1.06\hat{\sigma}P^{-1/5}$, the second with $h = 0,9\alpha\hat{\sigma}P^{-1/5}$.

It is important to stress one aspect of (1.28): as the *pf* was built as a product of different kernels, one for each axis of the events space independent among them, this does not mean that the variables that are referred to are equivalently independent each other; in the latter case the general form of (1.28) would be of the type:

$$p(x) = \prod_{d=1}^{D} \frac{1}{Ph_{(d)}} \sum_{n=1}^{N} K_d \left(\frac{x_d - x^{(n)}}{h_{(d)}} \right) \tag{1.31}$$

where D is the number of dimensions of the events space and N is the number of points in the elementary volume.

The remaining probability featuring in (1.28) can be calculated starting from $p(\tilde{x}_t, \tilde{x}_{t-\tau}, \tilde{y}_{t-\tau})$ marginalizing, each time, the distribution.

1.5.3 Darbellay-Vajda partitive algorithm

Let's denote the two starting time series, ordered according with the increasing trend of their values, with $U = \{u_1, ..., u_N\}$ and $V = \{v_1, ..., v_N\}$. The

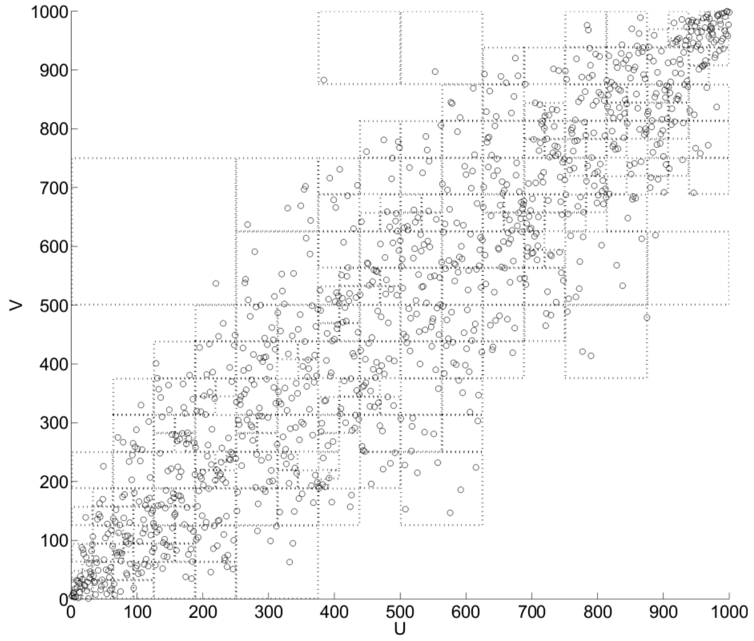

Figure 1.6: D-V partitioning in two dimensions.

three-dimensional space identified by these points and the actual values of the future of one of them can be divided into a certain number of different sizes cubic shape sub-spaces (generally we start with 8 cubes, whose vertices are identified by the points on the axes having probability equal to 0, 0.5 and 1).

Each of these cubes will contain a certain number of points, on which the χ^2 statistic can be calculated to verify the points being or not uniformly distributed in space [4]:

$$s_{\chi^2} = \sum_{i=1}^{8} (M_i - \mu_M) \tag{1.32}$$

in which $\{M_1, ..., M_8\}$ represents the series' points numbers in each cube and μ_M is the total number of points in the probability space divided by 8 (or the number of cubes). If the s_{χ^2} test value is numerically greater than the $\chi^2_{95\%}(7)$ value (5% significance level with 7 degrees of freedom), then the null hypothesis can be rejected, and for each of the eight cubes we proceed to a

further division into eight subcubes in the same way (figure 1.6).

In a recursive way we will reach a point where the null hypothesis can no longer be rejected: the last eight cubes will be considered as a single entity (ie, the last partition has no validity) and we end up with a certain number L of partitions of the probability space, number in which have been deleted partitions not containing points within them.

The (1.25) can, therefore, be rewritten as follows:

$$T_{U \to V} \approx \sum_{k=1}^{L} \frac{n_k}{P} log \left(\frac{n_k n_k^{v_{i-1}}}{n_k^{v_{i-1},u_i} n_k^{v_i,v_{i-1}}} \right) \tag{1.33}$$

with P representing the total number of points in space, n_k the number of points in the k-th partition and $n_k^{v_{i-1}}$, $n_k^{v_i,v_{i-1}}$ and $n_k^{u_i n_k,v_{i-1}}$ representing the number of points in the *whole* probability space having values between the limit values (top and bottom) of the k-th partition with respect to the dimensions indicated in the apex. For example, taking the k-th partition, if the limits of the same are 1 and 5, $n_k^{v_{i-1}}$ represents the number of points throughout the space whose values, independently on the other dimensions, are in the range [1, 5] along the dimension of v_{i-1}.

1.5.4 Comparison of the algorithms

It is possible to compare the results from different algorithms outlined above. Using a series of simulated data [4], it has been proved that all three methods effectively identify the appropriate time interval elapsing between an event in the channel X and the one it “caused” in channel Y (the *lag*, in this case it is equal to 2, as one can see in figure 1.7); also, the calculated TE level is comparable in the first and in the third case, while with the KDE algorithm there was a slight decrease (figure 1.7). This leads us, in the choice of the algorithm to use, to lean towards the former model, both for issues related to the computation time and because it provides both higher mean values of TE than in the two other cases in the time intervals in which there is do an actual causal connection between the two channels.

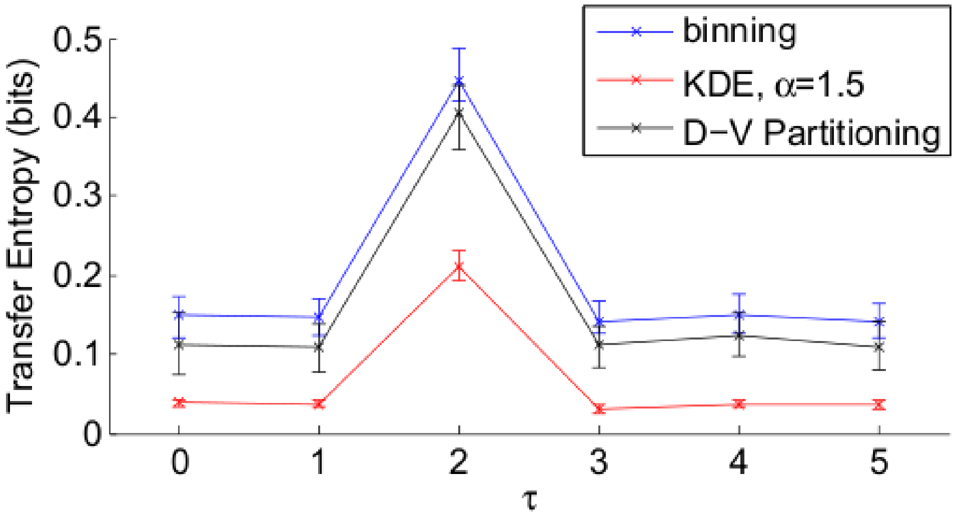

Figure 1.7: comparison between the different algorithms [4]; the error bars represent the interquartile range.

1.6 Multivariate TE

There are cases in which the space-time series constituting a complex system are more than two, such as the inforation flow between various areas of the cerebral cortex. More importantly, these series can interact between them, so that the behavior of each of them is not determined only on the one of another, but on the *net* and contemporary behavior of all the others.

In this case the formulation of the TE outlined here is insufficient, since it does not take into account this effect, but only the relationship between two of them, as if the surrounding universe did not exist (the *bivariate* case).

To overcome this deficiency, the theory must be reformulated in the *multivariate* way, trying to introduce corrections that provide the *net* amount of information exchanged uner the action that other occurring time series can have on that under investigation [8].

Fortunately, the TE formalism outlined here lends well itself to multivaria-

te extension (MVAR), and requires only a redefinition of relation (1.19).

Let therefore X, Y and Z three time series (three channels) that can mutually interact. If we denote, for shorteness of notation, with X^-, Y^- and Z^- their past (vectors whose size depends, as we have already seen, on the model order), with x the *current* value of the series X and with $\oplus$ the *concatenation* operator, then it can be shown that, with a procedure identical to that already seen, one get [9]:

$$\begin{aligned} T_{Y\to X|Z} =& H(x|X^- \oplus Z^-) - H(x|X^- \oplus Y^- \oplus Z^-) = \\ & \sum \left[p(x_{n+1}, x_n, y_n, z_n) log \left(\frac{p(x_{n+1}|x_n, y_n, z_n)}{p(x_{n+1}|x_n, z_n)} \right) \right] \end{aligned} \tag{1.34}$$

with $H(\cdot|\cdot)$ representnig the *conditional entropy*. All other possible combinations of the three (or more) channels can be obtained and calculated with the same modalities seen previously, being the partitive algorithms become richer just of a few more dimensions.

1.7 Cross-Correlation

As we have seen so far, the calculation of TE is intimately linked to the choice of the *lag* parameter, ie, the elapsed time between the current value of the time series under examination and the past of time series that are supposed to influence it.

Obviously, this value may make more or less valid the study, therefore its choice must be made using appropriate tools.

The best way to do so is to perform the calculation of TE varying time to time the *lag* value, so as to obtain the temporal variation of transferred information and, once identified the maximum, to obtain the moments in time where the exchanged information is maximum. Moreover, this is the best way to study the temporal dynamics of information as it makes obvious the temporal changes in interaction between the various time series under

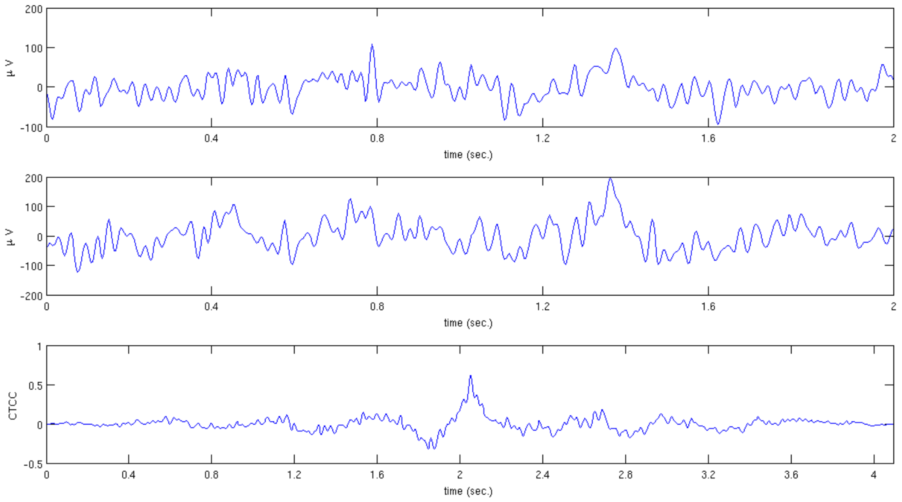

Figure 1.8: trend of the CTCC for two time series: on top, the X series, and in the center the Y serie. Bottom, the CC: the positive peak at about 2 seconds indicates the instant of maximum transfer of information between the two series (a few moments after the synchronization).

investigation. However, this requires a long computation time, especially because it is not known *a priori* the temporal extension of the *lag*, and this inevitably leads to an unnecessary waste of calculation time.

One way to impose constraints on the variability of the *lag* is to calculate the *Cross-Correlation* (CC) between the two time series, defined as the covariance of the two temporally ordered and normalized series X and Y. Formally, it can be considered as the normalized convolution of the two series, which in the case of discretized data can be expressed as:

$$C_{xy}(t) = \frac{\sum_{j=1}^{k} x(t+\tau_j)y(t+\tau_j)}{\sqrt{\left[\sum_{j=1}^{k} x^2(t+\tau_j)\right]}\sqrt{\left[\sum_{j=1}^{k} y^2(t+\tau_j)\right]}} \tag{1.35}$$

where k is the number of *bins* (common to X and Y) in which the two time series were decomposed. In the case of biological series, k can be the

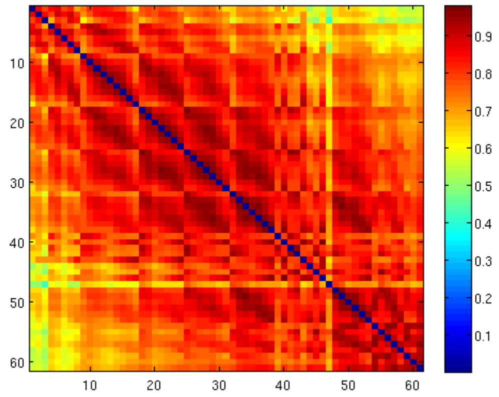

Figure 1.9: CC mean value within a second of all pairs of channels of an EEG. This matrix takes the name of *functional activation map*.

number of stimulations which the patients are submitted to during the EEG recording, therefore the duration of the bin ranges from approximately 1/5 second to 1 second. In this case, the CC feature is called *Cross-Trial Cross-Correlation* (CTCC).

The result of the calculation, for each pair of channels, is a time series itself, twice long as the two original series, whose values lie in the range $[-1, 1]$ and scoring as close as the unity when the two sets are functionally coupled with each other (Figure 1.8). The CC is basically a coefficient comparing between them the shapes of two time series: by making the product of the first series for the second shifted (ie, performing the convolution) we obtain values close to ± 1 if the waveforms are similar to each other, and 0 if they are very different. At the same time, the CC measures the extent to which interval of time the effect of the first time series is translated in the behavior of the second: in this context, the CC proves useful in imposing limits on the *lag* as it allows identification of the time windows in which the series will exchange information, allowing us to choose the appropriate constraints on the *lag* value and reducing the computation time only considering the intervals that *really* are indicative of an interaction between the channels.

Moreover, the output from CC can be averaged over time generating a matrix (or map) of functional activation between all pairs of channels.

Note that the (1.35) is invariant under exchange of the two time series between them, and this makes symmetric the functional activation map (figure 1.9).

1.8 Synchronization Entropy

A second type of entropy that can be evaluated between two channels X and Y is the *Synchronization Entropy* (SE), defined in a similar way to that of Shannon's information (1.12), but referring to the probability that the two time series are *synchronized*.

It is important to note immediately that the synchronization between two time series is not synonymous of their correlation (or *coherence*). However it can be shown [10] that if two systems are synchronized, then certainly they are also correlated (this condition is necessary, but not sufficient).

With this regard, the most appropriate way [11, 12] to define the *phase* of a signal (or a time series) $s(t)$, variable in time, is to introduce a generalization of the signal the same in a complex space, imagining that $s(t)$ is only the *real* part of a *complex* signal, ie composed by a real part and a purely imaginary one. We denote by $\zeta(t)$ this complex function of a real variable (the time, t), which can be rewritten as [13, 14]

$$\zeta(t) = s(t) + is_H(t) = A(t) \cdot e^{i\phi(t)} \tag{1.36}$$

with $S_H(t)$ representing the *Hilbert transform* of the original signal $s(t)$:

$$s_H(t) = \frac{P.V.}{\pi} \int_{-\infty}^{+\infty} \frac{Re[\zeta(\tau)]}{t-\tau} d\tau \tag{1.37}$$

and PV indicating the principal value integral. Once the calculation of (1.36) has been done, the signal amplitude $A(t)$ and its phase $\phi(t)$ are fixed instant

by instant. We note explicitly as the Hilbert transform does not contain additional parameters to be calculated.

From a formal point of view, equation (1.37) can be considered as a convolution of the signal $s(t)$ with the function $1/\pi t$, and basing on the properties of this particular type of product, the Fourier transform of $S_H(\omega)$ of $S_H(t)$ is the product of the Fourier transform of $s(t)$ and $1/\pi t$. This means that, for the frequencies having a physical meaning ($\omega > 0$), the Hilbert transform can be implemented as an ideal filter whose amplitude response is 1 and whose frequency response is equal to $\pi/2$ throughout the entire frequencies band.

It can be demonstrated [15] that (1.36) has physical meaning only in the moment in which the initial signal $s(t)$ is constituted by a narrow band of frequencies: in this case the amplitude $A(t)$ represents the envelope of the signal $s(t)$, and the *instantaneous frequency* is the frequency of the signal presenting a maximum in the spectrum of $s(t)$.

Considering two different time-varying signals, $s_1(t)$ and $s_2(t)$, we can define the *generalized phase difference* among these in the following way:

$$\Phi_{m,n}(t) = [m\phi_1(t) - n\phi_2(t)]_{mod\,2\pi} \tag{1.38}$$

where m and n are two appropriate weights; as it is evident, $\phi_{m,n}$ is defined up to an additive factor 2π. If the two signals, within a certain time interval, are *synchronized*, the distribution of the phase difference will be peacked on a certain value, otherwise it will present itself as uniform. Similarly, if we report the values of the two phases in a two-dimensional diagram, the synchronization will be revealed by the points *clustering* in a specific area of the graph, in spite of what happens if there is no synchronization, when the experimental points tend to either follow separated straight lines or to disaggregate from each other (figure 1.10).

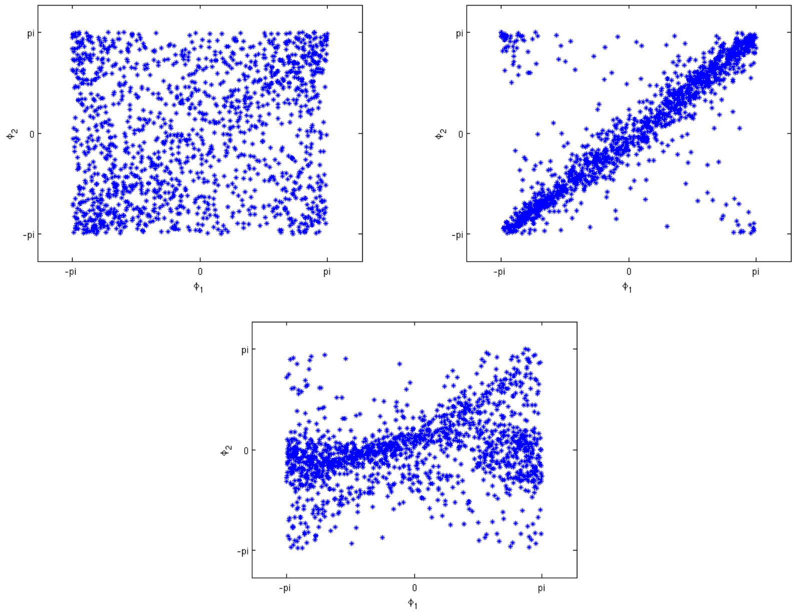

Figure 1.10: effects of synchronization: top, no synchronization; bottom, we can notice a thickening in the central horizontal area with phase difference next to π.

The choice of the two parameters m and n can be made on the basis of considerations about the nature of the two signals. In principle all possible couples should be considered, but if the series are of the same nature, then it is possible to consider both of them as equal to 1.

As already seen for the CC, the SE is invariant too under the inversion of the two sets X and Y, for which the corresponding functional *map*, obtained by associating to each pair of channels the SE value, will be symmetrical (figure 1.11).

The SE can be calculated, as previously mentioned, similarly to (1.12), namely:

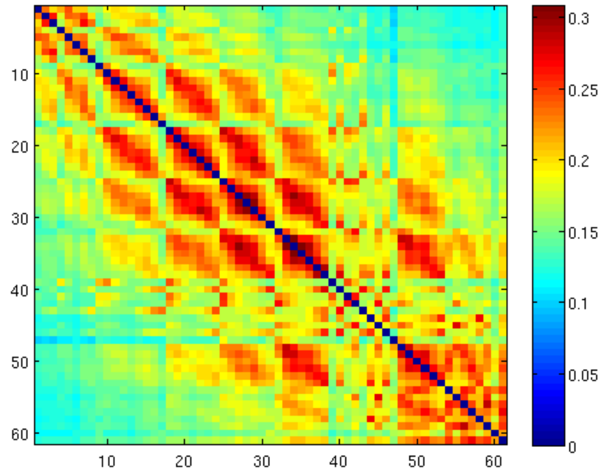

Figure 1.11: SE over all couples of channels for an EEG.

$$SE = -\sum_{i=1}^{N} p(i) log[p(i)] \tag{1.39}$$

in which N represents the number of intervals within which the probability is subdivided, and $p(i)$ is the probability of occurrence for the i-th event (the phase difference, in the specific case). However, differently to what happened for the calculation of the TE, in the present case it is possible to make a correction to the previous relations that cancels the error due to the quantization interval in which the probability is subdivided [16]:

$$SE = -\sum_{i=1}^{N} \hat{p}(i) log[\hat{p}(i)] + \frac{m-1}{2N} \tag{1.40}$$

where $\hat{p}(i)$ represents the frequency with which the i-th combination is present and m is the number of intervals in N having at least one point.

Chapter 2

Time-Frequency analysis of signals

2.1 Introduction

For a time series, the Fourier transform (or series) is a useful tool for very different purposes. One of these, and probably the main one, lies in the fact that its modulus describes the contribution given to the time series from the various frequencies which can be decomposed, while its square module describes the contribution of the various frequencies to the total energy. Very often, in fact, it is of primary importance to know whether certain frequencies in a signal are more active than others, as their presence, absence or different relative amplitude can be related, for example, in pathological changes of the electroencephalographic rhythms of patients suffering from a given disease.

2.2 Fourier Analysis

Given a signal $f(t) \in L^2(\mathbb{R})$ in the time domain, the Fourier Transform (FT) gives a new representation of the signal in the new variable ω domain. The mathematical expression identifying the FT is the following:

$$\hat{f}(\omega) = \int_{-\infty}^{+\infty} f(t)e^{-\omega t}dt \tag{2.1}$$

If the variable ω is considered as a pulse and we require that $\omega = 2\pi\nu$, then we get:

$$\hat{f}(\nu) = \int_{-\infty}^{+\infty} f(t)e^{-2i\pi\nu t}dt \tag{2.2}$$

The function generated by (2.2) depends only on the variable ν, which can be identified as a frequency. The usefulness of such transform resides in the fact that its squared modulus returns, for each frequency, the amplitude of each sinusoidal component constituting the signal, if this is imagined, of course, as the sum of infinite periodic components.

In turn, the individual components are involved with their contribution to the definition of the signal total energy. If we remember, in fact, that for a signal limited in time the total carried energy is

$$E_f = \int_{t_1}^{t_2} |f(t)|^2 dt \tag{2.3}$$

the *Parseval relation* ensure that:

$$E_f = \int_{t_1}^{t_2} |f(t)|^2 dt = \int_{-\infty}^{+\infty} |\hat{f}(\nu)|^2 d\nu. \tag{2.4}$$

$S_f(\nu) = |\hat{f}(\nu)|^2$ is called *energy spectrum* of the signal $f(t)$. Similarly, we obtain for the signal power:

$$P_f = \frac{1}{\Delta T} \int_{-\Delta T/2}^{\Delta T/2} |f(t)|^2 dt = \sum_{\nu} |A_\nu|^2 \tag{2.5}$$

with ΔT representing the time duration of the signal and A_ν indicating the amplitude of the single harmonic component (the sum is extended to all components). The limited time duration of the signal is required from an entropic point of view, since otherwise the energy carried by the signal of infinite duration would diverge. P_F is called *spectral power* and, in analogy with the energy, represents the power carried by the ν-frequency component of the signal.

The analysis of the signal spectrum and its spectral power is one of the first tools in the analysis of biological origin signals and electroencephalographic in particoular: it is widely known how alterations in the relative amplitudes of certain frequencies or their appearance and disappearance often characterize the onset of certain diseases and can also frequently anticipate their debut.

However, the analysis of (2.2) also highlights a limitation of the "classical" spectral analysis: the application of the transform does lose, from an analytical point of view, the functional dependence of time: the signal $f(t)$, in fact, provides the time characterization with infinite resolution (ie, no uncertainty) on the time axis, hiding, at the same time, any information on the frequency content of the signal, on whose axis has null resolution; vice versa, the FT of a signal provides detailed information on the frequency content, but simultaneously presents infinite uncertainty on the time axis. Substantially, the FT gives us informations on the frequencies composing a signal, but does not tell us nor when these components are present, neither if we are dealing with transients or if such a components are present throughout the entire duration of the signal.

Let's consider, for example, the two signals shown below:

$$f_1(t) = \sum_{n=1}^{3} A_n sin(n\omega t) \quad t \in [0,1] \quad e \quad f_2(t) = \begin{cases} A_1 sin(\omega t) & t \in [0,0.3] \\ A_2 sin(2\omega t) & t \in [0.3,0.6] \\ A_3 sin(3\omega t) & t \in [0.6,1] \end{cases} \tag{2.6}$$

On the basis of what has been seen, the Fourier analysis would return, for both signals, the same spectrum: a series of three integer multiples frequencies of ω, the amplitude of each is A_i, and this even though the two signals are deeply different. And this applies to any series of frequencies (see figure 2.1).

This phenomenon must not be surprising: in accordance with the Heisen-

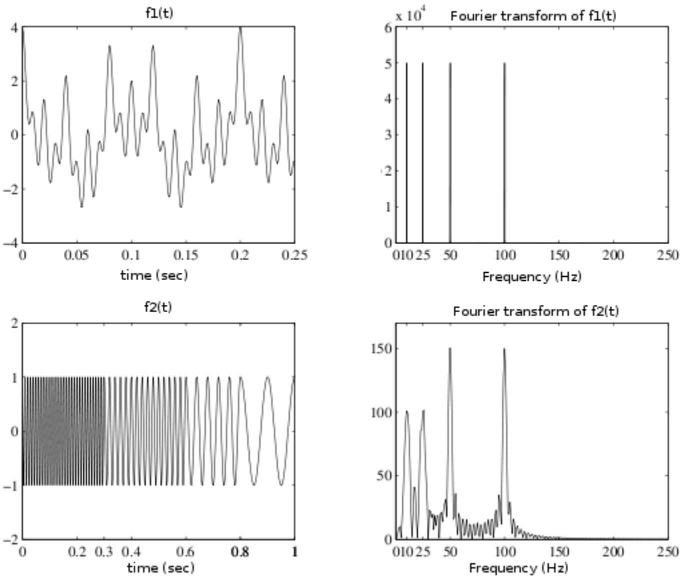

Figure 2.1: the Fourier analysis of two different time series can lead to the same frequency spectrum. In this case, the harmonics have a frequency of 10, 25, 50 and 100 Hz. Arbitrary units for magnitudes (y axis).

berg uncertainty principle, the more precise the frequency characterization of a signal, the poorer the time localization of each component frequency.

By contrast, in biological series (as in many other branches of science) it is important to study the *dynamics* of the time series, highlighting, for example, the occurrence of oscillations or transients following a certain event such as, in case of an electroencephalographic recording, a stimulation.

2.3 Wavelet Analysis

A first way to overcome the problem of temporal localization of a component frequency could be switching from classical Fourier transform to the *windowed* or *short-time Fourier transform* (STFT or WFT) which, instead of acting on the examinated time series through its entire length, only acts

on time windows which can be taken gradually narrower, in such a way to highlight, with the time passing, differences in the frequency composition of the signal.

In this case, the individual elementary components of the signal are extracted by means of the function $W(t) = w(\tau - t)e^{i\omega t}$, where $w(t)$, called "window", is a function with compact support that allows the localization of the temporal component of the signal. The *localized* Fourier transform then becomes:

$$(\mathcal{T}_L u)(\omega, t) = \int_{\mathbb{R}} u(\tau)w(\tau - t)e^{-i\omega t}d\tau = u(t) \star W(t) \tag{2.7}$$

The SWFT, however, has poor inflexiblity because the signal portions are extracted all of the same length, regardless of the frequency content of the signal. Furthermore, the ability of this method to resolve the signal components in the right moment (or at least in compatibility with the uncertainty principle) depends on the windows width which, with the approximating zero, can generate undesirable "edge effects"; it can even require the introduction of "artificial" signals on the external right and left of the window, and this would fit in the window non-real frequencies (in truth, as we shall see, the wavelet transform will suffer from the same problem, but in that case there exist a quantitative parameter taking this phenomenon into account).

A more complex version of SWFT, but that best suits the problem, is to reconsider the form of the window function $w(t)$, its magnitude and its temporal extension.

The basic idea of the *wavelet* functions analysis (meaning "small waves") is to use rectangles of different amplitudes to localize components in the time-frequency plane: more precisely, the localization in frequency decreases logarithmically with the increasing frequency, while the temmporal localization gradually becomes higher (figure 2.2). Differently form Fourier analysis, which passes from a purely temporal representation of the signal to a purely

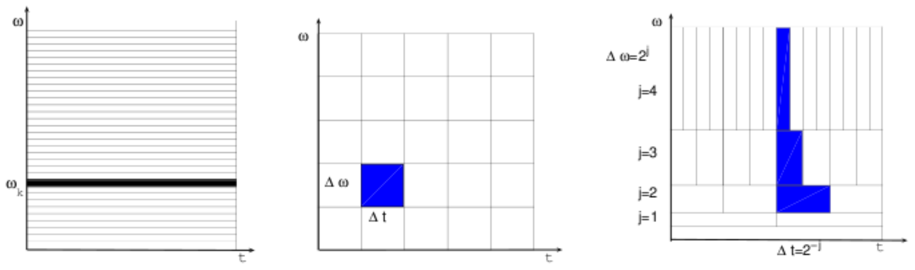

Figure 2.2: the time-frequency plane for the elementary harmonics of Fourier analysis (left), the SWFT (center) and wavelet (right).

frequency representation, the wavelet analysis reaches a compromise fournishing a time-frequency representation.

The time-frequency localization is obtained by replacing, in (2.7), the $W(t)$ function with the so-called *mother wavelet* function, $\psi_{a,b}(t)$, where t is as always the time and a and b are two characteristic free parameters, the first called *scaling parameter* and the second *translation factor*. These two parameters are responsible, respectively, to stretch or shrink the wavelet (and, consequently, to vary the frequency of the wave packet into it) and slide the wave along the time series, in agreement with the definition of convolution product.

From a geometrical point of view, the mother wavelet is a wave packet localized in time and modulated by a Gaussian, whose width is related to the frequency of the wave packet in such a way that, beneath the envelope, a well-defined number of oscillations can take place. In this context, the parameters a and b are particularly important:

- large values of the scale parameter a is equivalent to lengthen the wavelet and its support, decreasing consequently the frequency of the wavelet the same, the reverse being true for low scaling parameters;
- small values of the scale parameter a must match equivalently small

values of the b translation parameter, in such a way to ensure sufficient coverage of the time axis; vice versa, for large values of the scale parameter we can use larger values of b.

Based on these reasons, the *Wavelet Transform* (WT) turns out to be, in analogy with (2.7):

$$\tilde{u}_{a,b} = (\mathcal{T}_W u)(a,b) = \int_{\mathbb{R}} u(\tau) \cdot \frac{1}{\sqrt{a}} \psi^* \left(\frac{t-b}{a} \right) dt = u(t) \star \psi_{a,b}(t) \qquad (2.8)$$

As a result of the convolution of the two time depending functions, the matrix $\tilde{u}_{a,b}$ does not depend on time, and each element is a coefficient quantifying the similarity between the original signal and the wavelet function at specific scale a (equivalent to a certain frequency) and at a specific time shift b.

In the passage from the continuous to the discrete case, typical in computer procedures, one can choose to tie together the two parameters using the integer j in the following way: $a = 2^{-j}$ and $b = ka = k2^{-j}$, with $j, k \in \mathbb{Z}$. On this basis, the $\psi(2^j t - k)$ components correspond, in the time-frequency plane, to rectangles of variable size $2^{-j} \times 2^j$ (figure 2.2).

The main advantage of the wavelet transform usage is the fact that provides a *multiscale decomposition* of the signal: for each scale j, the signal is decomposed into elementary components whose frequency content increases with the scale. In general, the multiscale decomposition of a signal consists in a poorly accurate mean value of the signal at a given scale (content at low frequencies) with more details calculated at more accurate scales (high frequency content). If the signal varies slowly, the details are not important to reconstruct the signal, so the multiscale representation provides a natural tool for the compression: it is sufficient to overlook details below a certain threshold and transmit only the most significant ones. The same technique can be used to reduce noise. On the other hand, the most significant details provided by the wavelet analysis correspond to the areas where the signal

has large variations, for example in correspondence of a peak: this causes the wavelet analysis to be successfully applied in the analysis of signals where you need to extract information about the " geometrical" stuctures of data.

Finally, the mothers wavelet (or "bases") can be used for the representation and compression of integro-differential operators: this allows to build efficient numerical methods for the solution of integral equations or of partial derivatives. Several of these applications are discussed in [17], where a detailed bibliography is also given.

2.4 Mother Wavelet

The mother wavelet function must have specific characteristics defining it uniquely. As we said, it must be a function with a compact support to the very principle of localization of component frequencies.

Second, it must meet a number of specific mathematical requirements, such as presenting a Fourier transform $\hat{\psi}(\omega)$ leading to a limited c_ψ parameter:

$$c_\psi = \int_0^{+\infty} |\hat{\psi}(\omega)|^2 \frac{d\omega}{|\omega|} < +\infty \tag{2.9}$$

For this condition it is sufficient that the wavelet is such that:

$$\int_{\mathbb{R}} \psi(t)dt = 0 \tag{2.10}$$

This consideration arises from the fact that, as in the case of Fourier transform, for the WT too it must be possible to trace back the energy spectrum of the signal, but in a localized way compared to the FT, which represents, in some way, the time integral.

Considering, in fact, both the transform (2.8) and its inverse

$$u(t) = \frac{1}{c_\psi} \int_{-\infty}^{+\infty} \int_0^{+\infty} \left[\tilde{u}_{a,b} \cdot \frac{1}{\sqrt{a}} \psi\left(\frac{t-b}{a}\right)\right] \frac{dadb}{a^2} \tag{2.11}$$

we can firstly find that there is no information loss in the passage from $u(t)$ to $\tilde{u}_{a,b}$ and vice versa, and that we have, for the Parseval relationship:

$$\int_{-\infty}^{+\infty} |u(t)|^2 dt = \frac{1}{c_\psi} \int_{-\infty}^{+\infty} \int_{0}^{+\infty} |\tilde{u}_{a,b}|^2 \frac{dadt}{a^2} \tag{2.12}$$

ie, there is no energy information loss of the signal passing from the time domain to the time-frequency plane, but just its redistribution in correspondence of the activation time intervals and around the component frequencies of the signal is composed with, defined by the $\tilde{u}_{a,b}$ coefficients of the matrix.

This is of paramount importance for our studies: for stationary signals the using of the Fourier transform arised because the information on the dynamics of the signal and the time variation of the spectral power is completely contained in the phase of the transform, and it is completely lost when we take the module, used for the power calculation. Instead, non-stationary signals such as most of the biological series, do not lend themselves to such a loss, and need a time localization of the energy spectrum.

Another feature that the mother wavelet can own (although it is not strictly necessary) is that the higher moments (at least the second, as we are going to see in the next section) are all zero:

$$\int_{\mathbb{R}} t^n \psi(t) dt = 0 \tag{2.13}$$

or that, at least, the first two moments are zero: this aspect is conencted to the best resolution that a wavelet presents at high frequencies with the increasing number of vanishing moments (see below). Furthermore, it must be fullfilled the condition

$$|\psi(t)| < \frac{C}{1 + |t|^p} \tag{2.14}$$

with $C > 0$ and $p \in \mathbb{N}$. Moreover, a feature that the mother wavelet should possibly have is to be "localized" both in the frequency domain and in time.

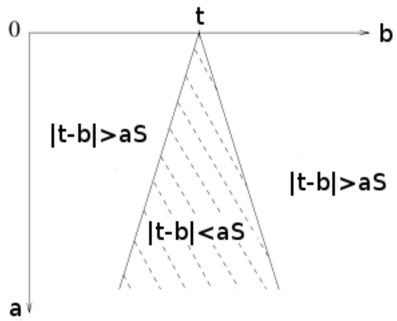

Figure 2.3: the cone of influence of abscissa τ is formed by the points of the plane for which the support of $\psi_{a,b}$ intersects $t = \tau$

Before we can browse the most commonly used mothers wavelet, it is imperative to introduce the concept of *cone of influence* of a mother wavelet. As we have seen, the convolution between two signals requires the wavetet mother to be translated, with all its support (say, $[-S, S]$), along the signal to be analyzed. However, at the initial and final eges of the signal, the wavelet support can easily (and, as a matter of fact, it does) exceed that of the signal, thus causing unwanted edge effects. These effects can be analytically treated extending the signal out of its cradle, for example by assuming the periodicity of the signal outside of its support, or performing a mirroring of the same. These corrections, however, do not eliminate artifacts that can occur, but their weight can be evaluated considering the *cone of influence* of the transform.

Let's imagine, therefore, to consider the generic point τ of the time series. The cone of influence of this point is defined in the time-frequency plane as the set of all points in the plane such that the point τ is content in the mother wavelet $\psi_{a,b}(t) = a^{-1/2}\psi((tb)/a)$ support. Being the latter equal to $[b - aS, b + aS]$, the cone of influence of τ is analytically defined as

$$|b - \tau| \leq Sa \tag{2.15}$$

Consequently, if b is located in the cone of influence of τ, then the wavelet

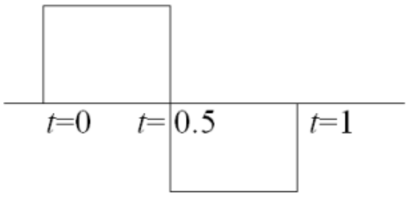

Figure 2.4: Haar wavelet

transform of the signal depends on the value of b in the neighborhood of the point τ (Figure 2.3). The envelope of all the cones of influence of individual points indicates what are the points of the time-frequency plane that are mostly influenced by edge effects, and sholud, in this way, be appropriately considered basing on the assumptions made on the aignal analytical extension.

Let us now consider a series of wavelet which, generally, the analysis is performed.

2.4.1 Haar Mother Wavelet

The Haar wavelet is the simplest among the mother wavelet that is possible to use. It's defined as follows:

$$\begin{cases} 1 \quad if \quad t \in [0, 1/2] \\ -1 \quad if \quad t \in [1/2, 1] \end{cases} \tag{2.16}$$

(see Figure 2.4). It is simply a high-pass filter having the advantage of being simple to implement, it's a fast algorithm for the WT, is reversible, compact, real and odd. However, this simplicity is balanced by obvious disadvantages, the first of which lies in the fact that in nature there are very few examples of rectangular signals, and the lack of regularity of the mother wavelet could generate a number of artifacts in the calculation of matrix elements of $\tilde{u}_{a,b}$. Moreover, it is easy to verify that only the first moment of (2.13) is zero.

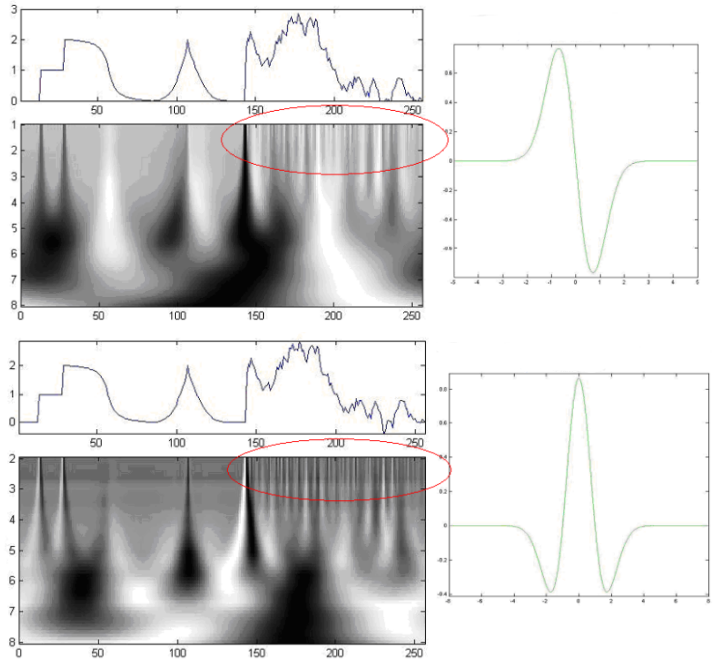

Figure 2.5: above, the WT with the first derivative order of the Gaussian filter (antisymmetric); bottom: WT relative to the second derivative (symmetric); it is evident the greater detail at high frequencies for the second wavelet.

2.4.2 Mexican Hat

It is possible to demonstrate that any p-order derivative of a Gaussian function has the characteristics previously seen to be considered a mother wavelet. In addition, it can be shown that in this case all the moments up to p-th vanish.

Starting from this principle, we can construct an entire family of mother wavelet whose elements are characterized by the growing derivative order, each having higher resolution at higher frequencies. If we observe, in fact, figure 2.5, we can notice how, with the passage of the derivative order from the first to the second, the resolution at high frequencies for the time series shown in the top -common to both analyses- increases (area circled in red).

More in detail, it is evident the way in which the wavelet of the first and second order are odd and even respectively: this is reflected in the way in which the first order is more sensitive to only increment or decrement of the signal, while the second, more accurate, better distinguishes the speed with which the signal varies over time. For this reasons, the second order derivative is preferred to the first (and to any succeeding, both even and odd).

So, starting from the Gaussian function, with $\sigma = a^2/2$

$$F_\sigma(t) = \frac{1}{2\sqrt{\pi\sigma}} e^{-\frac{t^2}{4\sigma^2}} \tag{2.17}$$

the corresponding mother wavelet is

$$\psi_2(t) = \frac{d^2 F_\sigma}{dt^2} = \left(\frac{t^2}{4\sigma^2} - \frac{2}{\sigma} \right) F_\sigma(t) = \frac{e^{-\frac{t^2}{4\sigma}} (t^2 - 2\sigma)}{8\sigma^2\sqrt{\pi\sigma}} \tag{2.18}$$

Because of its shape, such a wavelet is called *Mexican Hat* (or "sombrero", see figure 2.5, bottom). As it is evident since the previous relation, it can be considered as the difference between two Gaussian filters of different scale, divided by the scale difference itself.

In consequence of the above mentioned Parseval relation, the *localized* spectral energy density for a Mexican Hat is given by the relation

$$\int_{-\infty}^{+\infty} \int_{0}^{+\infty} 2|\tilde{u}_2|^2 \frac{d\sigma dt}{\sigma} \tag{2.19}$$

A further step forward for this class of wavelet consists in making the functions ψ_2 complex, for example using the Hilbert transform. However, when it becomes necessary to switch to a complex formulation, we prefer to rely on other mother wavelets families.

2.4.3 Morlet Wavelet

The Morlet wavelet, complex, is one of the most used for the time-frequency analysis of signals. It consists of a wave train of central frequency z_0 modulated by a Gaussian whose width is z_0/π:

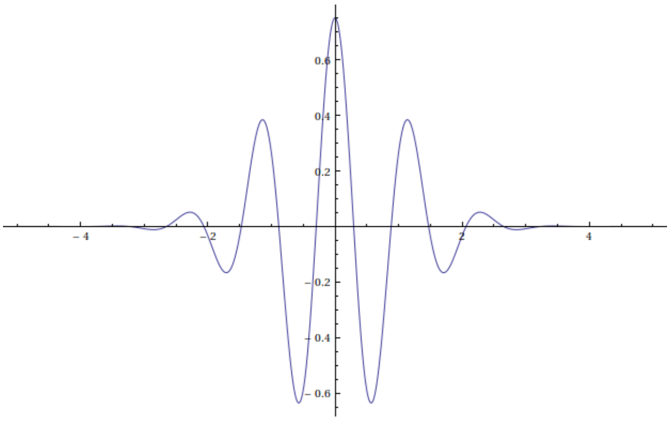

Figure 2.6: the real component of the Morlet wavelet

$$\psi_{z_0}^{M}(t) = c_\psi(e^{2i\pi t} - e^{-\frac{z_0^2}{2}})e^{-2\pi^2 t^2/z_0^2} \tag{2.20}$$

The factor $e^{-z_0^2/2}$ is called "correction factor", as it is used to correct the non-zero mean of the complex sine wave. c_ψ is the normalization coefficient that is not uniquely determined, but related to the value of z_0 that, in addition to defining the central frequency, also controls the number of oscillations within the package. The choice usually accepted for z_0 is 5 or 7, as for these values (larger than 5) the correction factor is very small and can be approximated to zero. In the following table, we show some pairs (z_0, c_ψ) used in literature:

z_0	4	5	7	10
c_ψ	1.1676	1.4406	1.9955	2.8353

Similarly to the previous case, the power spectral density of the signal can be expressed by means of the Parseval relation:

$$\int_{-\infty}^{+\infty}\int_{0}^{+\infty}\frac{1}{2}|\tilde{u}_M|^2\frac{d\omega dt}{\omega} \tag{2.21}$$

with $\omega = 1/a$ and $\tilde{u}_M$ representing the wavelet transform of the signal.

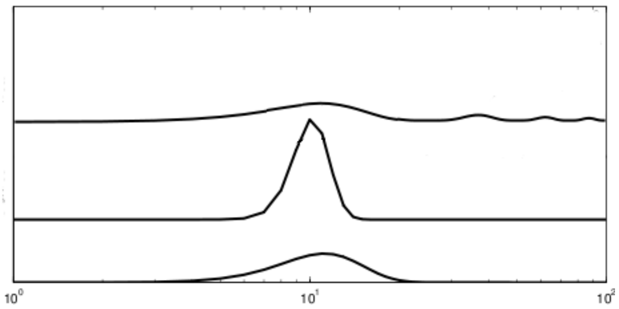

Figure 2.7: frequency spectrum of the different mother wavelet: Haar (top), Morlet (middle), Mexican hat (bottom). In abscissa the frequency is indicated, and the ordinate units are arbitrary.

Since this is a complex wavelet, the transform will be composed by a real part and an imaginary part: the first provides information on amplitude variation of frequencies that make up the signal, the second will describe the phase variation of the components themselves.

2.4.4 Comparison of the mother wavelet

The choice of the mother wavelet to be used to perform the transform is subject to the specific utility of the result and the characteristics the single wavelet.

A first comparison between them can be carried out considering the Fourier spectrum of the wave packet. The Haar mother wavelet, for example, presents a frequency spectrum that is not limited as a consequence of non regularity at the extremes of the support (figure 2.7). In contrast, the frequency spectrum of the Morlet and Mexican hat has a limited variability and a rapid decrease, which implies a better selectivity in frequency. From this point of view the Morlet is favored compared to the Mexican hat to have a narrower band, even if the latter presents a better location performances in the time domain.

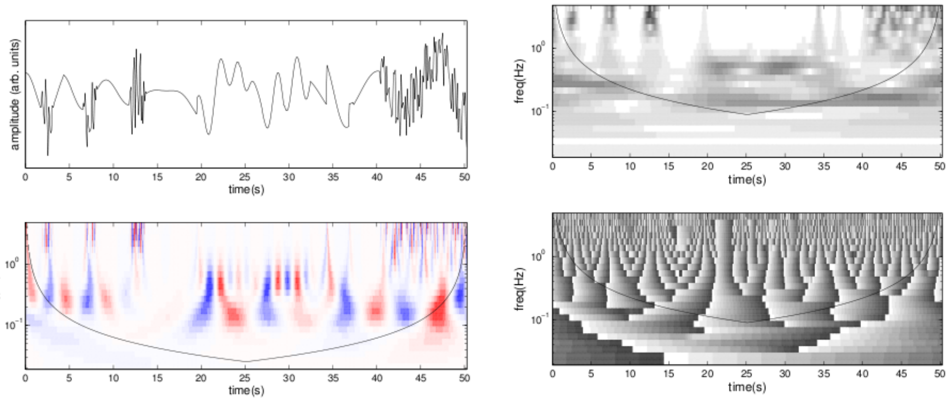

Figure 2.8: top left panel:the signal being analyzed; in the bottom left, the wavelet transform with the Mexican hat at the second order; right top panel: the WT with the Morlet wavelet for the amplitudes; right bottom: Morlet WT for the phase ($z_0 = 5$). In all cases the cone of influence is reported (dark line).

A second comparison between the wavelets can be conducted considering the cone of influence of each of them. As visible in figure 2.8, the Morlet wavelet has a wider cone with respect to the Mexican hat, as a consequence of the fact that the Morlet capture a greater number of oscillations with respect to the Mexican hat at fixed frequency, which covers only 1.5 obscillations.

A final comparison can take place according to the shape of the wavelet. The Mexican hat has little periodicity (a central maximum and two minima at the two sides of the support) and consequently a more marked tendency to highlight local maxima and minima when these are not rapidly succeeding: as already said, in fact, the transform coefficients represent the level of similarity between the mother wavelet and the fragment of analyzed signal. The Morlet wavelet, at the contrary, presents a strong periodicity and appears to be more suited to highlight fast sequences of maxima and minima, at the cost of lower temporal resolution.

Chapter 3

Causality

3.1 Introduction

As we saw earlier, it is possible to assess whether and how two or more time series share information or exhibit similarity of different nature. In the section dedicated to the TE, for example, we understood how to study, starting from the statistical data, the information flowed from a series X to a series Y that have been recorded *simultaneously*. We are now going to understand if we can not only study what happened between two time series, but even if we can go further and try to *predict* the trend of a time series on the basis of its own previous behavior and on the basis of what happens in one or more contemporary time series.

The first attempt of this kind is due to Clive Granger [24], who in a pioneering article of 1967, awarded two years later with the Nobel Prize for Economics, proposed a vector autoregressive model (VAR) to study the mutual influences between the financial markets. Although it was born in the economic sphere, the model of *Granger Causality* (or G-causality, GC) can also be extended to other areas including the dynamics of nonlinear systems [25, 26] and neuroscience [27], helping us to understand what are, for example, the causal relationships between heart rate and blood arterial pressure or between heart rate and respiratory rate [28] and so on.

In recent times, the development of computer technology and the increase in computational power of computers let it possible to extend the scope of the GC until the nonlinear study of complex systems consisting in a large number of time series, which are, for example, recordings of electrical potentials on the scalp, thus opening the door to the numerical study of information dynamic in the cerebral cortex [29].

3.2 Granger Causality: the linear model

To fully understand the vector autoregressive model proposed by Granger [24], it is necessary to introduce, since the beginning, the formalism that we will use from now on. Let $\{\bar{x}_i\}_{i=1..N}$ and $\{\bar{y}_i\}_{i=1..N}$, two time series of data simultaneously measured, with the same number N of samples. From here on we will assume that, for our model to be valid, the two time series are stationary: this means that we have to deal with constant mean and variance signals, and the covariance of any pair os segments belonging to the signal depends only on their relative distance in the signal itself.

Let us consider now the integer m, that will be called, as for the TE, *order of the model*, and the integer k, which can take values from 1 to the integer M = N$-m$. We will denote by x^k the $(k+m)$-th element of the initial series, $\bar{x}_{k+m}$. Since m is related to the "width" of the considered window to calculate the causality, x^k is the k-th element after the considered time window. The same holds for the second series. Finally we define the series $\mathbf{X}^k = (\bar{x}_{k+m-1}, ..., \bar{x}_k)$ and $\mathbf{Y}^k = (\bar{y}_{k+m-1}, ..., \bar{y}_k)$ as the "past" of the elements x^k and y^k respectively. Since k ranges from 1 to M, depending on the model order we can consider M realizations of the stochastic variables $(x, y, \mathbf{X}, \mathbf{Y})$. The VAR model provides, at this point, the introduction of the following linear system

$$\begin{cases} x = \mathbf{W_{11}} \cdot \mathbf{X} + \mathbf{W_{12}} \cdot \mathbf{Y} \\ y = \mathbf{W_{21}} \cdot \mathbf{X} + \mathbf{W_{22}} \cdot \mathbf{Y} \end{cases} \tag{3.1}$$

where $\{\mathbf{W}\}$ is a set of four real m-dimensional vector whose values will be derived from the actual time series data.

The system can be solved with respect to the set $\{\mathbf{W}\}$ by means of least squares techniques, giving [30]:

$$\begin{pmatrix} \mathbf{W_{11}} \\ \mathbf{W_{12}} \end{pmatrix} = \tilde{A}^{-1} \begin{pmatrix} \mathbf{T_{11}} \\ \mathbf{T_{12}} \end{pmatrix} \tag{3.2}$$

and

$$\begin{pmatrix} \mathbf{W_{21}} \\ \mathbf{W_{22}} \end{pmatrix} = \tilde{A}^{-1} \begin{pmatrix} \mathbf{T_{21}} \\ \mathbf{T_{22}} \end{pmatrix} \tag{3.3}$$

in which the operator $\tilde{A}$ identifies the block matrix

$$\tilde{A} = \begin{pmatrix} \Sigma_{xx} & \Sigma_{xy} \\ \Sigma_{yx} & \Sigma_{yy} \end{pmatrix} \tag{3.4}$$

The elements of the Σ matrix, and that of the vectors $\mathbf{T}$, are obtainable from the actual elements of the series in the following way:

$$[\Sigma_{xx}]_{\alpha\beta} = (X_\alpha, X_\beta) = \frac{1}{M}\sum_{k=1}^{M} X_\alpha^k X_\beta^k, \qquad \alpha, \beta = 1, ..., m \tag{3.5}$$

$$[\Sigma_{xy}]_{\alpha\beta} = (X_\alpha, Y_\beta) = \frac{1}{M}\sum_{k=1}^{M} X_\alpha^k Y_\beta^k, \qquad \alpha, \beta = 1, ..., m \tag{3.6}$$

$$[\Sigma_{yx}]_{\alpha\beta} = (Y_\alpha, X_\beta) = \frac{1}{M}\sum_{k=1}^{M} Y_\alpha^k X_\beta^k, \qquad \alpha, \beta = 1, ..., m \tag{3.7}$$

$$[\Sigma_{yy}]_{\alpha\beta} = (Y_\alpha, Y_\beta) = \frac{1}{M}\sum_{k=1}^{M} Y_\alpha^k Y_\beta^k, \qquad \alpha, \beta = 1, ..., m \tag{3.8}$$

while for the vectors $\mathbf{T}$:

$$[\mathbf{T}_{11}]_\alpha = (x, X_\alpha) = \frac{1}{M}\sum_{k=1}^{M} x^k X_\alpha^k, \qquad \alpha = 1, ..., m \tag{3.9}$$

$$[\mathbf{T}_{12}]_\alpha = (x, Y_\alpha) = \frac{1}{M}\sum_{k=1}^{M} x^k Y_\alpha^k, \qquad \alpha = 1, ..., m \tag{3.10}$$

$$[\mathbf{T}_{21}]_\alpha = (y, X_\alpha) = \frac{1}{M}\sum_{k=1}^{M} y^k X_\alpha^k, \qquad \alpha = 1, ..., m \tag{3.11}$$

$$[\mathbf{T}_{22}]_\alpha = (y, Y_\alpha) = \frac{1}{M}\sum_{k=1}^{M} y^k Y_\alpha^k, \qquad \alpha = 1, ..., m \tag{3.12}$$

where (,) represents an inner product.

As one can see, the dimensionality of matrices and vectors in question increases with the order of the model, and with this latter also increases the accuracy in the estimation of the error (this is an autoregressive model, so error must exist and must be different from zero) and its absolute value. If we denote by ϵ_{xy} and ϵ_{yx} such errors, their form appears to be [30]:

$$\epsilon_{xy} = Var\left(x - \mathbf{W_{11}} \cdot \mathbf{X} - \mathbf{W_{12}} \cdot \mathbf{Y}\right) = \frac{1}{M}\sum_{k=1}^{M}(x^k - \mathbf{W_{11}} \cdot \mathbf{X}^k - \mathbf{W_{12}} \cdot \mathbf{Y}^k)^2 \tag{3.13}$$

$$\epsilon_{yx} = Var\left(y - \mathbf{W_{21}} \cdot \mathbf{X} - \mathbf{W_{22}} \cdot \mathbf{Y}\right) = \frac{1}{M}\sum_{k=1}^{M}(y^k - \mathbf{W_{21}} \cdot \mathbf{X}^k - \mathbf{W_{22}} \cdot \mathbf{Y}^k)^2 \tag{3.14}$$

in which $Var()$ represents the variance operator. This model hypotizes, of course, that both sets $\mathbf{X}^k$ and $\mathbf{Y}^k$ contribute to "cause", within a given range, the trend of the variables x and y^k. If we forget for a moment this cross-dependence, then the model, simply called *autoregressive* (AR), would provide that

$$\begin{cases} x = \mathbf{V_1} \cdot \mathbf{X} \\ y = \mathbf{V_2} \cdot \mathbf{Y} \end{cases} \tag{3.15}$$

In this case, the least square method would return the results

$$\begin{cases} x = \mathbf{V_1} \cdot \mathbf{X} \\ y = \mathbf{V_2} \cdot \mathbf{Y} \end{cases} \tag{3.16}$$

with

$$\begin{pmatrix} \mathbf{V_1} \\ \mathbf{V_2} \end{pmatrix} = \begin{pmatrix} \Sigma_{xx}^{-1} & 0 \\ 0 & \Sigma_{yy}^{-1} \end{pmatrix} \begin{pmatrix} \mathbf{T_{11}} \\ \mathbf{T_{22}} \end{pmatrix} \tag{3.17}$$

As a result, the estimated variance of $x - \mathbf{V_1} \cdot \mathbf{X}$ and $y - \mathbf{V_2} \cdot \mathbf{Y}$, that we denote with ϵ_x and ϵ_y (representing the prediction error of x^k and y^k on the basis of the knowledge *only* of their past) generates the following results:

$$\epsilon_x = Var\left(x - \mathbf{V_1} \cdot \mathbf{X}\right) = \frac{1}{M} \sum_{k=1}^{M} (x^k - \mathbf{V_1} \cdot \mathbf{X}^k)^2 \tag{3.18}$$

$$\epsilon_y = Var\left(y - \mathbf{V_2} \cdot \mathbf{Y}\right) = \frac{1}{M} \sum_{k=1}^{M} (y^k - \mathbf{V_2} \cdot \mathbf{Y}^k)^2 \tag{3.19}$$

If the prediction error ϵ_{xy} is found to be smaller than ϵ_x, then we could say that considering *both* the series helps to predict the future of x^k better than just considering only its past. The same is true for the pair ϵ_{yx} and ϵ_y. It is said that the signal $\{\bar{y}_i\}$ has a causal influence or *Granger-causes* the set $\{\bar{x}_i\}$ at the order m.

One way to quantify this causality is to compare among them the prediction errors: we can introduce two parameters, $c_1 = \epsilon_x - \epsilon_{xy}$ and $c_2 = \epsilon_y - \epsilon_{yx}$, and define a directionality index D in such a way that:

$$D^{(l)} = \frac{c_2 - c_1}{c_2 + c_1} = \frac{\epsilon_y - \epsilon_x + \epsilon_{xy} - \epsilon_{yx}}{\epsilon_y + \epsilon_x - \epsilon_{xy} - \epsilon_{yx}} \tag{3.20}$$

in which the superscript l indicates the linearity of the calculation procedure for the indicator.

The meaning of $D^{(l)}$ is obvious: ranging from 1 ($x \to y$ flow) to -1 (influence of type $y \to x$), the index measures how much a signal x causes another signal y according to Granger, also providing an indication on the direction of causality. The intermediate values may indicate either a smaller causal connection between the two time series or a bi-directionality of the same, in such a way that the two flows (different in strength and of opposite sign) will compensate and generate intermediate values. The limiting case is the one in which the measured value is zero: in this case it cannot be said with certainty that there is no causal connection between the two series, but simply that there is not a net flow of causality between the source and the destination.

This imprecision is exceeded, as we shall see in the following paragraphs, reconsidering functional form of $D^{(l)}$, cue even for important considerations.

For sufficiently long time series (ie, for N sufficiently large, as for example the EEGs we are going to deal with, spanning from a few thousand to about 10^5 samples), and according to the definition of causality, the following two properties hold:

- *if* $\mathbf{Y}$ *is not correlated with* $\mathbf{X}$ *and* x*, then* $\epsilon_x = \epsilon_{xy}$;
- *if* $\mathbf{X}$ *is not correlated with* $\mathbf{Y}$ *and* y*, then* $\epsilon_y = \epsilon_{yx}$.

Considering only the first of the two properties (for the second the symmetrical speech holds), the non-correlation results mathematically in the fact that the operator $\tilde{A}^{-1}$ is diagonal ($\Sigma_{xy} = \Sigma_{yx} = 0$) and at the same time the vector $\mathbf{T_{12}}$, that somehow blend causal dependencies, is identically zero. As a result, VAR and AR models coincide.

According to the definition, the possibility that the indicator $D^{(l)}$ proves himself suitable to describe a linear type causality lies in the verifying of above mentioned properties. However, it may be necessary to overcome the assumptions of linearity and consider possible *nonlinear* causal links between

two temporally ordered series, as in these conditions the higher order corrections may become more important than in the linear case, in which are confined in bidding for small corrections.

In this case the two previously considered properties must be restated [31] in the following way:

- *if* $\mathbf{Y}$ *is statistically independent of* $\mathbf{X}$ *and* x*, then* $\epsilon_x = \epsilon_{xy}$;
- *if* $\mathbf{X}$ *is statistically independent of* $\mathbf{Y}$ *and* y*, then* $\epsilon_y = \epsilon_{yx}$.

As recent studies [32] have tried to bring the non-linear causality in the filed of the linear one (as occurs with the local approximation of a curve with a straight line segment in Euclidean spaces), the best practicable choice is to consider sets of functions that, characterizing the non-linearity of causality, can *globally* fullfill the property just enunciated.

3.3 Granger Causality: nonlinear model

To characterize the non-linearity in the Granger causality we must reconsider the initial system (3.1) introducing two generic non-linear vector functions, each of n components and m variables, $\Psi = (\psi_1, ..., \psi_n)$ and $\Phi = (\phi_1, ..., \phi_n)$ [30]:

$$\begin{cases} x = \mathbf{\Omega_{11}} \cdot \mathbf{\Psi}(\mathbf{X}) + \mathbf{\Omega_{12}} \cdot \mathbf{\Phi}(\mathbf{Y}) \\ y = \mathbf{\Omega_{21}} \cdot \mathbf{\Psi}(\mathbf{X}) + \mathbf{\Omega_{22}} \cdot \mathbf{\Phi}(\mathbf{Y}) \end{cases} \tag{3.21}$$

where $\{\mathbf{\Omega}\}$ is a set of 4 real n-dimensional vectors. The choice of the integer n is related, as we shall see, to the choice of the basic nonlinear functions. Once the two functions Ψ and Φ are fixed, the system (3.21) represents a linear space generated by the components of the two functions in $4n$ variables $\{\mathbf{\Omega}\}$, whose elements have to be established to minimize the prediction errors.

Using, as before, the least squares method to solve the system (3.21) we obtain:

$$\begin{pmatrix} \mathbf{\Omega_{11}} \\ \mathbf{\Omega_{12}} \end{pmatrix} = \begin{pmatrix} \mathbf{S_1} & \mathbf{S_2} \end{pmatrix}^{\dagger} \mathbf{t_1} \tag{3.22}$$

and

$$\begin{pmatrix} \mathbf{\Omega_{21}} \\ \mathbf{\Omega_{22}} \end{pmatrix} = \begin{pmatrix} \mathbf{S_2} & \mathbf{S_1} \end{pmatrix}^{\dagger} \mathbf{t_2} \tag{3.23}$$

where † refers to the pseudo-inverse matrix [33] and elements of $\mathbf{S}$ and $\mathbf{t}$ are computed as follows:

$$\begin{aligned} &[\mathbf{S_1}]_{k\rho} = \psi_\rho(\mathbf{X}^k) \quad \textit{with} \quad k = 1, ..., M; \rho = 1, ..., n \\ &[\mathbf{S_2}]_{k\rho} = \phi_\rho(\mathbf{Y}^k) \quad \textit{with} \quad k = 1, ..., M; \rho = 1, ..., n \\ &[\mathbf{t_1}]_k = x^k \quad \textit{with} \quad k = 1, ..., M \\ &[\mathbf{t_2}]_k = y^k \quad \textit{with} \quad k = 1, ..., M \end{aligned} \tag{3.24}$$

Based on the knowledge of these elements, we obtain

$$\epsilon_{yx} = \frac{1}{M} \sum_{k=1}^{M} \left[y^k - \mathbf{\Omega_{21}} \cdot \mathbf{\Psi}(\mathbf{X})^k - \mathbf{\Omega_{22}} \cdot \mathbf{\Phi}(\mathbf{Y})^k \right]^2 \tag{3.25}$$

$$\epsilon_{xy} = \frac{1}{M} \sum_{k=1}^{M} \left[x^k - \mathbf{\Omega_{11}} \cdot \mathbf{\Psi}(\mathbf{X})^k - \mathbf{\Omega_{12}} \cdot \mathbf{\Phi}(\mathbf{Y})^k \right]^2 \tag{3.26}$$

Such non-linear VAR model is to be compared, as previously, with the non-linear AR model, which is based on system

$$\begin{cases} x = \mathbf{\Gamma_1} \cdot \mathbf{\Psi}(\mathbf{X}) \\ y = \mathbf{\Gamma_2} \cdot \mathbf{\Phi}(\mathbf{Y}) \end{cases} \tag{3.27}$$

and consequently

$$\mathbf{\Gamma_1} = \mathbf{S_1^\dagger t_1} \quad , \quad \mathbf{\Gamma_2} = \mathbf{S_2^\dagger t_2} \tag{3.28}$$

$$\epsilon_x = \frac{1}{M} \sum_{k=1}^{M} \left[x^k - \mathbf{\Omega_{11}} \cdot \mathbf{\Psi}(\mathbf{X})^k \right]^2 \tag{3.29}$$

$$\epsilon_y = \frac{1}{M} \sum_{k=1}^{M} \left[y^k - \mathbf{\Omega_{22}} \cdot \mathbf{\Phi}(\mathbf{Y})^k \right]^2 \tag{3.30}$$

Even in this non-linear model, the $D^{(nl)}$ parameter has the same functional structure as the linear case, with the only difference that the determining elements has a nonlinear behavior.

It is possible to prove [30] that this choice satisfies the conditions seen previously: if we impose the conditions are true (eg, the first), then for every $\mu = 1, ..., n$ and for every $\nu = 1, ..., n$, the function $\phi_\mu(Y)$ results as not correlated with x and $\Psi_\nu(X)$. It follows that

$$\begin{aligned} &Var\left[x - \mathbf{\Omega_{11}} \cdot \mathbf{\Psi}(\mathbf{X}) - \mathbf{\Omega_{12}} \cdot \mathbf{\Phi}(\mathbf{Y})\right] \\ &= Var\left[x - \mathbf{\Omega_{11}} \cdot \mathbf{\Psi}(\mathbf{X})\right] + Var\left[\mathbf{\Omega_{12}} \cdot \mathbf{\Phi}(\mathbf{Y})\right] \end{aligned} \tag{3.31}$$

and for very large N, ϵ_{xy} tends to zero in accordance with its definition, Ω_{12} will be identically null and the non-linear AR and VAR coincide. The same procedure is true swapping x and y .

What still remains unsettled is the choice of non-linear functions to be used. The indication is to use classical p-degree polynomial functions (returning, for $p = 0$, the linear case) or Gaussian functions. From the computational speed point of view of there is not much difference between the two; however, the Gaussian function is preferable, both because best approximates the probability distribution of the experimental points (as has already been seen for the TE), and because is well suited for use in clustering algorithms as kernel [34, 35, 36].

In this case, setting $n << M$ in the space spanned by the vector $\mathbf{X}$, n different centers of coordinate $\{\tilde{\mathbf{X}}^\rho\}_\rho^M = 1$ are primarily identified (with any

clustering method applied to the data $\{\mathbf{X}^k\}_{k=1}^M$). The same occuring in the space of the vectors $\mathbf{Y}$, finding the n centers $\{\tilde{\mathbf{Y}}^\rho\}_{\rho=1}^M$. Such centers represent the *prototypes* of the variables $\mathbf{X}$, for which the functions ψ represent a measure of the similarity between the paths from these to every other point in the space. The same applies to $\{\tilde{\mathbf{Y}}^\rho\}$ and ϕ .

From a mathematical point of view, for the p-degree polynomials, the choice of the functions to be used is

$$\begin{aligned} \psi_\rho(\mathbf{X}) &= \left(1 + \mathbf{X} \cdot \tilde{\mathbf{X}}^\rho\right)^p, \quad \textit{with } \rho = 1, ..., n \\ \phi_\rho(\mathbf{Y}) &= \left(1 + \mathbf{Y} \cdot \tilde{\mathbf{Y}}^\rho\right)^p, \quad \textit{with } \rho = 1, ..., n \end{aligned} \tag{3.32}$$

while for the Gaussian functions we have:

$$\begin{aligned} \psi_\rho(\mathbf{X}) &= \exp\left(-\frac{||\mathbf{X} - \tilde{\mathbf{X}}^\rho||^2}{2\sigma^2}\right), \quad \textit{with } \rho = 1, ..., n \\ \phi_\rho(\mathbf{Y}) &= \exp\left(-\frac{||\mathbf{Y} - \tilde{\mathbf{Y}}^\rho||^2}{2\sigma^2}\right), \quad \textit{with } \rho = 1, ..., n \end{aligned} \tag{3.33}$$

in which σ is a parameter that must be fixed in each case in such a way as to avoid the data overfitting, but whose order of magnitude, in any case, is that of the average distance between the points of the spaces generated by $\mathbf{X}$ and $\mathbf{Y}$. The advantage of using Gaussian functions with respect to the polynomials lies in the fact that the first describes all degrees of non-linearity of the distribution of the experimental points.

Finally, note how the (3.32) and (3.33) assume the kernel used to be linear both in $\mathbf{X}$ and $\mathbf{Y}$, not there being an explicit relationship mixing the variables $\mathbf{X}$ and $\mathbf{Y}$. This choice is, at moment, the only one compatible with the conditions of statistical independence between $\mathbf{X}$ and $\mathbf{Y}$ imposed at the end of the previous paragraph.

3.3.1 Complete Multivariate Model (MVAR)

As we have seen in the last paragraph, the GC has a vagueness in the definition of the indicator D, not defining precisely *how much* a time series is G-causing a second if not for the *net* influence that the second can have on the first. To overcome this ambiguity, we can choose [37] not to use the given definition, but to directly compare the variances of their accuracy errors in the VAR and AR cases: in this way the systems (3.1) and (3.15) can be corrected by introducing the precision errors $\epsilon_{(.)}$ and generalizing to the case of *all* the future of every single series $\mathbf{X}$ and $\mathbf{Y}$ in the following way [38]:

$$\begin{cases} x(t) = \sum_{j=1}^{m} \mathbf{W_{11,j}} \cdot \mathbf{X}(t-j) + \sum_{j=1}^{m} \mathbf{W_{12,j}} \cdot \mathbf{Y}(t-j) + \epsilon_{xy}(t) \\ \\ y(t) = \sum_{j=1}^{m} \mathbf{W_{21,j}} \cdot \mathbf{X}(t-j) + \sum_{j=1}^{m} \mathbf{W_{22,j}} \cdot \mathbf{Y}(t-j) + \epsilon_{yx}(t) \end{cases} \tag{3.34}$$

and

$$\begin{cases} x(t) = \sum_{j=1}^{m} \mathbf{V_{1,j}} \cdot \mathbf{X}(t-j) + \epsilon_{x}(t) \\ y(t) = \sum_{j=1}^{m} \mathbf{V_{2,j}} \cdot \mathbf{Y}(t-j) + \epsilon_{y}(t) \end{cases} \tag{3.35}$$

where m is as always the model *order* (ie, number of elements of the past that may affect the future of destination series) and the vectors $x(t)$ and $y(t)$ represent all elements of the future (what you want extended in time, let's just say, T) of the set $\mathbf{X}$ and $\mathbf{Y}$.

As seen above, if the error ϵ_{xy} is smaller than ϵ_x in module, then the set $\mathbf{Y}$ has a causal influence on $\mathbf{X}$ ("$\mathbf{Y}$ G-causes $\mathbf{X}$"). Obviously we can not use the difference between the two errors to define the indicator $D^{(l)}$ (or $D^{(nl)}$), otherwise we would commit the same above error: we will use, rather, the ratio of their variance in the following way

$$D^{(l)}_{x \to y} = \ln\left(\frac{Var(\epsilon_x(t))}{Var(\epsilon_{xy}(t))}\right) \tag{3.36}$$

which is, of course, time-independent (in fact it is calculated on the entire *future* of the time series). The extension to the nonlinear case trivially occurs

with the same procedures as previously seen. We will return on the value of the m parameter, the order of the model.

What makes crucial the calculation of G-causality is not, however, the model used (linear or not, of any order), but the fact that this, as presented so far, does not yet take into account the *contemporary influence that each other time series may have on the series under consideration* (in the case of electroencephalograms we are going to deal with, there are about 60 time series influencing each other). In any case, the mathematical structure of GC is well suited for multivariate extension (MVAR), and to prove it [37] let's consider as an example the case of three time series $\mathbf{X}$, $\mathbf{Y}$ and $\mathbf{Z}$ influencing each other (higher dimensional cases follow immediately from this).

If we insert the three series in the VAR model we obtain:

$$\begin{cases} x(t) = \mathbf{W_{11,j}} \cdot \mathbf{X}(t-j) + \mathbf{W_{12,j}} \cdot \mathbf{Y}(t-j) + \mathbf{W_{13,j}} \cdot \mathbf{Z}(t-j) + \epsilon_{xyz}(t) \\ \\ y(t) = \mathbf{W_{21,j}} \cdot \mathbf{X}(t-j) + \mathbf{W_{22,j}} \cdot \mathbf{Y}(t-j) + \mathbf{W_{23,j}} \cdot \mathbf{Z}(t-j) + \epsilon_{yxz}(t) \\ \\ z(t) = \mathbf{W_{31,j}} \cdot \mathbf{X}(t-j) + \mathbf{W_{32,j}} \cdot \mathbf{Y}(t-j) + \mathbf{W_{33,j}} \cdot \mathbf{Z}(t-j) + \epsilon_{zxy}(t) \end{cases} \tag{3.37}$$

where the sum over the index j from 1 to the order m of the model is upon-intended. Now the errors $\epsilon_{(.)}(t)$ consider both the case of the simultaneous presence of the three series and the case in which, case by case, one of the three series is not taken into account. In the same way, the corresponding MAR model must consider all possible combinations.

A simple way to proceed is to consider separately the covariance matrix of the complete model:

$$\Sigma = \begin{bmatrix} Var(\epsilon_{xyz}) & Cov(\epsilon_{xyz}, \epsilon_{yxz}) & Cov(\epsilon_{xyz}, \epsilon_{zxy}) \\ Cov(\epsilon_{yxz}, \epsilon_{xyz}) & Var(\epsilon_{yxz}) & Cov(\epsilon_{yxz}, \epsilon_{zxy}) \\ Cov(\epsilon_{zxy}, \epsilon_{xyz}) & Cov(\epsilon_{zxy}, \epsilon_{yxz}) & Var(\epsilon_{zxy}) \end{bmatrix} \tag{3.38}$$

and the $n - 1$ covariance matrices of the *restricted* models, or better the models in which, case by case, a time series is omitted:

$$\rho_x = \begin{bmatrix} Var(\epsilon_{xy}) & Cov(\epsilon_{xy}, \epsilon_{yx}) \\ Cov(\epsilon_{xy}, \epsilon_{yx}) & Var(\epsilon_{yx}) \end{bmatrix}$$
$$\rho_y = \begin{bmatrix} Var(\epsilon_{xz}) & Cov(\epsilon_{xz}, \epsilon_{zx}) \\ Cov(\epsilon_{zx}, \epsilon_{xz}) & Var(\epsilon_{zx}) \end{bmatrix} \quad (3.39)$$
$$\rho_z = \begin{bmatrix} Var(\epsilon_{zy}) & Cov(\epsilon_{zy}, \epsilon_{yz}) \\ Cov(\epsilon_{zy}, \epsilon_{yz}) & Var(\epsilon_{yz}) \end{bmatrix}$$

The meaning of the matrices elements we have just seen is quite straight: for example, if we consider only the elements ϵ_{xyz} and ϵ_{xz} we have:

$$\begin{aligned} \epsilon_{xyz} &= Var(x - \mathbf{W_{11}X} - \mathbf{W_{12}Y} - \mathbf{W_{13}Z}) \\ \epsilon_{xz} &= Var(x - \mathbf{W_{11}X} - \mathbf{W_{13}Z}) \end{aligned} \quad (3.40)$$

and so on for all the others.

In this model, certainly more realistic of the previous, the amount (measured in bits) with which the series **X** G-causes the set **Y** *in the presence of the set* **Z** (affecting **Y** too) is

$$D^{(l)}_{x \to y|z} = \ln\left(\frac{(\rho_x)_{11}}{\Sigma_{11}}\right) \quad (3.41)$$

In this way, the uncertainty due to the difference of the two information flows inherent in the first definition of D is exceeded, the contemporary presence of a second time series that may affect the first is considered and, for each pair of the n time series appearing in the model, a not necessarily symmetric $n \times n$ connection matrix is achieved.

An important parameter to be fixed is the order m of the model [39]: if from a purely theoretical point of view, m should be as high as possible (for $m \to \infty$ there would be the cancellation of the uncertainties and their

variances), from a computational point of view we must seek a compromise between computational speed and accuracy of the model. Generally, the estimation of the model order is made on the basis of two hypotheses, which in a different way minimizes the ratio between the variance of the model and the number of coefficients to be calculated (basing on the n time series we chose to consider). A first choice is from Akaike (Akaike Information Criterion, [40]), which calculates this integer as the minimum of the following function

$$AIC(m) = \ln\left[det(\Sigma)\right] + \frac{2mn^2}{T} \tag{3.42}$$

while the second, the Bayesian Information Criterion (BIC, [41]) assumes that the function to be minimized is

$$BIC(m) = \ln\left[det(\Sigma)\right] + \frac{\ln(T^2) \cdot mn^2}{T} \tag{3.43}$$

The BIC model is certainly the most used in neuroscience, as more agile when we have to manage particularly extended data series. However in cases where the BIC (or AIC) provides or an order m too high for the calculation to be computationally efficient, or even in the case in which there is not a defined minimum (presence of more than one only minimum or even *plateaux*), then the parameter m can be chosen smaller [37], provided the models BIC/AIC substantially do not present further decrease beyond that limit [39].

3.4 Equivalence between Transfer Entropy and Granger Causality

Let us return to the formulation of the multivariate GC and show that, in the calculation of the lowest linear order of GC and TE, the two quantities are equivalent as long as the original time series exhibit a Gaussian distribution.

In a slightly different formalism from the one we have used till now but absolutely equivalent due to Geweke [37] and already used for the multivari-

ate redefinition of TE (cite paragraph 1.6), if we denote by $\mathbf{\Sigma}(\epsilon)$ the error variance of $\epsilon_{(\cdot)}$, then (3.41) can be rewritten as follows:

$$D^{(l)}_{x\to y|z} = \ln\left(\frac{\mathbf{\Sigma}(\epsilon_{xz})}{\mathbf{\Sigma}(\epsilon_{xyz})}\right) = \ln\left(\frac{\mathbf{\Sigma}(x|X^- \oplus Z^-)}{\mathbf{\Sigma}(x|X^- \oplus Y^- \oplus Z^-)}\right) \tag{3.44}$$

Let's assume now that the time series on which TE is calculated are characterized by a *Gaussian* distribution, for which we can rely on the relationship between the entropy of the time series (let's call it $\mathbf{X}$) and its variance [42]:

$$H(\mathbf{X}) = \frac{1}{2}\left\{\ln\left(|\mathbf{\Sigma}(\mathbf{X})|\right) + n\ln\left(2\pi e\right)\right\} \tag{3.45}$$

where n is the dimension of $\mathbf{X}$. For the conditional entropy there is a very similar relationship. We start, in fact, from considering the well known property [42]:

$$\begin{aligned} H(\mathbf{X}|\mathbf{Y}) =& H(\mathbf{X}\oplus\mathbf{Y}) - H(\mathbf{Y}) = \\ =& \frac{1}{2}\ln\left(|\mathbf{\Sigma}(\mathbf{X}\oplus\mathbf{Y})|\right) - \frac{1}{2}\ln\left(|\mathbf{\Sigma}(\mathbf{Y})|\right) + \frac{1}{2}n\ln\left(2\pi e\right) \end{aligned} \tag{3.46}$$

and remember that, at the same time:

$$\mathbf{\Sigma}(\mathbf{X}\oplus\mathbf{Y}) = \begin{pmatrix} \mathbf{\Sigma}(\mathbf{X}) & \mathbf{\Sigma}(\mathbf{X},\mathbf{Y}) \\ \mathbf{\Sigma}(\mathbf{X},\mathbf{Y})^\dagger & \mathbf{\Sigma}(\mathbf{Y}) \end{pmatrix} \tag{3.47}$$

By inserting at this point the known matix identity [43]

$$\begin{vmatrix} A & B \\ C & D \end{vmatrix} = |D|\cdot|A - BD^{-1}C| \tag{3.48}$$

we easily obtain

$$\mathbf{\Sigma}(\mathbf{X}\oplus\mathbf{Y}) = |\mathbf{\Sigma}(\mathbf{X})|\cdot|\mathbf{\Sigma}(\mathbf{X}|\mathbf{Y})| \tag{3.49}$$

ie the profit result:

$$H(\mathbf{X}|\mathbf{Y}) = \frac{1}{2}\left\{\ln\left(|\mathbf{\Sigma}(\mathbf{X}|\mathbf{Y})|\right) + n\ln\left(2\pi e\right)\right\} \tag{3.50}$$

that, extended to the case of three variables (with a recursive procedure) and substituted into (1.33, Chapter 1), returns:

$$T_{y\to x|z} = \frac{1}{2}\ln\left(\frac{\mathbf{\Sigma}(x|X^{-}\oplus Z^{-})}{\mathbf{\Sigma}(x|X^{-}\oplus Y^{-}\oplus Z^{-})}\right) = \frac{1}{2}D^{(l)}_{x\to y|z} \tag{3.51}$$

therefore *the Granger causality coincides with the Transfer Entropy less than a factor of 2 if the parental time series shows a Gaussian distribution.* The property still keeps true even when the number of the considered time series is greater than the three used in this demonstration.

Chapter 4

Brain Networking

4.1 Introduction

The analysis presented so far, and in particular those relating to the connectivity between different brain areas, only fournishes a partial view of what happens in the transmission of information between cortical areas: the TE, like the GC, provides simply a "map" of functional activation that can be studied in greater detail with more sophisticated tools as network analysis, the so-called *Networking* [44].

Born in computing science as an evolution of the classic *graph theory*, this theory deals with the description, both global and local, of the connection between the sub-components of a complex system, as one can consider the brain (in our case, the cortex only, with related areas) [44]. A number of indicators, in fact, verify if the flow of information passes unhindered through these components, if is facilitated by some areas and inhibited by others, if the network is organized in sub-structures that communicate between them or that tend to isolate themselves and so on. We can say that if the connectivity analysis is engaged in *intensity* of connections, the networking studies the internal *dynamic* of connections and the relationship between its components.

The application of this theory to the cortex functional connectivity is

called *Brain Networking* (BN), and constitutes the frontier of research in computational neuroinscience.

4.2 Networking Principles

A network is the mathematical representation of a real system in what you want complex, defined by a set of *nodes* (or *vertices*) and connections between them (*links*, or *edges*).

Basically there are three different types of networks that can be studied: anatomical, functional and effective. The first studies the structure of physiological connection between brain areas, highlighting traits of organic matter that physically connect them. The other two, instead, study respectively the correlation and the causal relationship between the electrical activity of various brain areas: one can say that while the first is a "physical" network for the brain, the other two represent the network of causality and information flowing through it.

In BN, nodes represent specific areas of the cortex: in particoular the scalp areas covered by individual electrodes that can be later reorganized into the more "classical" frontal, central, temporal, parietal and occipital lobes, left and right in the variants [45]. What is particularly relevant is that the mounting pattern of the electrodes must cover the whole scalp as much as possible without overlapping, in order to avoid effects of masking of the actual measures or create "ghost" areas that would appear, as will be clear later, particularly segregated [46, 47]. It 'also important to emphasize that the assembly diagram and covering of the scalp (*parcellation*) should be uniquely determined and never changed during the analysis, as the results differ according to the same scheme [48, 49].

This does not mean, however, that the results change with the order in which the channels are followed in the analysis: it can be shown, in fact, that the results of the analysis are invariant under permutation of the channels

within the same parcellation scheme. As part of this work we have used the internationally recognized *extended 10-20* scheme, or 61 electrodes uniformly distributed over the scalp.

Similarly, the nature of the link and the interpretation of their intensity varies depending on the connection matrix used: for anatomical connection, the link strength is an estimate of the amount of white matter that acts like a "bridge" between different brain areas, while for the functional and effective networks, the link represents the intensity of the connection or the correlation, both in linear and non-linear type [50, 51].

From the computational point of view, the description a network requires an activation map whose individual elements represent the strength of the connection between the nodes i and j. For the functional and effective networks, the connection matrix belongs from the connectivity analysis performed with the tools seen in the previous chapters: the SE and the CTCC are used for functional connectivity being symmetric matrices (or "undirected"), while the TE and the GC are preferred for the study of effective connectivity ("directed" matrices). Anyway, matrices from TE and GC can be symmetrized (for each pair $\{i, j\}$ the largest matrix element in the module is chosen), in order to be used as an entry for the functional network.

Another exception should be made on the form of the connection matrix elements. In fact, it is possible to work with binary or weighed matrices: in the first case (after applying a suitable threshold, see below), the elements have logic 1 or 0 value (presence or absence of connection), while in the second case the value is the one calculated with the appropriate indicators, highlighting an intensity value of the connection too.

In the present study, the anatomical connectivity will not be considered, since -at the moment- the results cannot be compared with those of the other two types of network: these, in fact, start from the assumption that the information transmission takes place at cortico-cortical level, while the

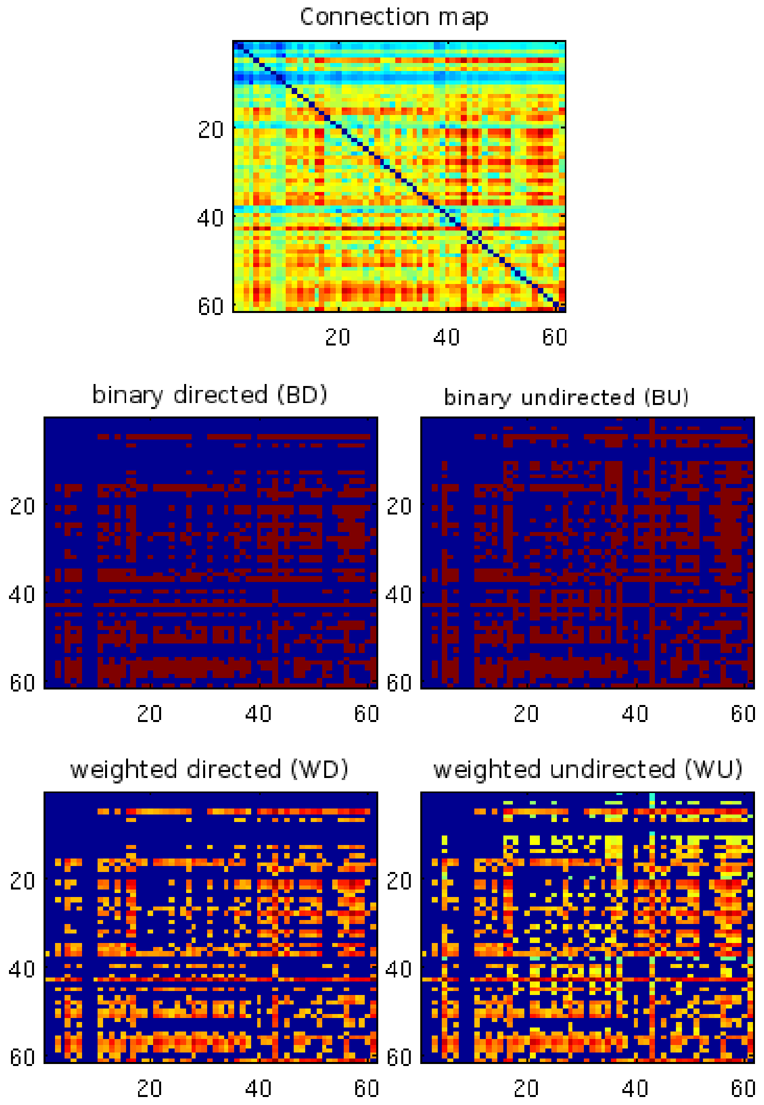

Figure 4.1: Examples of thresholding and readjustments of the activation map: the TE starting map (top); at center, the same map undergoing thresholding of 60 % has been firstly binarized (BD) and then symmetrized (BU); bottom: after the threshold application, the map has been symmetrized (WU), or simply left unchanged (WD).

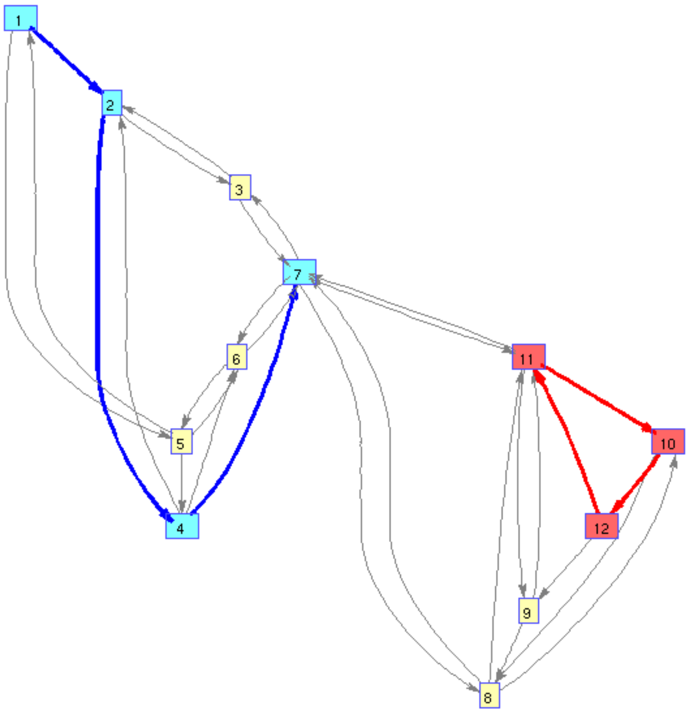

Figure 4.2: example of a graph associated with a directional network consisting in 12 nodes and 40 links. The bimodular structure of the network is evident as well as the strong centrality of node 7, which acts as an intermediary between the two strongly segregated sub-network, $1 \leftrightarrow 6$ and $8 \leftrightarrow 12$. Also, notice all the *motifs* (such as the blue line pattern) and the *triangles* (the red circuit, which can not be path reversally) present in the network.

anatomical connectivity (deduced, for example, by an MRI tractography) generates a network whose connections are cortico-subcortical in addiction. For sure, the next step in the description of the brain real network will be the interconnection and simultaneous interpretation of the anatomical and informational network, but will not be the subject of this work.

We will therefore focus, as we shall see in more detail, on the functional and effective connectivity, favoring whenever possible the latter, as the aspect of *direction* of the information flow through the links is of paramount

importance in this study: an indirect matrix (symmetric or symmetrized) in fact does not show differences in flow directionality, since nodes i and j are linked with the same intensity, while in the case of directional matrices it is possible to distinguish differences in connectivity from i to j and vice versa; in this case we can reconstruct the actual path followed by the information which may, in principle, be not perfectly symmetric in the path back and forth between two nodes.

We can not choose *a priori*, however, which version of the connection matrix deal with (binary or weighted, directed or indirect), as each single quantity described later or relies on a particular version (the *distance*, for example, measures the distance between two nodes counting the number of intermediate vertices: the binary version of the connection matrix is sufficient), or presents two versions of itself, weighted and binary (it is the case, for example, of the couple *degree* - *strength*). For this reason, hereinafter, using as much as possible the weighted versions of the activation matrices is preferred, binarizing them only if strickly necessary or when requested by the definitions. The same considerations apply to the direct and indirect (symmetrized) versions of the same matrix: the first will always be preferred to the latter. In other words, effective connectivity will always be preferred to functional one.

4.3 The threshold problem

Before we can use them for analysis, any element whose value is particularly small must be properly removed from the connection matrices, as they represent spurious or little significance connections that can obscure the topology of strongest and most significant connections [52]. It is therefore necessary to apply a threshold to eliminate those items.

The choice that can be made is twofold: the threshold can be *absolute* or *relative* (or *adaptive*): in the first case an absolute minimum value is chosen,

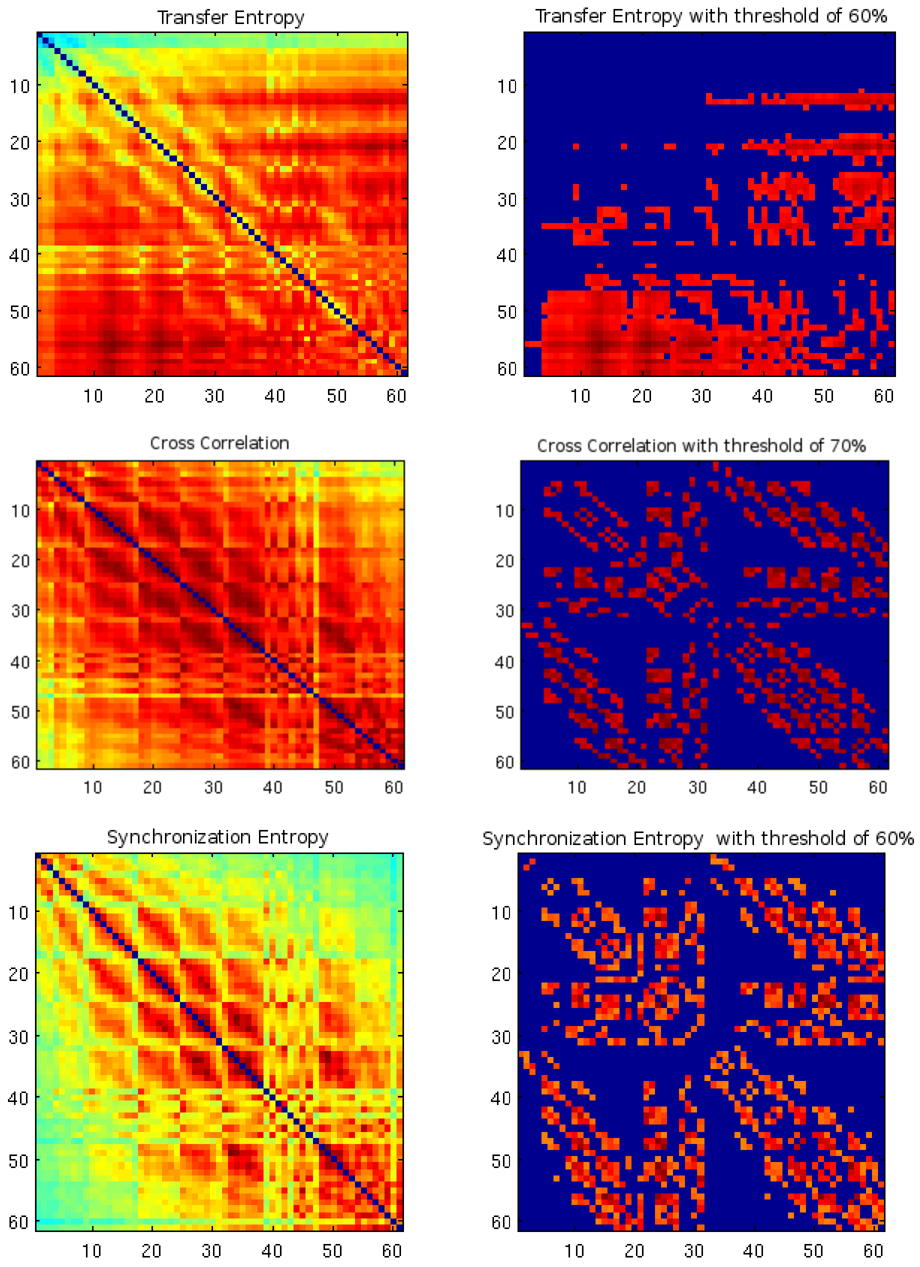

Figure 4.3: examples of network subjected to adaptive thresholding. From top to bottom we find TE, CC and SE undergoing different threshold values. All versions are left weighted and directed (WD).

independently of the model used or the applied stimulation, under which threshold each array element is reset. In the second case a value between 0 and 1 is chosen which cuts, for each activation matrix, each element whose module falls below the product between this value and the largest matrix element in the matrix: in practice, every element whose module is below a certain percentage of the maximum value of the entire array is set to zero. This second method is more efficient than the first, since it allows to avoid inconsistent results as identically null matrices (that is, a "totally disconnected network") and allows the study of the network basing not on the intensity of the connection but according to the relative dynamics between its parts [53].

Likewise, the wisest choice would be the one that does not uniquely fix the threshold and preserves it throughout the analysis, but does consider the different behavior of the network throughout the entire threshold range, so as one can analyze the variability of the quantities as a function of the information flow level (although if integrated) flowing through the network.

It is also true that, as we shall see in the data analysis section, within a broad ranges of threshold value, the network does not present a great variability, for which the choice of the threshold could fall into one of these intervals in which the indicators have not a monotonically increasing or decreasing trend but presents a plateau both in absolute value and in variance. Usually these intervals are the central ones, ranging between 0.35 and 0.75.

4.4 Caracteristic features of BN

A single measurement made on the network is able to characterize various aspects of brain connectivity, both at local and global level. Throughout this section, we present several measures that, in different ways, describe different aspects of functional integration and segregation, which quantify the importance of the individual cortex areas, characterize the information routes and the different possible circuits that can be followed by information,

analyzing the potentialities of a network to restore the connections that may have interrupted due to any cause (pathological, physical, etc..).

4.4.1 Basic Measures

Each measurement made on BN is based on the definition of some fundamental quantities, which serve, in various ways, to characterize the network right from the start and to better interpret the subsequent and more sophisticated measures, better placing them in the general context.

Each single measure should not be considered in its singularity, but as part of a distribution that best characterizes the network in one hand and data ensambles on the other.

Let then N the number of all possible sets of nodes in a network and n their total number. Let L the number of all possible link sets and l their total number. (i, j) represents a link between the nodes i and j, with $i, j \in N$.

Let $a_{i,j}$ the connection status between i and j: if $a_{i,j} = 0$, then the two nodes are disconnected, otherwise they are defined *close* ($a_{i,j} = 1$ in the binary network, $a_{i,j} = w_{i,j}$ if the network is weighted: from now on, the weights will be considered normalized, so that $0 \leq w_{i,j} \leq 1$).

For a binary network, the number of links is defined as

$$l = \sum_{i,j \in N} a_{ij} \tag{4.1}$$

while for the weighed ones it has the form

$${}^{w}l = \sum_{i,j \in N} w_{ij} \tag{4.2}$$

Note that for undirected networks, $a_{i,j} = a_{j,i}$, therefore in (4.1) and (4.2) each link is counted twice, while for directed networks each link is counted once and only once, each of them being directed from node i to the j in a

unique way.

The first measure that can be made on the network is the *degree* of each single network node, considered as the number of connections (total, ingoing and outgoing) departing from each single node:

$$k_i = \sum_{j \in N} a_{ij} \tag{4.3}$$

For the direct version we can distinguish the degree in two ways, for the incoming and outgoing traffic:

$$k_i^{in} = \sum_{j \in N} a_{ji} \qquad , \qquad k_i^{out} = \sum_{j \in N} a_{ij} \tag{4.4}$$

In this context, the degree is a measure of how many nodes are close to the actual node (*neighbor node*). If the network is undirected, the number of ingoing connections equals the outgoing ones. In any case, the total degree is the sum of two degrees in input and output. Moreover, the degree is a measure of the importance that a node (or a cluster of nodes) plays within a network.

The weighted version of the node degree, called *strength*, sums the intensities of each individual connection of a node instead of the individual nodes number:

$$s_i = {}^{w}k_i = \sum_{j \in N} w_{ij} \tag{4.5}$$

The set of degrees of all nodes in the network defines the *degree distribution*, an important indicator of the ability of the network to regenerate broken connections, actually dropped due to internal or external causes.

The average degree of the entire network is known as *density*, and is the *wiring cost* of the network: the higher this indicator, the more efficient the network.

We can try to measure the *shortest path length* between two single nodes i and j by firstly expressing with $g_{i\leftrightarrow j}$ the smaller path in length between node i and the j (ultimately, the geodesic between two nodes) and then summing over all the links belonging to them. The weighted version also requires the calculation of the map (ie, the inverse matrix) from weights to lengths, $f(w_{i,j})$:

$$d_{ij} = \sum_{a_{uv} \in g_{i\leftrightarrow j}} a_{uv} \qquad , \qquad {}^{w}d_{ij} = \sum_{a_{uv} \in {}^{w}g_{i\leftrightarrow j}} f(w_{uv}) \tag{4.6}$$

The directed version, as for the degree, distinguishes the distance from i to j from the inverse, replacing the generic geodesic $g_{i\leftrightarrow j}$ with the *direct* one $g_{i\rightarrow j}$:

$$d_{ij}^{\rightarrow} = \sum_{a_{uv} \in g_{i\rightarrow j}} a_{uv} \qquad , \qquad {}^{w}d_{ij}^{\rightarrow} = \sum_{a_{uv} \in {}^{w}g_{i\rightarrow j}} f(w_{uv}) \tag{4.7}$$

It is important to emphasize how the characteristics of a network are strongly influenced by these initial measures, as well as by the number of nodes and links. For this reason, wheter we have not at least two populations to compare among them or we want to test the null hypothesis that our results are only artifacts or due to casual distribution of nodes within the network, it is possible to artificially generate comparison networks for each measure (the so-called *null model*, NM) that has to share all these quantities with the real network under investigation. On the other hand, the topology of the NM and its node's spatial distribution plays no role: different topologies are allowed, ranging from random to fractal [54].

4.4.2 Functional Segregation measures

Functional segregation refers to the network tendency to let certain processes take place only within particoluar areas that, most of times, are inner densely connected and present, at the same time, a quite reduced number of connections from and to the outside compared to the internal ones.

In the first place, the segregation measures quantify the number of such areas, called *modules* (or *clusters*), the presence of which indicates the possi-

bility that the related areas of the network are segregated. Alternatively, it can be stated that, in functional and effective networks, the abnormal presence of clusters is an indicator of a possible strongly structured hierarchy in the information path.

The simplest measures of segregation are based on the counting of *triangles* (or *polygons*, closed circuits in the patterns followed by the information, consisting of n nodes and the same number of link) in the network, whose relative abundance is an important factor in the study of segregation.

We will define the *triangles* around the node i in the binary network by means of the following relation:

$$t_i = \frac{1}{2} \sum_{j,h \in N} a_{ij}\, a_{ih}\, a_{jh} \tag{4.8}$$

while for weighed and directed ones the following definitions apply:

$$^{w}t_i = \frac{1}{2} \sum_{j,h \in N} (w_{ij} w_{ih} w_{jh})^{1/3} \tag{4.9}$$

$$t_i^{\rightarrow} = \frac{1}{2} \sum_{j,h \in N} (a_{ij} + a_{ji})(a_{ih} + a_{hi})(a_{jh} + a_{hj}) \tag{4.10}$$

For each single node, the fraction of triangles surrounding each vertex is defined as the *clustering coefficient*

$$C_i = \frac{2t_i}{k_i(k_i - 1)} \tag{4.11}$$

therefore the entire network clustering coefficient can be calculated as follows:

$$C = \frac{1}{n} \sum_{i \in N} C_i \tag{4.12}$$

with the usual weighted and directed variants:

$$^{w}C = \frac{1}{n} \sum_{i \in N} \frac{2 \cdot {}^{w}t_i}{k_i(k_i - 1)} \tag{4.13}$$

$$C^{\rightarrow} = \frac{1}{n} \sum_{i \in N} \frac{2t_i^{\rightarrow}}{(k_i^{out} + k_i^{in})(k_i^{out} + k_i^{in} - 1) - \sum_{j \in N} a_{ij} a_{ji}} \tag{4.14}$$

By definition, the clustering coefficient is defined only for $k_i \geq 2$, otherwise it is identically zero. It can be proved [55] that such a quantity is equivalent to the fraction of nodes that, in triplets, are close to each other. The average of this coefficient throughout the network reflects the presence of a strongly centralized connectivity around one or more nodes (or areas).

Following the definition of the clustering coefficient, we note that this indicator is, indeed, strongly influenced by the presence of low degree nodes, being normalized node to node. A variation of this coefficient, the *transitivity*

$$T = \frac{2 \sum_{i \in N} t_i}{\sum_{i \in N} k_i (k_i - 1)} \quad , \quad {}^{w}T = \frac{2 \sum_{i \in N} {}^{w}t_i}{\sum_{i \in N} k_i (k_i - 1)} \tag{4.15}$$

$$T^{\rightarrow} = \frac{2 \sum_{i \in N} t_i^{\rightarrow}}{\sum_{i \in N} \left[(k_i^{out} + k_i^{in})(k_i^{out} + k_i^{in} - 1) - 2 \sum_{j \in N} a_{ij} a_{ji} \right]} \tag{4.16}$$

is normalized with respect to all nodes in the network, and so is not affected by such a dependency [56]. Note that the transitivity is not defined for the single node, but for the entire network.

More sophisticated measures of segregation not only describe the presence of densely interconnected cortex areas, but can even reconstruct the exact size and composition of these groups. The latter variant, the composition, is determined by the *modular structure* subdividing the network into groups of nodes having the highest possible number of inter-connections and the minimum possible number of extra-connections [57].

However, the level in which the network can be divided into these subgroups (which should, in any case, be non-overlapping) is measured by the *modularity* feature [58], that despite all other measures, is not calculated exactly but via optimization algorithms [59], which generally sacrifies a few

degrees of accuracy in favor of calculation speed.

If we denote by M the set of all the non-intersecting modules the network can be divided in and by e_{uv} the total fraction of link connecting the modules u and v, then the modularity Q is defined by the relation

$$Q = \sum_{u \in M} \left[e_{uu} - \left(\sum_{v \in M} e_{uv} \right)^2 \right] \tag{4.17}$$

In a completely equivalent way we can reformulate the expression of Q according to Newman [60], getting

$$Q = \frac{1}{l} \sum_{i,j \in M} \left(a_{ij} - \frac{k_i k_j}{l} \right) \delta_{m_i, m_j} \tag{4.18}$$

where m_i and m_j represent two modules contained in M, l is the total number of links and δ_{m_i,m_j} is the usual Kronecher delta. From this expression it is possible to derive the form of modularity coefficient for weighted and directed network:

$${}^{w}Q = \frac{1}{{}^{w}l} \sum_{i,j \in M} \left(w_{ij} - \frac{{}^{w}k_i \cdot {}^{w}k_j}{{}^{w}l} \right) \delta_{m_i, m_j} \tag{4.19}$$

$$Q^{\rightarrow} = \frac{1}{l} \sum_{i,j \in M} \left(a_{ij} - \frac{k_i^{out} \cdot k_j^{in}}{l} \right) \delta_{m_i, m_j} \tag{4.20}$$

As Newman's algorithm [60] is very fast and accurate, it is optimized for networks in whitch the number of nodes does not exceed a few units (at most, a dozen), while for our purposes (over 60 nodes) the algorithm recently developed by Blondel [61] ensures a reliable performance for large networks but sacrifies too much computation time because of its ability in highlighting any hierarchical structure within the modules (smaller modules within larger modules). The most obvious solution has been to use both of them depending on the size of the module identified within the network.

However, as we will see in the section concerning *centrality*, for our purposes it is not imperative that modules remain always separated from each

other, and indeed will be important to identify those nodes (or cluster of nodes) playing a junction role between two or more modules: it will therefore be necessary to consider the hypothesis that one or more nodes belong simultaneously to two or more modules. In this case the algorithm developed by Palla [62] seemed to us the most appropriate for such a description, keeping in mind the fact that the regions of the cortex that we consider in our studies are not exactly juxtaposed zones, but show a minimal overlapping (as it is evident considering the names of the electrodes that define their position on the scalp).

Finally, we define the concept of *Local Efficiency* of a node (or a cluster of nodes) as the tendency of that node to communicate with its neighbors using the shortest possible path.

So let $d_{jk}(N_i)$ the length of the shortest path from j to k passing only through the neighbors of node i. The *local efficiency* of node i is defined in the three variants as

$$E_{loc,i} = \frac{\sum_{j,h \in N, j \neq i} a_{ij} a_{ih} \left[d_{jh}(N_i)\right]^{-1}}{k_i(k_i - 1)} \rightarrow E_{loc} = \frac{1}{n} \sum_{i \in N} E_{loc,i} \tag{4.21}$$

$$^{w}E_{loc,i} = \frac{\sum_{j,h \in N, j \neq i} \left(w_{ij} w_{ih} \left[d^{w}_{jh}(N_i)\right]^{-1} \right)^{1/3}}{k_i(k_i - 1)} \rightarrow {}^{w}E_{loc} = \frac{1}{2} \sum_{i \in N} {}^{w}E_{loc,i} \tag{4.22}$$

$$E^{\rightarrow}_{loc,i} = \frac{\sum_{j,h \in N, j \neq i} (a_{ij} + a_{ji})(a_{ih} + a_{hi}) \left(\left[d^{\rightarrow}_{jh}(N_i)\right] + \left[d^{\rightarrow}_{hj}(N_i)\right] \right)}{(k_i^{out} + k_i^{in})(k_i^{out} + k_i^{in} - 1) - 2 \sum_{j \in N} a_{ij} a_{ji}} \rightarrow E^{\rightarrow}_{loc} = \frac{1}{2n} \sum_{i \in N} E^{\rightarrow}_{loc,i} \tag{4.23}$$

4.4.3 Functional Integration measures

Functional integration refers to the ability of the scalp areas to rapidly recombine together specialized information coming from different and separated areas. This concept is expressed in the framework of BN by means of

an estimate of the ability in witch different and separated cortex areas communicate bewteen them by considering the concept of *path*, or the set of all sequences of nodes and links that information can potentially follow to reach the node j from the node i. The distribution of these lengths expresses the intensity of the functional connection between two nodes (or areas intended as a cluster of nodes): average short lengths express strong integration between two nodes (areas), while high average lengths compete to underserved areas poorly integrated with each other. In this order of ideas, integration in functional connectivity is much less easy to interpret than the anatomical connectivity.

The mean value of the path length between each pair of nodes on the entire network is called *characteristic length* of the network and is considered the most important integration measure [55].

Called L_i the average distance between node i and *all* the other nodes in the network, the *characteristic path length* is defined, in its binary version, as

$$L = \frac{1}{n}\sum_{i\in N} L_i = \frac{1}{n}\sum_{i\in N}\frac{\sum_{j\in N, j\neq i} d_{ij}}{n-1} \tag{4.24}$$

while the weighted and directed version, we have:

$${}^{w}L = \frac{1}{n}\sum_{i\in N}\frac{\sum_{j\in N, j\neq i} {}^{w}d_{ij}}{n-1} \tag{4.25}$$

$$L^{\rightarrow} = \frac{1}{n}\sum_{i\in N}\frac{\sum_{j\in N, j\neq i} d_{ij}^{\rightarrow}}{n-1} \tag{4.26}$$

It is important to note that, while for the binarized version of the connection matrix the value of the characteristic length is evaluated by adding the active links from node i to node j, for the weighted version the *bond lengths* are added, being the latter quantity inversely proportional to the weight of each link, as larger weights are indicative of stronger ties and, as a result, smaller characteristic path and an higher "closeness" of two nodes.

The inverse of the average length is defined as the network *global efficiency* [63], that for the single node can be calculated as

$$E_i = \frac{\sum_{j \in N, j \neq i} d_{ij}^{-1}}{n-1} \tag{4.27}$$

(d_{ij}^{-1} is always the inverse matrix of distance between two nodes, but in this case we deal with the average over the entire network), so the global efficiency of the whole network is

$$E = \frac{1}{n} \sum_{i \in N} \frac{\sum_{j \in N, j \neq i} E_i}{n-1} \tag{4.28}$$

and similarly for weighed and direct versions:

$$^{w}E = \frac{1}{n} \sum_{i \in N} \frac{\sum_{j \in N, j \neq i} \left(d_{ij}^{w}\right)^{-1}}{n-1} \quad , \quad E^{\rightarrow} = \frac{1}{n} \sum_{i \in N} \frac{\sum_{j \in N, j \neq i} \left(d_{ij}^{\rightarrow}\right)^{-1}}{n-1} \tag{4.29}$$

Unlike the *path length*, the global efficiency can also be calculated on partially disconnected network: the path length, in fact, is defined as "infinite" in case of two disconnected nodes, and this leads to both an infinite characteristic length and to null efficiency. In general, the *path length* turns out to be strongly influenced by longer routes, while the global efficiency is mostly influenced by short ones. According to some authors [64] this makes the global efficiency a much more effective and significant measure of functional integration.

In general, it is possible to demonstrate [53] that effective networks are statistically more globally efficient if compared to functional networks, which show a lower integration between different modules.

4.4.4 Small-world Connectivity

The different instances of segregation (strong hierarchical structure in information processing and a few extra connections) and integration (strong interconnections between modules) are studied by the so-called small-scale

connectivity (or *small-world brain connectivity*), which measures how much the behavior of a network is close to that of an ideal, that is a network presenting the same number of nodes and links as the one under examination, but whose spatial distribution ensures that the work done by highly specialized areas is efficiently redistributed toward other highly specialized areas as well.

This linking ability appears to be carried out by the anatomical connectivity, which is not indeed the subject of our research. However, a certain number of studies [65] have shown that by combining functional connectivity characteristics with that of the effective, one can bring insightes about the small scale connectivity, as both of them are able to highlight modular structures within the network and, by comparison, the effective connectivity also highlights connecting structures between separated areas: facilities which simultaneously show high segregation and integration will be caracterized by a high small scale connectivity, while others with high integration and low segregation properties will have characteristics very far from that of the ideal network.

Alternatively [66], using the definition of connectivity on the small scale, we can define the *small-worldness* of a network by comparison with an artificially generated one with random topology: a network shows small scale connectivity if it is much more clustered of another with the same characteristic path length.

Let then two networks be given, one of which being artificially generated by a computer and having a random topology, and let respectively C and C_{rand} their clustering coefficients. Let also L and L_{rand} the characteristic lengths of the two networks. We define the *small-worldness* of the first network as compared to the second in the following way:

$$S = \frac{C/C_{rand}}{L/L_{rand}} \tag{4.30}$$

If this ratio is much greater than unity, then the first network shows the peculiar characteristics of microconnectivity. The extension to weighted and directed network is trivial.

4.4.5 Motifs of the network

The global measures (ie, extended to the whole network) of the different previously regarded quantities tend to mask a series almost infinite number of topologies such as local loop, more or less extensive closed circuits and varied forms of recurrings: a simple example consists in the *triangles* that we have seen in the previous paragraphs. But the most various structures can occur, whose number is multiplied if we also consider directionality within them.

Be given the generic direct path h (consisting of n_h nodes and l_h links in any ordered sequence) within the network. h is defined as *functional motif* within the network itself if, taken a generic path in the network or any part thereof (or its sub-network), the sequence of links of the latter coincides with that of h [67]. We can define J_h as the percentage of occurrence of the pattern h in the network. These *motifs* within the network are often associated, both at the anatomical and functional level, to particular cortex activities, and their presence becomes more important as the numerosity with which they occur grows, often referred to as the *motif z-score.*

The latter quantity is defined by considering not only the network under investigation, but also a number of other artificially generated networks with a random topology, each with its own occurrence rate of the pattern h. If such a set of network presents a standard deviation $\sigma_{J_h,rand}$ for the distribution of J_h, then

$$z_h = \frac{J_h - \langle J_{h,rand} \rangle}{\sigma_{J_h,rand}} \tag{4.31}$$

Considering, however, the motif distribution around individual nodes, one can get the node *fingerprint* in the network, characterizing the functional role

of that node (or region of the cortex) which belongs [67], using the already considered motif occurrence precentage J_h:

$$F_{n_h}(h) = \sum_{i \in N} J_{h,i} \tag{4.32}$$

in which $J_{h,i}$ is the h pattern occurrence pertentage around the node i. For weighted network it is necessary to introduce the concept of *intensity* of the motif h:

$$I_h = \sum_u \left(\prod_{(i,j) \in L_h^u} w_{ij} \right)^{1/l_h} \tag{4.33}$$

where the sum is extended to all patterns where this h is present and L_h^u is the set of links in the u-th element of summation. On the basis of these quantities we define the motif z-score *intensity*:

$$z_h^I = \frac{I_h - \langle I_{h,rand} \rangle}{\sigma_{I_h,rand}} \tag{4.34}$$

and the *motif fingerprint intensity* as:

$$F_{n_h}^I(h) = \sum_{i \in N} I_{h,i} \tag{4.35}$$

It should be noted that the functional and effective networks motifs only partially coincide with those of the anatomical network, as the firsts only partially use the paths described by the latter, which is the reason why it would be more correct to separate the concept of *functional* and *anatomical motifs*, defining the firsts as the set of all possible closed paths that can be included in tha anatomical ones.

4.4.6 Centrality measures

Some areas of a network plays an important role as *hubs* of information and connection between highly specialized areas (ie, segregated), and also their role -as we shall see in the next section- becomes more and more important when due to external or internal causes, the different areas can no longer

communicate with each other. This is the paramount concept of *centrality*, which can be extended to both the cases of individual nodes and the whole areas of the network (in our case, the different cortex areas).

The first measure of centrality we can consider is the *degree*, already seen in the introduction to this chapter, having an immediate interpretation: areas with a particularly high number of connections (both incoming and outgoing) are more likely to interact and sort the information.

Degree measures can be specialized by considering the *intra-modular* and *inter-modular* variants, describing connectivity and centrality within the specialized modules (ie, the previously considered segregated areas) and between them [68].

The *intramodular z-score* is calculated, for each node, identifying which module belongs to the node i (let's call it m_i) and counting the number of links between i and all other nodes in m_i: if we denote this number by $k_i(m_i)$ and by $\bar{k}(m_i)$ and $\sigma^{k(m_i)}$ the mean and standard deviation respectively, we obtain

$$z_i = \frac{k_i(m_i) - \bar{k}(m_i)}{\sigma^{k(m_i)}} \tag{4.36}$$

$$z_i^{out} = \frac{k_i^{out}(m_i) - \bar{k}^{out}(m_i)}{\sigma^{k^{out}(m_i)}} \tag{4.37}$$

$$z_i^{in} = \frac{k_i^{in}(m_i) - \bar{k}^{in}(m_i)}{\sigma^{k^{in}(m_i)}} \tag{4.38}$$

moreover

$$^{w}z_i = \frac{^{w}k_i(m_i) - {^{w}\bar{k}}(m_i)}{\sigma^{^{w}k(m_i)}} \tag{4.39}$$

The *participation coefficient* is defined by means of the same quantities:

$$y_i = 1 - \sum_{m \in M} \left(\frac{k_i(m_i)}{k_i} \right)^2 \tag{4.40}$$

$$y_i^{out} = 1 - \sum_{m \in M} \left(\frac{k_i^{out}(m_i)}{k_i^{out}} \right)^2 \tag{4.41}$$

$$y_i^{in} = 1 - \sum_{m \in M} \left(\frac{k_i^{in}(m_i)}{k_i^{in}} \right)^2 \tag{4.42}$$

$$^w y_i = 1 - \sum_{m \in M} \left(\frac{^w k_i(m_i)}{^w k_i} \right)^2 \tag{4.43}$$

Nodes (or cluster of nodes) having high intramodular degree and low participation coefficient (known as *local hubs*) have an high probability of playing a key role in facilitating the segregation of an area, while an high participation coefficient corresponds to an high probability for that node (or cluster of nodes) to facilitate the intermodular integration (*connection hubs*).

Most of the measures regarding centrality start from the assumption that the central nodes (or areas) are present in many patterns within the network, especially the shorter ones, thus acting as a control clearinghouse for information flows. For example, the *closeness centrality* is defined as the inverse of the average shortest path between a node and all the others in network: for the i-th node it is defined by the relations

$$L_i^{-1} = \frac{n-1}{\sum_{j \in N, j \neq i} d_{ij}} \tag{4.44}$$

$$(^w L_i)^{-1} = \frac{n-1}{\sum_{j \in N, j \neq i} {^w d_{ij}}} \tag{4.45}$$

$$(L_i^{\rightarrow})^{-1} = \frac{n-1}{\sum_{j \in N, j \neq i} d_{ij}^{\rightarrow}} \tag{4.46}$$

Similarly, the *betweeness centrality* is defined as the fraction (on the total existing in the entire network) of shortest paths passing through a given node:

$$b_i = \frac{1}{(n-1)(n-2)} \cdot \sum_{\substack{h,j \in N \\ h \neq j, h \neq i, j \neq i}} \frac{\rho_{hj}(i)}{\rho_{hj}} \tag{4.47}$$

where ρ_{hj} is the number of shortest paths from h to j and $\rho_{hj}(i)$ is the number of shortest paths from h to j that passes through i. We can get the weighed and directed versions simply by replacing

$$\begin{cases} \rho_{hj} \rightarrow {}^{w}\rho_{hj} \ \vee \ \rho_{\vec{hj}} \\ \rho_{hj}(i) \rightarrow {}^{w}\rho_{hj}(i) \ \vee \ \rho_{\vec{hj}}(i) \end{cases} \tag{4.48}$$

In this sense, nodes with a very high betweeness centrality behave as a bridge to connect two or more nodes otherwise separated. Naturally, the concept can be extended to links, not only to nodes, and in this way we can get two variants of the betweeness centrality: the *Vertex Betweeness* (VBC) and the *Edge Betweeness* (EBC), both of them calculable with fast algorithms such as those of Brandes [69] and Kintali [70].

It should be noted how anatomical nodes showing a strong centrality are also those who, connecting regions otherwise disconnected between them, facilitate their integration, increasing their functional connection. At the same time, however, these anatomical links make the functional centrality of these nodes less important, which are consequently less easy to spot.

4.4.7 Resilience Measures

Resilience is defined as the potential ability of a network to recover its functionality as a result of interruptions (pathological, structural or whatever) of the connections between the various constituting modules: in fact, the deterioration of the anatomical connections between brain areas inevitably leads to a similar deterioration in functional and effective connectivity both among the directly injuried areas or even between areas using that one as an information hub. The analysis of the network allows us to highlight those network areas that, in case of such a damage, are able to restore the lost

connectivity.

The resilience measures can be subdivided into two classes: direct and indirect ones. By their nature, direct measurements [71] require long periods of observation and experimentation for the measurement to be performed, as they test the actual adaptation of the network to the progressive deterioration of anatomical and, by consequence, functional connectivity. In this case, which is not of our interest, the applicability of the chosen measures has to be possible on disconnected network, as one or more areas can evolve this way over time.

The resilience measures of our interest, however, are the indirect ones, which test the potential capacity of recovery of networks and areas presenting an high risk of deterioration.

One of such a measures is the *degree distribution* of the network [72]. If $p(k_i)$ is the probability that the node i has degree k_i, then the *degree distribution* can be defined as follows:

$$P(k) = \sum_{k' \geq k} p(k') \tag{4.49}$$

and the weighted and direct versions can be trivially obtained. Nodes or areas presenting low grade power laws distributions may be more resilient to deterioration of the random networks, but at the same time are more vulnerable in case of damage of high egree central nodes.

As the real network does not always follow a defined grade power law, it is possible that some modules inside it present defined degree distribution (most of the times, a low grade). The analysis of these areas can result in some cases useful the entire network resilience study [64].

A second measure that can effectively characterize the resilience of a network is the *assortativity coefficient*, a measure of the correlation between

the degree of the nodes at the beginning and at the end of an information path:

$$r = \frac{l^{-1}\sum_{i,j\in L} k_i k_j - \left[l^{-1}\sum_{i,j\in L} 1/2\left(k_i + k_j\right)\right]^2}{l^{-1}\sum_{i,j\in L} 1/2\left(k_i^2 + k_j^2\right) - \left[l^{-1}\sum_{i,j\in L} 1/2\left(k_i + k_j\right)\right]^2} \tag{4.50}$$

$${}^{w}r = \frac{l^{-1}\sum_{i,j\in L} w_{ij}\cdot {}^{w}k_i\, {}^{w}k_j - \left[l^{-1}\sum_{i,j\in L} 1/2 w_{ij}\left(k_i + k_j\right)\right]^2}{l^{-1}\sum_{i,j\in L} 1/2 w_{ij}\left({}^{w}k_i^2 + {}^{w}k_j^2\right) - \left[l^{-1}\sum_{i,j\in L} 1/2\cdot w_{ij}\left({}^{w}k_i + {}^{w}k_j\right)\right]^2} \tag{4.51}$$

$$r^{\rightarrow} = \frac{l^{-1}\sum_{i,j\in L} k_i^{out} k_j^{in} - \left[l^{-1}\sum_{i,j\in L} 1/2\left(k_i^{out} + k_j^{in}\right)\right]^2}{l^{-1}\sum_{i,j\in L} 1/2\left((k_i^{out})^2 + (kin_j)^2\right) - \left[l^{-1}\sum_{i,j\in L} 1/2\left(k_i^{out} + k_j^{in}\right)\right]^2} \tag{4.52}$$

The positive assortativity presupposes that the two ends of the paths consist of nodes (or clusters, particularly hubs) interconnected between them and of comparable resilience; vice versa, a negative coefficient expresses the possibility that the central nodes are uniformly distributed in the network and that, consequently, their eventual removal makes vulnerable the entire network generating an unstable behavior.

Two other measures that may characterize the resilience of a network are the *local assortativity coefficient* (ie, a localized version of the coefficient of assortativity, only extended to modules or areas) and the *average neighbor degree* [73]:

$$k_{nn,i} = \sum_{j\in N} \frac{a_{ij}\cdot k_j}{k_i} \tag{4.53}$$

$${}^{w}k_{nn,i} = \sum_{j\in N} \frac{w_{ij}\cdot {}^{w}k_j}{{}^{w}k_i} \tag{4.54}$$

$$k^{\rightarrow}_{nn,i} = \sum_{j \in N} \frac{(a_{ij} + a_{ji})(k_i^{in} + k_i^{out})}{2(k_i^{out} + k_i^{in})} \tag{4.55}$$

Low values of these indicators show a high risk of impairment for the entire nestwork if the nodes which are referenced were removed.

Chapter 5

EEG Data analysis

5.1 Introduction

The tools we have presented up to this point can be applied to various fields of research and inference analysis, ranging from econometric data (for which they were originally developed, although not all of them) to the weather data, and so on.

The analysis of electroencephalographic data is intended to highlight, through the techniques set out above, any differences which may exist between two or more populations of migraine patients in terms of total quantity of information flowing between different cortex areas or efficiency that they may have in distributing, processing it and so on.

For this purpose, it becomes necessary to consider at least two populations in each study, one of which must be considered as a reference for the analysis (in the present case, the so-called "controls", or patients not suffering the disease under study). Moreover, the choice of the particular stimulation, of course decided by the neurologist, has been made time by time in such a way as or to stimulate areas particularly suited to recipe it (for example, visual stimulation, which affects the parieto-occipital areas, is used to highlight any cognitive deficiencies of a population or to verify that, in migranic patients, their relative neuroelectric activity is more intense than the others, as it is

well known in neurology), or to affect as many areas as possible in order to verify the integration state of the network (it is the case of painful laser stimulation that, by acting directly on the brainstem system and on pain receptors, arrives directly to the cortex without passing by one of his areas).

5.2 Characterization of EEG and *10-20* system

The data considered in this work, as widely mentioned in previous chapters, are of biological origin, and precisely they constitute the recordings of electrical potentials measured at specific sites of the human scalp, arranged in such a way as to cover as much as possible the entire extension of it (the so-called "channels"), each of which records a signal whose magnitude is a few tens of μV and whose value is the *sum* of all the potentials underlying the cortex area covered by the electrode.

Such a system of data is called *electroencephalogram* (EEG), and for the purposes of this research it is a system of 64 channels with a *sampling rate* of 256 points per second, which makes the cortex EEG analysis of neuroelectric activity an high temporal resolution analysis. In reality, not all of these channels are used in the analysis: two of them in fact, 63 and 64, only controls the so-called *electro-oculogram*, EOG, used to control the eyes movements and one, number 32, is the recording of the electrocardiogram (ECG); the number of usable channels falls then to 61.

The need for using the EOG derives from the fact that each movement of the patient undergoing stimulation and EEG recording may introduce artifacts in the recording the same that can make it completely unusable around the event. The most common of these artifacts is the *eye-blinking*, which creates evident spikes in the recording: the registration of the eye movement makes it possible to check for any interval of time that must be removed from *all the channels of the track* in the pre-processing phase, being the coregis-

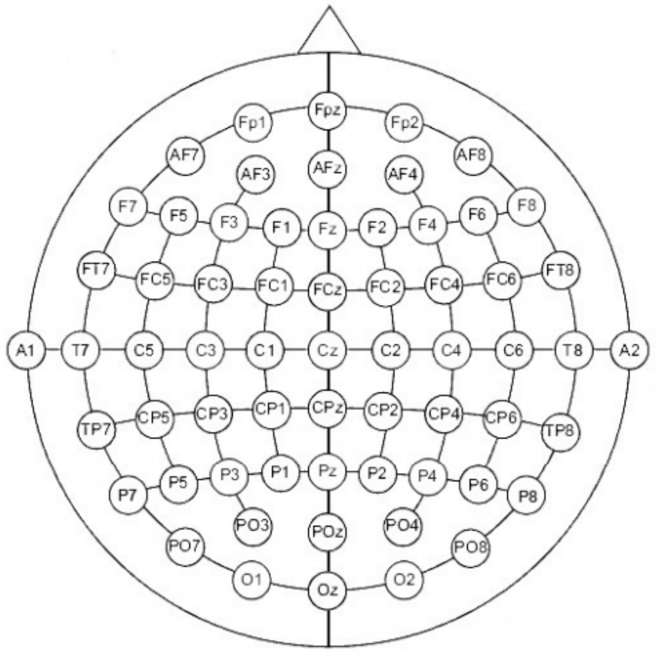

Figure 5.1: the *10-20* system for mounting the electrodes.

tration of the time series of primary importance, as noticed, for the analysis of TE, GC and all other indicators.

Our first work, however, has been conducted using only a 12 channels system for data recording, only 6 of which almost free from artifacts to be taken into account, as a result of an older registration system with respect to the one that has been adopted later. However, both recording systems share the arrangement of the common electrodes, for which the system consisting of 61 channels can be considered to all effects as an extension of the one with 20 channels, and is indeed a model with an higher spatial resolution.

An important consideration is to be done. As it is well known, not all skulls are equal, and if proper precautions are not used, the risk of recording the neuroactivity of cortex areas that can not be related to the specific areas indicated with reference to the name of the electrode (F1, T8 and so on, as

visible from figure 5.1) is quite high. To overcome this problem it has been constructed the so-called *10-20* system for the system with 20 channels, which in the case of 64 channels is called *extended 10-20 system*, which covers up to a maximum of 128 channels, which we will not use. Such a model assumes that the electrodes are placed (individually or embedded in a suitable cap) in a sequential way, starting from two particular points of the head that are located on the median line connecting the point at the center of the frontal bone (or eyes, called *nasion*) with the point to its opposite in the occipital area (*inion*). In this way, the first five electrodes are fixed on the midline equidistant between them in such a way that the edges are situated at a distance from the two reference points equal to 10% of total distance, and the other at 20% of the total distance between them. The same happens along the sagittal direction, ie from ear to ear (the reference point, in this case, are singularly called *trago*). In the intersecting point of the two medial lines, called *vertex*, the electrode attached to the top of the skull, called Cz, takes place.

All the other electrodes are fixed in such a way to consider the distance between two of the previous electrodes as a reference and to leave constants the relative distances between each pair of electrodes. In this way, the actual position of the electrodes reflects the one they would have on an "ideal" head, and especially it can be said to be common to all shapes and dimensions of the skull, as widely demonstrated in the medical literature [74].

5.3 Frequency Bands

As all the signals varying in time, the EEG too can be considered as the sum of different contributors sine wave at different frequency (or pulse). However, a *spectral* characterization would not be easy to treat, since the number of sinusoidal components of the signal is theoretically infinite.

It has been thought, therefore, to use not the individual frequencies as

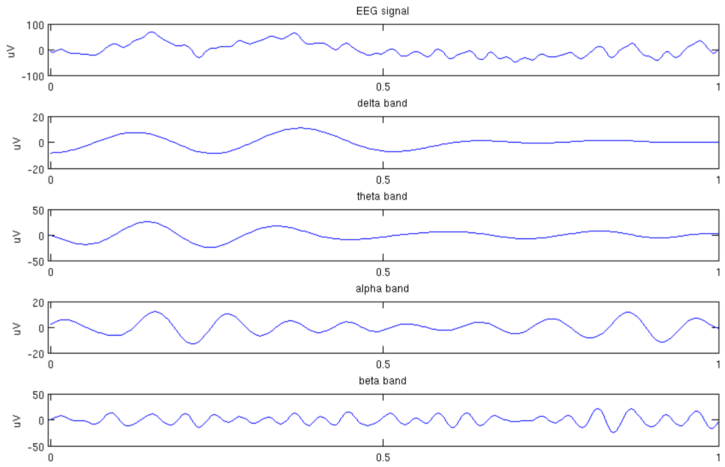

Figure 5.2: filtering of the EEG signal (top) in the four frequency bands. In abscissa, the time is measured in seconds.

a reference, but *frequency bands*, each of which is characterized by a certain range of frequencies and that reflect, individually, a determined type of cortex activity (figure 5.2): from *slow-waves* or *longwaves* which reflect the activities of the oldest areas of the brain (the *amygdala*, for example) to the *rapid* activity which is expressed for example when the cortex, in a steady-state, must recognize particular types of stimulation or perform specific tasks.

Specifically, there are four to six bands, depending on the conventions on two sub-bands. This convention provides the following distinction:

- δ band, from 0.5 to 4 Hz is the bandwidth of long waves and unconscious activity, associated generally to deep sleep;
- θ band, from 4 to 7 Hz associated with light sleep or drowsiness;
- α band, from 7 to 12 Hz is the first band of consciousness associated with the resting state and relaxation with eyes closed while awake conditions;

- β band, from 12 to 30 Hz, is the bandwidth related to the neuroactivity of waking and attention.

This is the four-band system that will be used from now on, but it is not the only possible choice: some authors prefer to split the α band in two equiextended bands, α_1 and α_2, other authors prefer to include the γ band, with frequencies from 30 Hz on, but belonging only to frontal areas. Obviously the choice is of convenience and reflects the specific needs of a given research work. Moreover, the separation of the bands is not always so clear: their thickness is often extended right and left of each interval of 0.5 Hz, so as to obtain not separated bands, but intersecting.

The localiztion of the brain *waves* is not a prerogative of the γ band only: even β waves are present in the front and central areas almost exclusively. In addition, the alteration of some frequency component is closely related to specific disorders: the alteration of δ rhythms can be an indicator of a medium-to-severe disorder of brain activity, while the alteration of θ rhythms is often a sign of a mild-to-moderate disorder of cortical activity. However, this topic is beyond the scope of this work, and for a more in-depth case studies, please refer to specialized medical literature.

The choice of the bands to be used (all the four bands, or only α and β, and so on), however, is not always related to the type of stimulation performed on the patient: the flashing light at a certain frequency will not affect necessarily the frequency bands in which it is located but even its neighbors, in something like a resonance effect, as well as a laser stimulation painful not only affect the lower and the higher band, being the processing bands of the stimulus during wakefulness (β band) and pain (δ band): it will be seen in fact, as the neural response interests quite all the frequency bands.

5.4 Data pre-processing

Before analyzing the available EEGs, it is necessary to clean up data from all kind of artifact that could lead to significant errors both from the point of view of measurement of the real quantities, both from the one of results comparison among populations (patients suffering from a specific pathology, healthy patients, etc.).

Following the procedures universally adopted by the scientific community, the technician responsible for carrying out the recording session and the neurologist have first applied a digital band-pass filter in the range of frequencies between 0.1 and 70 Hz, and then applied a Notch filter to remove interferences and harmonics at 50Hz from the power line that may have affeted the recording.

Finally, an electrode has been chosen to be the reference for the measurement of potential differences, namely Fpz. The electrodes used presented a total impedance of less than 10 $k\Omega$.

The band filtering was performed using a two-tailed Butterworth filter of the second order.

5.5 Potentials evoked by visual stimulation (SVEP): flashing light

In the studies performed on functional and effective connectivity of migraine in all its various forms, the first two types of stimulation which patients were subjected to were those from visual flashing light, belonging to the category of SVEP: "Steady-state Visually Evoked Potential". The first type of stimulation considered will be the simple flash with different time frequencies.

The available EEGs were from 19 patients suffering from migraine with

aura (MA) selected in accordance with the ICHD-II procedure [75], 19 subjects experiencing migraine without aura (MO), whose diagnosis was made according to the same procedure, and 11 healthy subjects (N), selected among the nursing staff of the Neurology Department.

Analysis of statistical inference (ANOVA and χ^2 tests) have shown that there are not imbalances in populations in favor of male or female and there were no peacks about particular values of the age distribution. It has also been verified that the MA and MO patients were temporally distant from the last migraine attack and sufficiently furthest from the next, in such a way as to exclude any effects due to the pre-attack dysfunctional state. Such a condition has been verified by a telephonic interview after the recordings have been perfromed.

The visual stimulation has been conducted through an intermittent strobe with radiant energy of 0.29 J, located 20 cm from the patient, who was asked not to close eyes during stimulation.

Five different frequencies of stimulation have been used: 9, 18, 21, 24 and 27 Hz, whose choice was not casual, but based on previous physical and neurological studies [76, 77]. Each of them was delivered to patients for about 40 seconds, with an interval of 20 seconds between them, so as to be certain that the residual effect does not interfere with the next. For each stimulation about 20 trials were performed.

The potentials were recorded through six electrodes: two occipital (O1 and O2), two parietal (P3 and P4), one central (Cz) and one frontal (Fz), the assembly of which was referred to *nasion*.

The frequency bands used in this first work were only the highest two, α and β.

5.5.1 Analysis of the spectral power

Using the Fast Fourier Transform for discretized signals (FFT), and selecting from time to time the signal strength at the stimulation frequency, it was found that, in correspondence with this frequencies, the EEG signal of MA and MO was significantly more powerful than that of N, in all channels except those in the fronto-central region (Fz and Cz, figure 5.3).

5.5.2 Analysis of functional connectivity: Synchronization Entropy

Once we assigned the two weights: $(m, n) = (1, 1)$, it has been possible to proceed with the phase synchronization analysis. In order to reduce the number of multiple comparisons and then lower the Bonferroni correction factor (see later), we proceeded comparing, for each stimulation frequency, not the absolute synchronization factor but the one relative to the base, defining the following factor for each band

$$\Gamma = \rho^{flash} - \rho^{spont} \tag{5.1}$$

where ρ^{flash} is the average synchronization of the cortex in the presence of light stimuli and ρ^{spont} is the base mean spontaneous synchronization in that band. This difference measures the change in synchronization with the arrival of the light stimulus in relation to the activity of the base, and is called *hypersincnronization.* [76]

The statistical analysis conducted with the t-Student test with post-hoc Bonferroni correction showed that in α band, the MO show a clear hypersincnronization compared to the MA and the N in almost all frequencies of stimulation (figure 5.4, top panel). ANOVA test with post-hoc correction showed that the stimulation frequencies have different effect on synchronization in α band, even if the post-hoc test showed no significant differences between the various frequencies, particularly for MO patients compared with the MA and the N [78, 79].

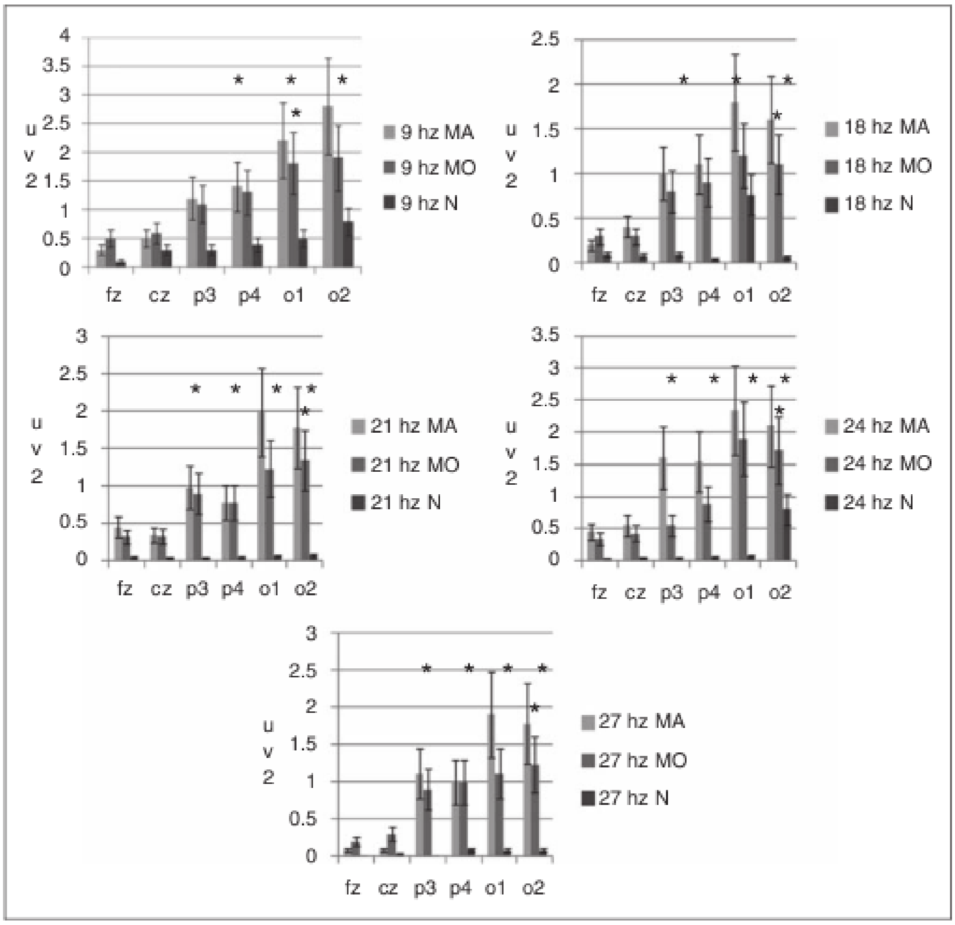

Figure 5.3: power spectrum analysis of the EEG signal at the stimulation frequency. Bars marked with an asterisk indicate statistically significant comparisons.

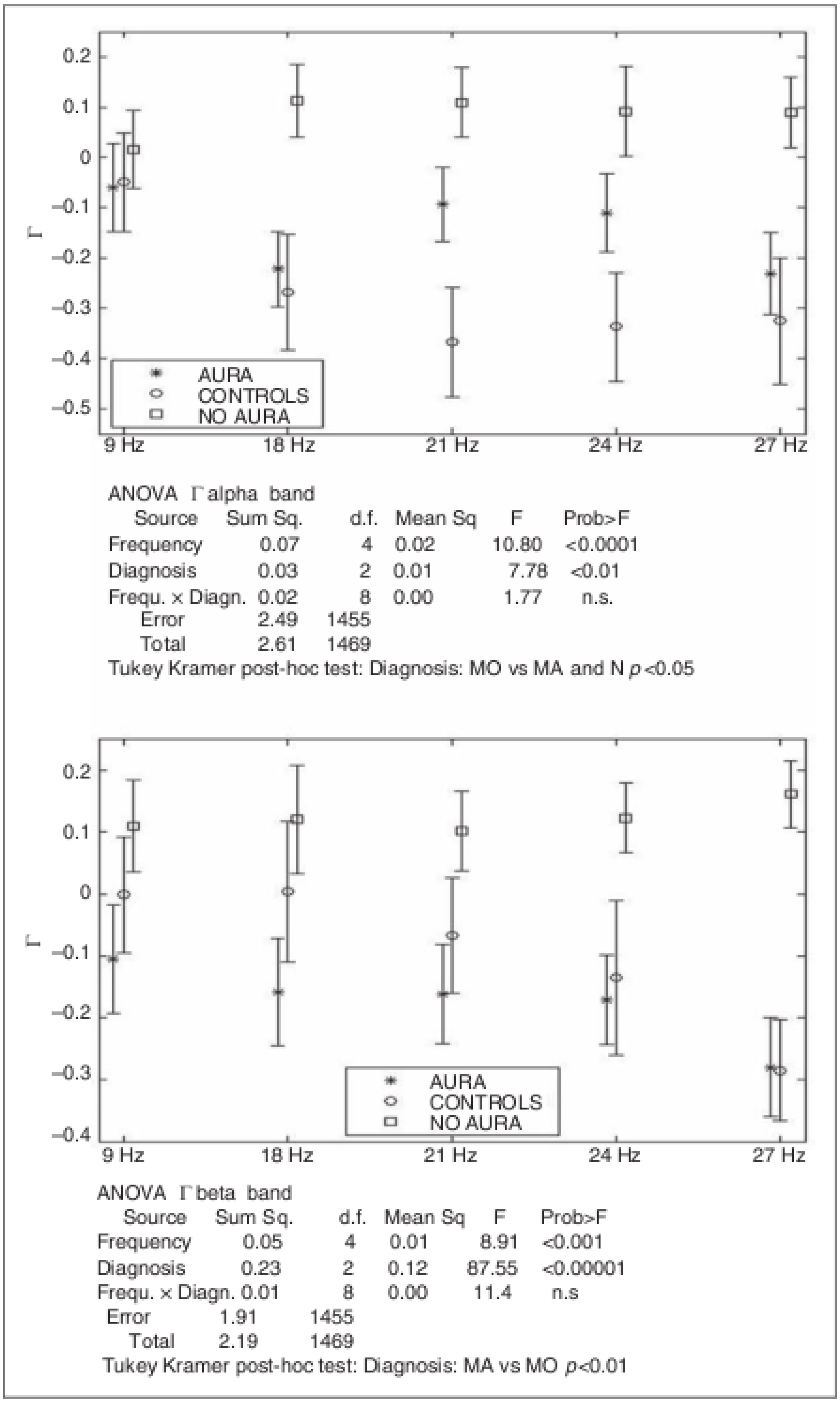

Figure 5.4: mean values and standard deviations of the synchronization index Γ in α (top panel) and β (bottom panel) bands. At foot of each panel the values of the corresponding ANOVA tests are reported.

In β band, however, we are witnessing the effect of desynchronization of MA with the increasing stimulation frequency (figure 5.4, bottom panel), an effect that is also present for the N, although with less intensity.

There is also a clear difference between the two groups of patients with migraine: the MA de-synchronize faster with the increasing frequency of stimulation than in the case of MO.

5.5.3 Analysis of effective connectivity: Granger Causality

For the analysis of effective connectivity we chose to use the non-linear GC, using a Gaussian kernel. The results obtained for each pair of channels and for each population were averaged and compared with a one-way ANOVA with post-hoc correction. The magnitude of the connection was significantly different across populations and stimulation frequencies in α band: the MO showed values significantly lower than the MA and the N (figure 5.5, top panel), with the frequency of stimulation at 24 Hz which also shows an effect of interaction between populations and stimulation frequencies, which makes the three populations even more separated basing on the mean values of causality.

In β band, the MA show larger average values of causal connection than MO and N in all frequencies of stimulation, with the other two populations showing causality values practically constant throughout the range of frequency stimulation (figure 5.5, bottom panel).

Finally, a further ANOVA test showed that there were significant correlations between SE and GC values in all bands and all frequencies of stimulation.

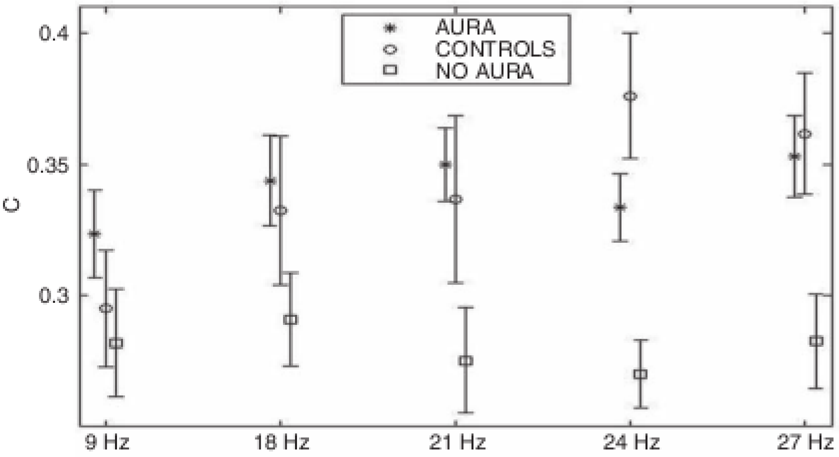

ANOVA causality alpha band

Source	Sum Sq.	d.f.	Mean Sq	F	Prob>F
Frequency	0.16	4	0.04	5.98	<0.001
Diagnosis	1.27	2	0.64	92.50	<0.00001
Frequ. × Diagn.	0.24	8	0.03	4.32	<0.001
Error	10.01	1455			
Total	11.63	1469			

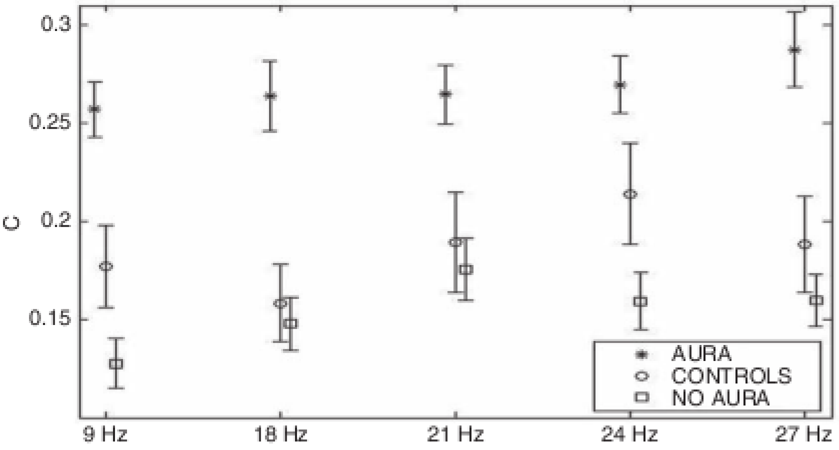

ANOVA causality beta band

Source	Sum Sq.	d.f.	Mean Sq	F	Prob>F
Frequency	0.21	4	0.05	10.08	<0.0001
Diagnosis	1.27	2	0.64	192.50	<0.00001
Frequ. × Diagn.	0.24	8	0.03	2.92	<0.001
Error	10.01	1455			
Total	11.63	1469			

Tukey Kramer post-hoc test: Diagnosis: MA vs MO P<0.001 MA

Figure 5.5: mean values and standard deviations of the causality index C in α (top panel) and β (bottom panel) bands. At foot of each panel the values of the corresponding ANOVA tests are reported.

5.5.4 Conclusions

As a result of light stimulation, MA and MO show a different behavior in their neuroelectric activity. In the first population, in fact, an increase in effective connectivity in β band has been observed, while in the latter there is a similar increase in α band. Such a difference may indicate a different neurosynaptic behavior between the two phenotypes of migraine [83].

5.6 Evoked potentials from photic checkerboard

The second type of light stimulation that has been considered was the one with bright intermittent checkerboard with two different spatial dimensions: the first with 5 mm edge and the other with 20 mm; the stroboscopic frequency was always kept at 5 Hz.

Such kind of stimulation was suggested by neurologist in accordance with Shibata hypotesis [80], in wich a photic checkerboard stimulation could lead to important differences in cortical activity of migranic patients.

The EEG used in the connectivity analysis consisted of 61 channels recordings of neuroelectric activity during the visual stimulation, whose temporal extension was about 5 seconds. In addition to these, also the unstimulated neuroelectrical activity recording, the so-called *bases*, were available.

The sampling rate was 256 Hz, and the recordings were made by placing the stroboscopic checkerboard, whose radiant energy was 0.29 J, 20 cm from the patient, which were asked to keep eyes wide open during the stimulation without blinking their eyes.

The patient's populations voluntarily undergoing stimulation were three: migraine with aura (MWA, 29 patients), migraine without aura (MWoA, 19 patients) and patients not suffering from migraine (CONT, 11 subjects).

Based on these considerations, the statistical analysis was conducted, for each channel and for each band, using the one-tail (alterned left and right) t-Student test with a Bonferroni correction for multiple comparisons equal to

$$b = n_{couples} \times (n_{stims} - 1) = 3 \times 2 = 6 \tag{5.2}$$

The Bonferroni correction is a *post-hoc* compatibility test, used to increase the confidence level in comparisons for a difference to be considered as statistically significant, and is performed when the number of comparisons is greater than one: in this case the universally accepted value of 0.05% c.l. is lowered to 0.05% / b (in our case, 0.008%, or 0.004% for each tail).

In this type of analysis, we decided to study the behavior of populations for each channel (or couple of them), without performing averages as in the case of the bright flashes: the larger number of channels, in fact, allows an higher spatial resolution compared to the previous case, and gives us the possibility of studying the effects of the behavior of every single cortex area regardless that of the other. Such a condition would not be highlighted if we would have take the mean value over the whole 61 channels parcellation of the scalp, hiding possible (and, as a matter of fact, real) differences in single areas behavior that might distinguish populations among them.

In this case, however, a new type of graphycs have been studied to explain numerical results, in order both to avoid dealing with a large number of plots of the type already seen in the previous work (in the best case, 61 plots; in the worst, 61 $\times$ 61 = 4096 plots for each stimulation and for each comparison: this condition is, of course, unapplyable) and to fournish an immediate identification and intensity of the distinguishing areas of the cortex.

In this context, we decided to use the *topoplot* system to visualize results. Such a system is easy to understand: the human head is pictured

from above, and each single channel stands for its particular position on it. The convention we used is the following: if the comparison on that particular channel gives no distinction, it remains green in color. If, instead, the channel gives rise to distinction, we can deal with two different pictures: the first is used in the functional/effective analysis, in which the distinguishing channel's color is as deep as the number of the external channels which the channel communicate with increases; in the second, mainly used while dealing with BN features, each distinguishing channel's color depth is as deeper as the percentual difference among populations increases.

The color convention we used is the following: warm colors if the distinction in in favour of the first element of the comparing couple (eg, in MA/MO case, red is referred to MA), cool colors otherwise (in the last example, blue is the distinction in wich MO levels are larger).

In this way only we can deal with few diagrams fournishing both as much information as possible and a clear location of the distinguishing areas.

5.6.1 Analysis of effective connectivity: Transfer Entropy

By means of Kolmogorov-Smirnov test we verified that the data distribution for each channel were parametric and, with the subsequent Spearman post-hoc test, if the gaussian trend of their distribution was verified. Only at this point we was able to carry out the statistical analysis of data.

The first parameter that must be fixed in the study of effective connectivity is the *lag*, which affects strongly the value of cortical connectivity. An analysis of the average values of the TE as a function of the lag has shown (figure 5.6) that within 75 ms preceeding each considered sample, the magnitude of the TE does not vary significantly. For this reason the value of 9 samples for this parameter was chosen, corresponding to about 36 ms before each single element of the series.

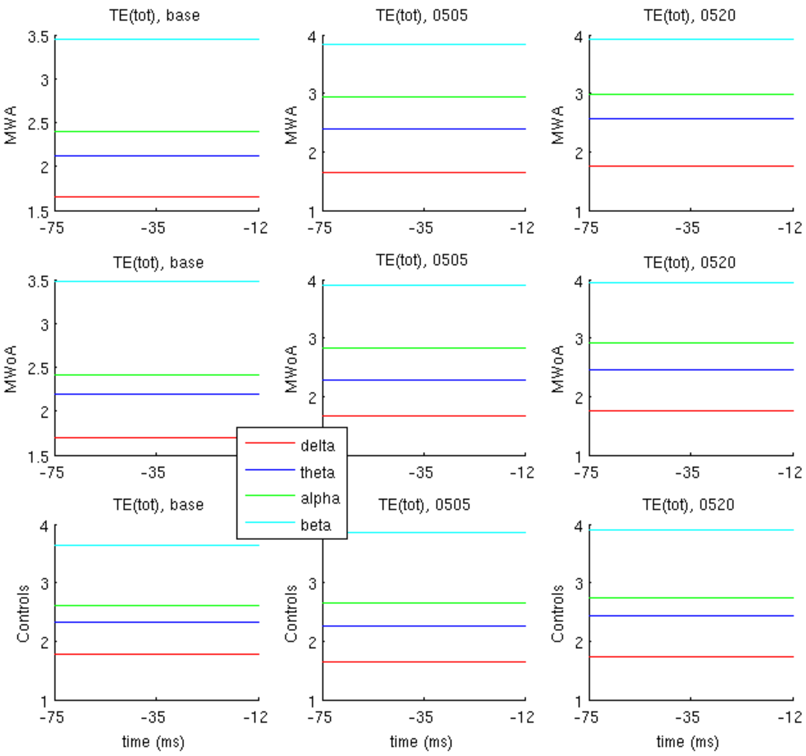

Figure 5.6: trend of TE as a function of the lag.

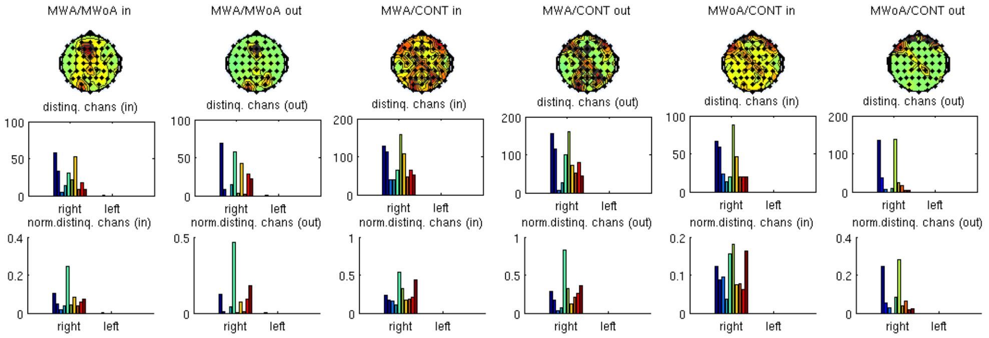

Figure 5.7: analysis of the TE in α band for checkerboard stimulation with 5 mm edge. In the first row are shown, for each recording site on the scalp, the total number of distinguishing channels, both in input and in output, for the three pairs of populations; as always, warm colors indicate that the most number of channels is in favor of the first element of the pair, while the cold colors indicate a distinction in favor of the second element of the pair. The second row shows histograms with the number of channels that, area by area, distinguish the two populations: cool colors for the left hemisphere and warm for the right; the sequence of areas is frontal, central, temporal, parietal, occipital. In the third row, the same histograms normalized with respect to the total number of channels for each area: in this case representing the percentage of each area's channels that distinguish the two populations.

Starting analyzing the base (ie, the EEG is not subjected to stimulation), we immediately notice that there were practically no differences in behavior between the MWA and MWoA; only in β band a few significant differences between the two populations are noticed, and only in the right frontal area, which presents a slight difference both in input and output (about 3 - 4%), moderately higher in MWA. At the same time, there is no difference, in any band, between MWA and CONT.

A substantial difference can be seen, however, between MWoA and CONT: the latter, in fact, always show information levels, both incoming and outgoing, larger than the first of 15% on the average, even if this distinction not always follows a precise pattern: in lower bands (δ and θ) this superior-

ity is almost uniform, while in the higher bands there is a strong posterior and right temporal component. In output, however, there was no recursive schemes.

The second type of stimulation, the one with 5 mm edge checkerboard, shows the comparisons MWA/MWoA and MWA/CONT very similar, both from the exchanged information levels point of view and from that of in/out directionality. In both cases, the two central bands, θ and α, show larger TE levels in MWA patients (up to 50%), but while in input (figure 5.7) the area of greatest distinction is always across the right and left hemispheres in the neighborhood of the *vertex*, with a structure that vaguely recalls a capital X whose center is the Fz channel, in output the two comparisons are different: the second (MWA/CONT) shows a structure similar to that just described, while the first shows the constant presence of channel Fz, from which flows information in larger quantities than in MWoA. The outer bands, however, does show a fast decrease of TE levels in MWA than the other two populations, particularly in left temporal areas.

The third type of comparison, MWoA/CONT, shows as CONT present, in the lower bands and in correspondence of Fz, mean information levels larger than MWoA up to 30%, while in the front left area it's MWoA that present larger information levels. In the uppermost band, instead, the MWoA show, in the frontal areas (right and left) larger levels of transferred information.

The 20 mm checkerboard stimulation shows, however, a marked difference between MWA and CONT in all frequency bands and with similar structures and characteristics both in input and in output: around Fz the X-shaped area is again created and clearly distinguishes the two populations (figure 5.8), with levels of TE much larger (that could even reach 90% in output) in MWA than in CONT. Such a structure would seem to tie together anterior frontal areas with those occipital, creating a sort of corridor that information can follow.

The comparison between MWoA and CONT shows characteristics very similar to what has been seen, with the X-shaped area centered around Fz

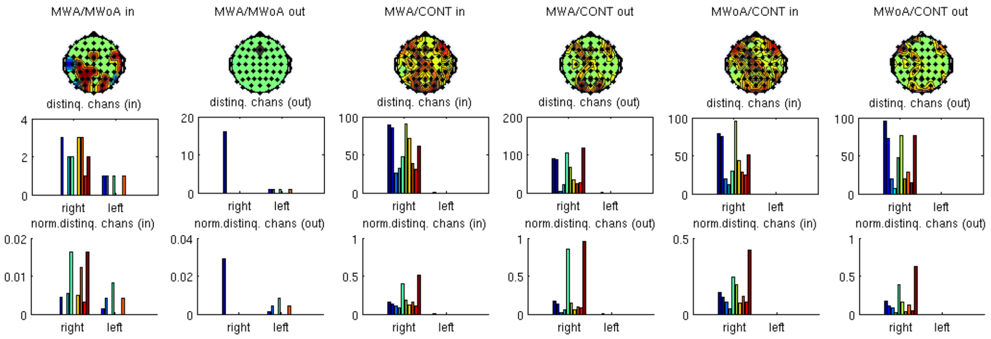

Figure 5.8: analysis of the TE in α band for checkerboard stimulation with 200 mm edge.

sending an larger amount of information (more than 60%) in MWoA that in CONT. At the same time, however, there are nor substantial differences nor easily recognizable structures that can distinguish MWoA and CONT with rspect to the outgoing information.

Finally, the comparison between MWA and MWoA shows features very similar to the smaller checkerboard: in outer bands the exchanged information level is slightly lower (about 2%) in MWA compared to MWoA almost exclusively in the left temporal areas. In the inner bands, however, the MWA show larger exchanged information level, particularly in the central and occipital. In output, however, the channel Fz is always the one showing differences, presenting the highest levels of TE in the MWA.

5.6.2 Analysis of Brain Network

The analysis of the BN began with the study of the overall behavior of the network in function of the applied threshold used to eliminate spurious connections. As we saw in section 4.3, it was decided to apply a adaptive threshold to compensate for the different connections intensities characterizing the network of each patient.

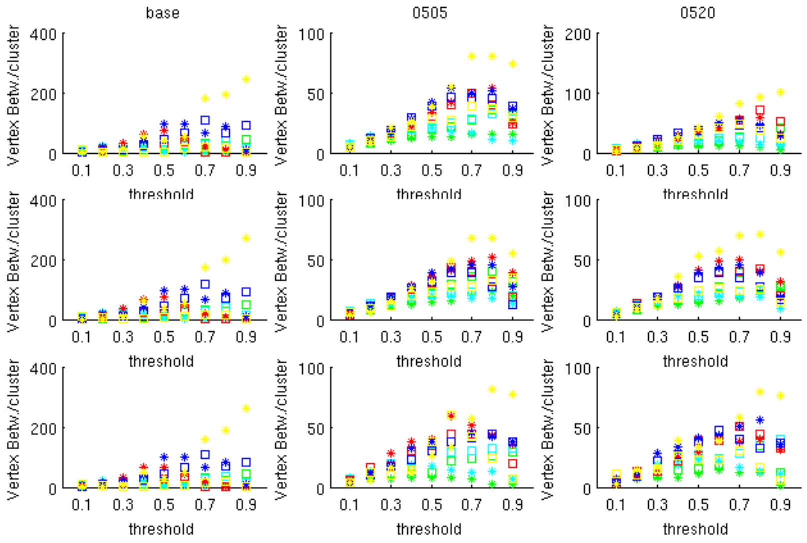

Figure 5.9: β band analysis of the Vertex Betweeness trend for the different cortical areas as a function of the adaptive threshold applied.

The result of this analysis showed (figure 5.9, in which is shown, as a general example, the trend of the Vertex betweeness) that considering an adaptive threshold of 65%, the behavior of the network indicators and their variance reaches a *plateau*. This choice means that any connection strength whose value falls below the 65% of the maximum connection value is removed from the network.

Regarding the statistical treatment of data, it was decided to use a double investigation key: on one hand we have chosen to continue using the Student's t test on each channel, so as to improve, as already said, the spatial resolution of the analysis, and on the other, it has been chosen to perform a parallel statistical comparison using the channels of a cortex area (for example, the right front, the left parietal and so on) as a single ensemble of data, in such a way that the comparison has not interested channels individually, but the entire cortex area.

So we have to manage three different cases:

- *the entire area and most of the individual channels differ significantly across populations*: the case anymore simple, since the behavior of the entire area is determined by that of the individual channels;
- *the entire area distinguishes the two populations, but none or just a few channels do the same*: in this case the variances of the individual channels in the two populations are too large for the means to be distinguished, but in the overall case, the global variance of the whole area (which is the squared sum of the channels individual variances whose the square root is extracted) may be lower than that of the individual channel, ensuring the entire area to give contribution to distinction;
- *the area does not distinguishes populations, but many of its channels do it*: the single channel has, in the two populations, a small variance compared to the indicator mean value, but is globally "drowned" in the great variability of the area's variance, that in this way do not contributes.

Furthermore, each feature calculated on the basis of the real data, before being used as a tool of a possible distinction between populations, has been subjected to a comparison with similar quantities derived from computer artificially generated connection matrices (the NM, already seen in Chapter 4) with both random and fractal topologies: real and simulated data shared, indeed, degree, number of nodes, their distribution and the average paths length, so precluding the possibility that results were due to a random distribution of nodes and links.

Integration measures

MWA/MWoA comparison The analysis of the Characteristic Path Length (CPL) does not show substantial differences between the two populations, as long as we do not consider the *eccentricity* variant that, especially in the base, shows a larger eccentricity of MWoA than the MWA (about 16%),

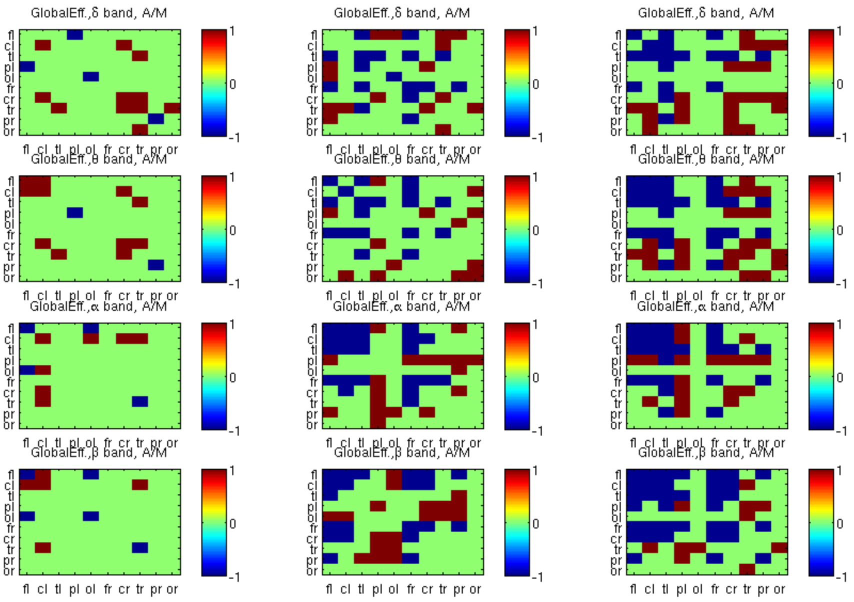

Figure 5.10: comparison of global efficiency in MWA and MWoA populations: the matrix elements in red (+1) account for larger values in MWA, while those in blue (-1) account for larger values od MWoA. Green cells mean no distinction.

which is, in the lower bands, spread all over the cortex, while in the higher ones is reduced to the left hemisphere.

Of particular interest is, instead, the study of global efficiency in different bands, particularly if the evolution with respect to the basal activity is considered. In the latter, in fact, there is no substantial difference between the two populations, and the application of stimuli (regardless of type) shows how the MWoA increase the left intra-hemispheric global efficiency with respect to the MWA, especially in fronto-central areas.

At the same time, the MWA show a larger efficiency in sorting information from the left parieto-occipital areas to right cortex areas (higher inter-hemispherical efficiency).

This effect is also characterized by a total increase of efficiency and structuring in both populations with the frequency bands (figure 5.10).

Finally, the analysis of *density* shows how, in all the bands of the basal activity, the efficiency of the MWoA's network is higher than that of the MWA of about 35%.

MWA/CONT comparison Also in this case there are no differences between MWA and CONT on the basis of CPL, but only in the *eccentricity* version, showing once again as the base distinguishes very well MWA, having lower levels with respect to CONT (about 10%). Considering the previous distinction with MWoA, we can say that the low *eccentricity* is a hallmark of migraine with aura, since the basal activity.

It is interesting to note, however, as during the stimulation, the eccentricity of the MWA in α band increases of about 15% in the central area if compared to CONT.

The comparison on the basis of global efficiency is interesting too. In the basal activity, CONT show a larger efficiency in the left fronto-central areas, while the application of the stimulation leads to an increased efficiency of MWA in the same areas as compared to CONT. In particular, the 20mm checkerboard stimulation shows an increase of integration between the left parieto-occipital and the frontal and central areas, and an increase in efficiency by CONT compared to MWA in the right intra-hemispheric area, and in the connection between the right and the left hemispheres.

MWoA/CONT comparison Again, the CPL do not show differences between the two populations but shows a 12% approximately larger eccentricity of the CONT compared to MWoA in the base. It is interesting to note that in the 20 mm checkerboard stimulation, the MWoA eccentricity in the central left area is larger than the CONT at higher frequencies by an amount close to 10%.

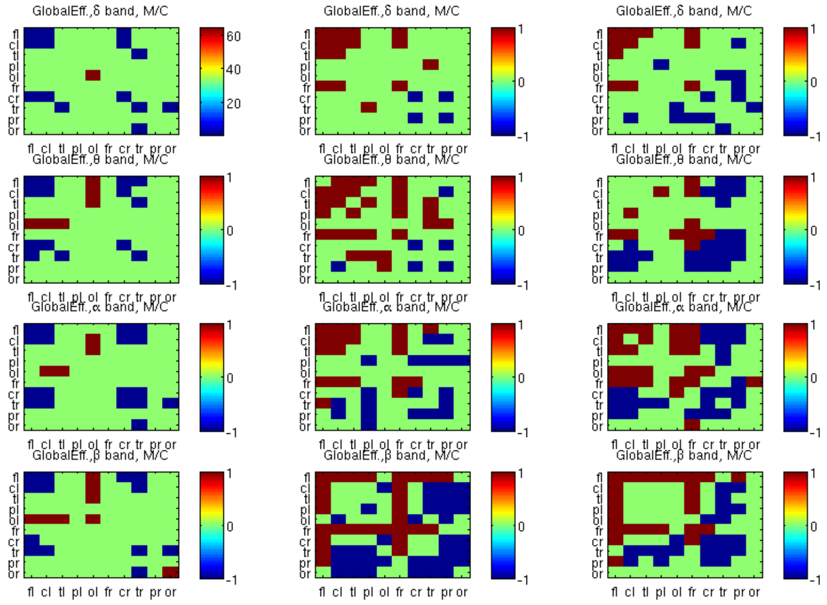

Figure 5.11: MWoA/CONT comparison based on the Global Efficiency. The three columns stems for, respectively, the basal activity, 5 mm and 20 mm stimulations.

The global efficiency analysis shows, beyond the almost total lack of distinction between the two populations in the basal activity (the only difference is the higher efficiency of the left fronto-central areas in CONT, but only of a few percentual points), a different spatial distribution of the efficiency depending on the type of stimulation and of the frequency band.

In both stimulations, the first three bands show a reversal with respect to the basal conditions, as the fronto-central areas efficiency is higher in MWoA; at the same time, the higherer β band efficiency of the frontal area in MWoA to transfer and receive information from all other cortex areas is evident.

Stimulation with 20mm checkerboard shows higher right intra-hemispheric efficiency of CONT and their higher ability to connect the right and left hemisphere between them (figure 5.11).

In conclusion it can be stated that, on the basis of functional integration, the two migraine phenotypes show a reduced eccentricity compared to healthy patients, particularly evident in the left lobe of the central area. This trend is inverted once the stimulation starts, leading MWoA and MWA to an higher eccentricity in the same areas. Regarding the global efficiency, it has been noted that at a basal level the three populations do not differ substantially, but with the arrival of the stimulation the situation changes as a function of population: the MWA make the left parieto-occipital areas more efficient, while the MWoA make the fronto-central areas more efficient compared to CONT in both stimulations.

Segregation measures

MWA/MWoA comparison At basal level, the comparison between the two phenotypes of migraine shows how the left central area of MWoA results 20% more clustered than the MWA. This distinguishing factor should also be considered on the basis of local efficiency, which shows in the same area a larger MWoA efficiency than MWA of about 10%. On the basis of this parallel, it can be stated that MWoA specialize the left central area in order to process the information, being simultaneously clustered and efficient. This basal phenomenon is repeated on all frequency bands and in δ band of all stimulations.

The stimulation arrival leads to two different phenomena: on one hand, MWoA specialize parietal areas in α band (which are *associative* areas, who process and distinguish sensorial stimulation), while MWA specialize the left central areas: this leads to state that the visual stimulation delivery generates a segregation increasing (ie, high specialization) of the MWA left central areas.

No modules number distinction is available for both populations.

MWA/CONT comparison At basal level, there are no peculiar differences between MWA and CONT, if not for the higher clustering of the last compared to the first in the left central areas (about 25%). This feature is not accompanied indeed, as in the previous case, by an equally distinctive local efficiency between the two groups, for which it can not be stated that a group segregate this area more than the other, but only that the MWA have a number of triangles smaller than the CONT in the left central area.

In the 5 mm checkerboard stimulation no particular differences are avident, but the 20 mm checkerboard stimulation shows in α and β bands particular structures that are both clustered and locally efficient in the MWA. It is the entire central areas, and in particular channels F7 and TP7 for the left hemisphere and FT8 and CP6 for the right, which show a deeper mean segregation of 21% compared to CONT.

At the same time, the left parietal areas are highly specialized in CONT compared to MWA, albeit lower than the previous: 13 % approximately.

MWoA/CONT Comparison Little differences between the two groups: basic activity always shows a larger clustering coefficient (approximately 11%) of the CONT compared to MWoA in the right center lobe, which is not matched by a distinctive local efficiency between the two groups.

Only α and β bands, as before, show a larger segregation of MWoA compared to CONT in the central-parietal areas and in the same channels previously seen F7/TP7 and FT8/CP6, in a (combined) percentage 9% approximately.

At the conclusion of the comparison, it can be affirmed that the stimulation with checkerboards (and especially the one with side of 20 mm) generates a reversal of specialization in the central areas and with respect to the central-parietal normal basic activities, which passes from being greater in CONT in basal conditions to be higher in the two phenotypes of migraine during stimulation in the higher frequency bands.

Centrality measures

MWA/MWoA comparison The analysis of the betweeness centrality starts from the Vertex variant (VBC), and clearly shows how, in the comparison between the two phenotypes of migraine, the basal activity presents a strong centrality of the left center areas (on average, 25%) in MWA compared to MWoA in all bands. The stimulation arrival causes, especially the one with 20 mm edge, the parietal-occipital areas to be more central in MWA compared to MWoA of about 35% in the higher frequency bands.

Even the analysis of the Edge variant (EBC) clearly shows an increased centrality of connections between parieto-occipital to almost all other areas of the MWA and of the fronto-central from all the others. Consequently, it is possible to speculate for the posterior areas to have a “bypass” function for the information, that uses these areas to connect otherwise disconnected areas of the cortex, as seen in figure 5.12, in which are shown the “average” networks for the two phenotypes [84].

The parallel between the two complementary measures Z-score/participation provides an interesting result. On the basal activity there are no differences between the two groups, while the visual stimulations show, in the uppermost bands α and β, an higher Z-score in MWA parietal-occipital areas (which can reach the 40%) and a larger participation coefficient in MWoA (of about 27%) in the same areas, particularly in the right hemisphere. According to what we have seen in section 4.4.6, this is indicative of the fact that the parieto-occipital areas of MWA behave essentially as *local hubs* of the network, or as areas facilitating the (already seen) functional segregation of the posterior areas of the cortex.

Of a certain relevance, in the lower bands, is instead the behavior of the channels FCZ-CPZ, which have a large inter-modular Z-score (which does not correspond to a high level of participation) during stimulation with 20 mm checkerboard: it is likely that, at low frequencies, these channels behave simply as *hubs* for the sub-cluster consisting in frontal $\leftrightarrow$ central $\leftrightarrow$ parietal

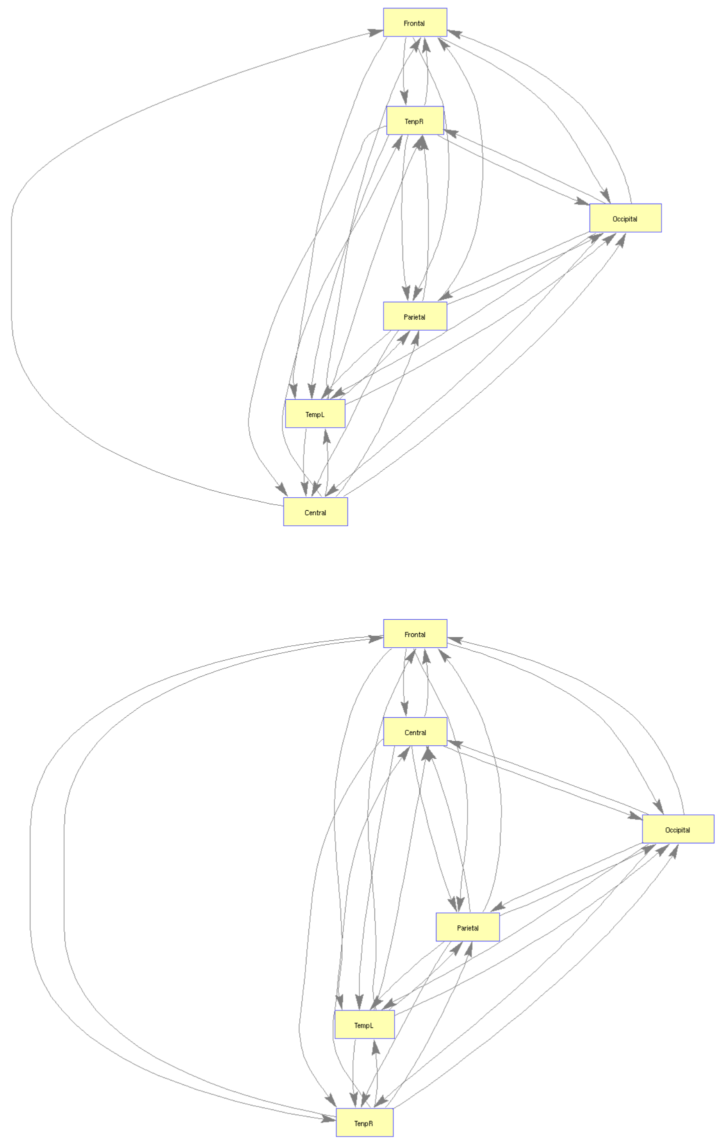

Figure 5.12: network diagrams for MWA (top) and MWoA (bottom) in the stimulation with 5 mm checkerboard in α band. It is evident the by-pass role of the the occipital area in the MWA in the anterior $\rightarrow$ posterior connection which is not symmetric as in MWoA.

areas and between the left and right hemispheres of the central area.

MWA/CONT comparison Beyond a slight distinction (about 4%) in the α and β bands of the left frontal area which, in the basal activity, shows a larger VBC of CONT with respect to MWA, no significant distinction can be made between the two groups.

EBC also shows very little differences both with the increase of the frequency bands and with the stimulation sequence. A single distinction can be made, in β band, on the central-parietal paths of the MWA, which are 12% more central than the CONT.

Finally, also the parallel between the couple of measures Z-score/participation shows no substantial differences between the two groups, if not for a series of channels, that indeed do not show a defined pattern of distinction.

MWoA/CONT comparison Among the two populations there are few signficative differences. One of these is the variation of the VBC in the left frontal area in β band that, if during the basal activity is 10% larger in CONT, during the stimulation is in favor of MWoA of the same quantity. No definite pattern of distinction can be found for the EBC.

Even the pair of indicators Z-score/participation shows few differences between the two groups, and only at the basal activity level: in θ band only it can be noted that CP2, CP1 and CP5 channels have a larger Z-score in MWoA (about 50%) and a larger participation coefficient in CONT (a few percent). Reconsidering this behavior only by reference to the CONT characteristics, it can be stated that these channels act like *local hubs* for MWoA.

Resilience measures

MWA/MWoA comparison A significant difference between the two migraine phenotypes can be seen in the β activity during stimulation with 20 mm checkerboard, which shows an increase of assortativity in MWoA than

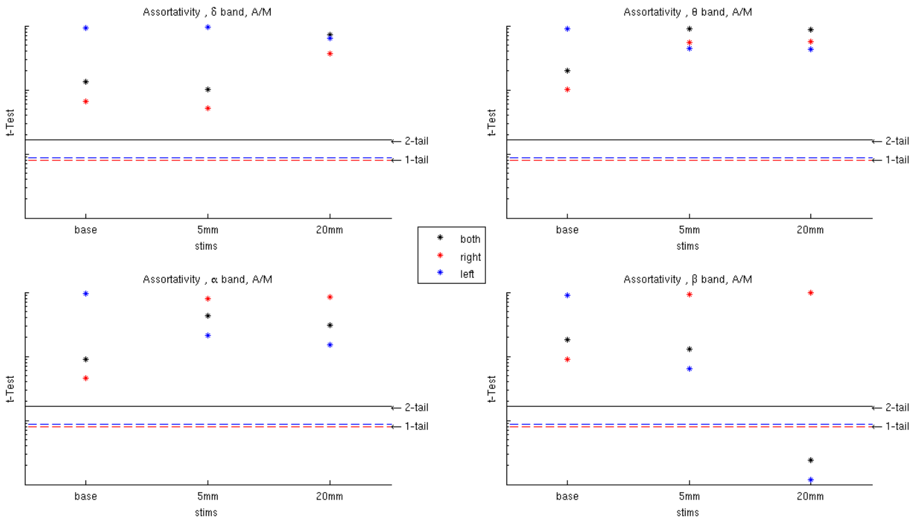

Figure 5.13: assortativity analysis for MWA/MWoA comparison. “Both”,‘right” and “left” refers to the tails of the Gaussian.

the MWA (figure 5.13) even presenting, the latter, a joint-degree distribution whose peack lies on significantly larger values.

Concordantly with the results already seen, the total degree of the left central area is significantly larger in MWA that in MWoA, as are, during the visual stimulations, the parieto-occipital.

Also in 20 mm stimulation a larger number of conenctions in the left temporal area is often present and characterizes the MWoA in comparison to MWA both from the intensity and the numerosity point of view (40% in both cases).

MWA/CONT comparison Even if not showing significant differences in the joint-degree distribution, the comparison between MWA and CONT on the basis the assortativity shows a larger coefficient of MWA compared to CONT in α and β bands in the 20 mm checkerboard stimulation (about 12%).

At the same time, the degree and the strength of the connections of the

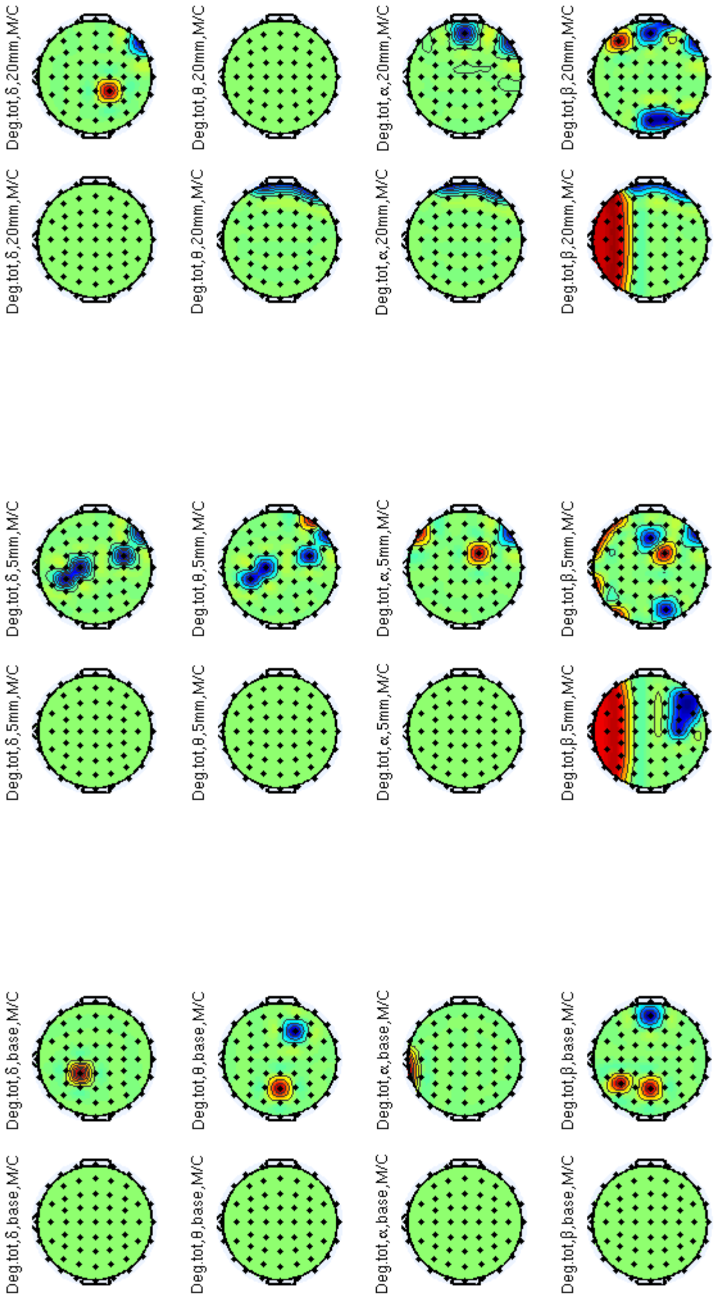

Figure 5.14: analysis of total degree distinction in MWoA/CONT comparison. Each column is formed by two sub-columns: the first indicates areas that in their entirety distinguish the two populations (with the usual convention on the colors), the second indicates locally the possible distinction.

temporal regions (both right and left) is significantly higher in CONT by a factor of 0.6, while at lower frequencies of 20 mm stimulation some channels across the left and right central areas appear to have strong connections (about 20% stronger) in the MWA.

MWoA/CONT comparison No significant differences between the two populations in terms of assortativity, but CONT show a network degree distribution peacked on larger values in MWoA in β band in all stimulations.

In terms of total degree, the distinction between the two groups manifests itself differently depending on the frequency band and on the stimulation. In fact, if in β band both stimulations show an increase in the number of connections of the frontal lobe in the MWoA compared to CONT, in the lower bands the distinction depends on the type of stimulation: in both cases the CONT degree is larger if compared to MWoA of more than 30%, but while in the 5 mm stimulation the distinction affects essentially some channels in the fronto-central areas (F1 and FCZ) and some of the right parietal area (P2, PO8), the 20 mm mainly interests the right temporal areas (figure 5.14).

5.6.3 Conclusion

Few differences exist between the basic connectivity of the two phenotypes of migraine, while CONT always show higher levels of ingoing and outgoing information with respect to MWoA.

The 5 mm checkerboard stimulation is characterized by increased information levels of MWA in central bands, θ and α, associated with a structure vaguely reminiscent a capitol X centered on the Fz channel for the incoming flux; for the outgoing information flow this structure disappears, and only the mentioned channel is still present and distinguishes MWA from CONT and MWoA. In the outer bands, TE levels for MWA are reduced since below those of MWoA and CONT.

The same Fz channel shows a significant difference between MWoA and CONT, since in all bands it shows higher TE levels in CONT, while in the

front area MWoA always reveal higher levels of exchanged information.

Even in the 20 mm checkerboard stimulation it is possible to distinguish between the two migraine phenotypes and CONT basing on the Fz channel behavior and areas in its immediate surroundings: the X-shaped structure seen in the previous stimulation appears again and distinguishes MWoA and MWA from CONT, due to an higher level of outgoing information characterizing the first two with respect to the latter in all bands. Even in the MWA/MWoA comparison the role of the Fz channel is relevant, as the information levels sent from this site are significantly higher in MWA.

As, furthermore, there are no substantial differences between the populations in terms of the overall connection length, the *eccentricity*, a measure of the anisotropy with which each channel (or cortex area) distributes information, distinguishes populations: the low eccentricity of MWA and MWoA compared to CONT appears to be an hallmark of the migraine phenotypes.

At the same time, it can not be stated that MWA are more efficient than MWoA in information distribution, as the global efficiency analysis has shown that stimulation, depending on the population under investigation, makes efficient different areas: the MWoA increase the intra hemispheric efficiency compared to MWA, which in turn increases the parietal-occipital areas efficiency, and this occurs both in relation to the two types of migraine, both compared to CONT, that in the basic activity, in any case, are more efficient. This is no longer true during stimulation, in which the two phenotypes of migraine recover efficiency compared to healthy patients.

Regarding the specialized areas in information processing, it can be steted that MWoA and MWA specialize the same areas in both stimulations: while in the basic activity the central areas in MWoA are already specialized, associating, during stimulation, the parietal areas (which are responsible for the associative processing during stimulation), MWAs seem to specialize central areas "lately" (ie, only during stimulation) with respect to the first. Introducing also the CONT in the comparison, it has been shown that the two

forms of migraine show, concerning channels F7/TP7 and FT8/CP6, typical structures showing high functional segregation if compared to CONT.

The analysis of centrality shows the most important characteristic of the comparison between MWA and MWoA: the lack of bi-directionality in the MWA information flow causes the parietal-occipital lobe to act as a *bypass* to sort information in MWA if compared to CONT and MWoA; such an effect is confirmed by the possible attribution to these areas the ststus of *local hubs* for MWA. For MWoA, on the other hand, the *local hubs* seem to consist of the three channels CP1, CP2 and CP5.

Finally, the analysis of resilience clearly shows how the capacity of recovering of healthy patients is higher than those, in sequence, of MWoA and MWA: the network of the latter, with assortativity and average degree distribution smaller than the others, are potentially less able restore connections if subject to breakdown.

What, maybe, is of paramount importance is the fact that the results, submitted to the neurologist opinion, have shown themselves in complete accordance with the known features of migraine with and without aura, so the used models and procedures can be considered as a fundamental tool to recover more aspects of the clinical assets of such a pathologies [81, 82].

5.7 Evoked potentials by laser painful stimulation

The available EEGs consisted of 61 channels recordings of neuroelectrical cortex activity from both patients experiencing migraine (MIGR, 29 patients) and healthy patients (CONT, 16 subjects). The sampling frequency of the signal was 256 points per second again.

The painful stimulation was delivered by a laser striking the right hand, the power of which was regulated, subject by subject, just above the pain

threshold and *was no longer changed throughout the session.* Before the stimulation arrival, the subject was verbally advised with an hint on the power of the laser pulse. This suggestion could be correct (threshold of pain, $L^{(0)}$), or misleading, inducing in the subject the idea that stimulation would be below the pain threshold ($L^{(-)}$) or definitely much higher than this threshold ($L^{(+)}$).

The recordings consisted, therefore, in two different sections of the same length: one second before the stimulation arrival reporting the *response to verbal suggestion*, and one second after the laser strike, recording the *response to painful stimulation.* For each subject and for each stimulation, from 10 to 15 stimulations were available.

The statistical analysis was conducted by means of the t-Student test (alternating the single tails) for the post-stimulation direct comparisons, with a Bonferroni correction equal to 4, whereas the comparison between the pre and post stimulation in the two populations was conducted using a two-way unbalanced ANOVA with a Tukey Kramer post-hoc correction.

Even in this case, given the high number of available channels, it was decided to study the behavior of the two populations for each channel, so as to improve the spatial resolution in the study of the cortical neuroelectric activity.

5.7.1 Wavelet Analysis

Using a Morlet mother wavelet, each channel was analyzed by means of the of wavelet transform method. The result (corresponding to the channel CP6) is shown in figure 5.15 and is illustrative of almost all channels of the scalp, with the exception of those in the neighborhood of the *vertex.*

An abnormal activity is immediately visible until θ band in MIGR patients which, to some extent, anticipate the reaction to the real painful stimulation. Such a phenomenon, that is absolutely not observed in healthy patients, takes place about a second before the arrival of stimulation and is

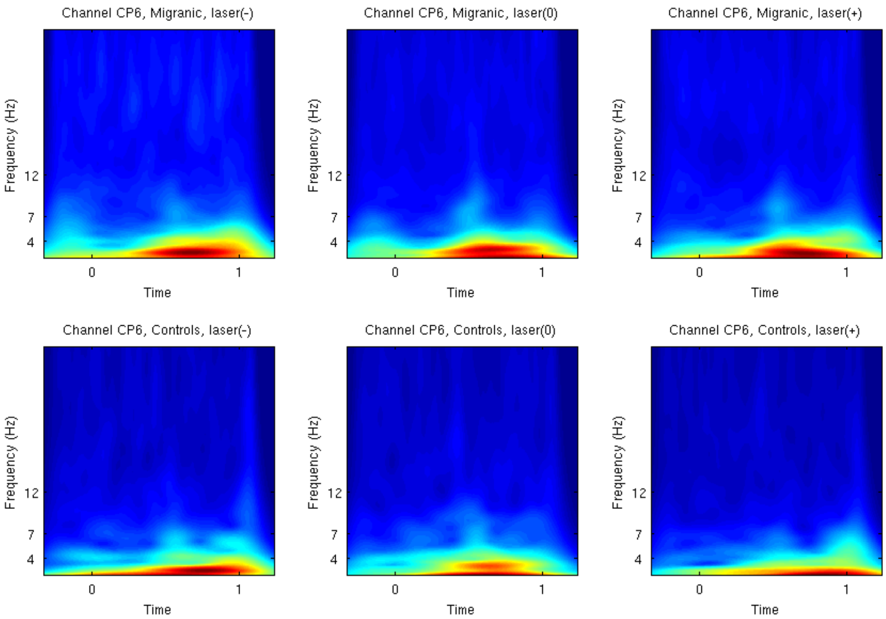

Figure 5.15: wavelet analysis of the response to the verbal and painful stimulations sequence (CP6 channel). The painful stimulation is delivered at the center of the time axis.

independent on the type of alert preceeding it.

Such an "anticipation" has a shape similar to that which, subsequently, will be produced by the painful stimulation, therefore represents, in accordance with the neurologist interpretation, a real response to the suggestion that MIGR offer to the pain stimulation. Its shape is also independent of the suggested intensity and the particular electrode considered.

From the point of view of the statistical treatment of the data, it was first necessary to normalize the wavelet images in both population, as the numerical value of each element was always larger in MIGR than in CONT, leaving no chances for a morphological analysis of the same.

Once data had been normalized so that they varied in the [0,1] interval,

the statistical analysis with the Student's test has showed a statistically significant difference at 0.2 ± 0.03 seconds after the recording started, or about 0.8 seconds before the painful stimulation arrival, reflecting the fact that MIGR somehow anticipate their activity preparing certain areas for the arrival of the real painful stimulation.

5.7.2 Analysis of effective connectivity: Transfer Entropy

By using the procedures already seen in the chapter dedicated to TE, and imposing that the model order m was 1, we firstly investigated the data distribution trend, verifying its parametric behavior (Kolmogorov-Smirnov test); by means of a subsequent post-hoc Tukey-Kramer test, the gaussian behavior of the distribution has been proved, so that we have not only the certainty of dealing with correct statistical tools, but even to rely on the cited equivalence between TE and GC.

The analysis of the TE as a function of *time lag* was conducted, finding that, within 20 samples (equivalent to approximately 80 milliseconds), there was no variation in the TE with the *lag* across cortex areas, which therefore has been imposed equal to 10 samples (figures 5.16 and 5.17). Such an analysis has been confirmed by the CTCC average maximum value, whose value was 37 samples after signals were synchronized.

The result of the analysis has shown (figure 5.18) that during the painful stimulation, in the first three bands, the information levels exchanged across couples of channels was uniformly higher (up to 50%) in MIGR compared to CONT. But while the amount of input information was quite uniform over all channels, the output information presented differences in the spatial distribution: in δ band, for example, in all three stimulations, the amount of output information from the channels of the right frontal areas remained similar in the two populations, while in α band the output response differentiated depending on the stimulation: as the latter was preceded by the notice

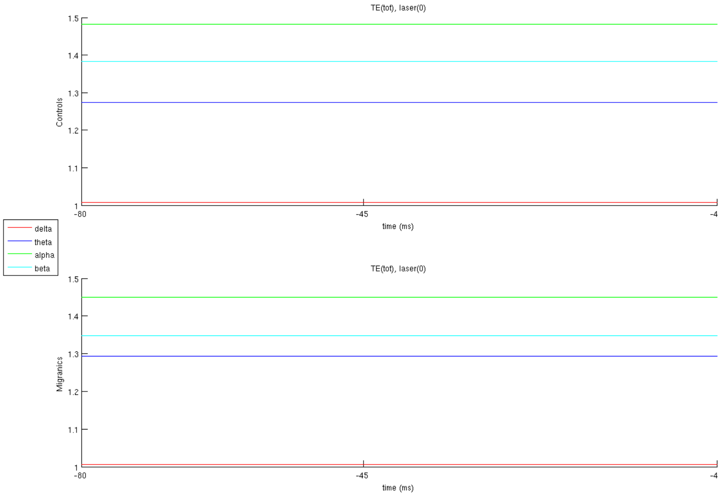

Figure 5.16: trend of the total TE (in + out) with time : it cannot be inferred significant changes within 80 ms prior each considered event. The colors refer to the different frequency bands.

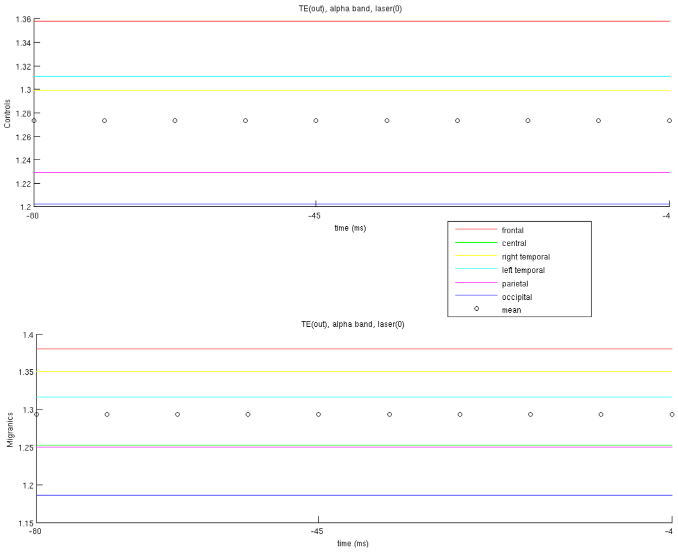

Figure 5.17: total TE trend with time lag in α band in $L^{(0)}$ stimulation: no variations are evident across cortex lobes.

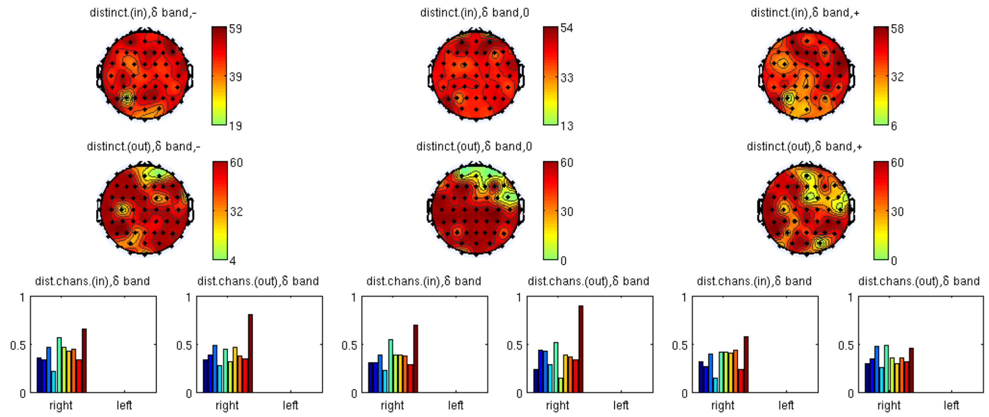

Figure 5.18: TE analysis in δ band. At the top, for each electrode, the number of channels *from* which (or *to* which) there is a statistically relevant difference in the information transferred between the two populations is indicated (warm colors for the channels distinguishing in favor of the MIGR, cool colors for CONT). In the third row the same results as above are reproduced, but on an histogram and as a fraction of the total for each cortex area: in sequence we have front, center, temporal, parietal and occipital areas, respectively in left and right variants; the two indication *left* and *right* refer to the considered tail of the gaussian. In the fourth line we have shown the trends of the mean input and output information levels for the three stimulation and for the two populations.

that it would have been more intense (but that, remember, was always of the same intensity), the areas in which populations are clearly distinguishable expands from the only left fronto-central to the whole scalp.

The β band deserves a separate analysis. In input there is a less shaped difference between the populations, especially in the stimulation declared lower in intensity, $L^{(-)}$, in which there is a channel from the left parietal area (P3) showing, in contrast to the others, an average amount of transmitted information larger in CONT (about 40%) with respect to MIGR. This phenomenon, in fact, is to be considered within a wider context, as it will be clear in a moment.

Regarding the outgoing information, however, the $L^{(-)}$ stimulation is always distinguished from the other two for a poorer differentiation of the response, without presenting a recursive scheme as it does in the other, in which an area of higher levels of exchanged information is particularly evident (figure 5.19) across left and right hemispheres. This aspect will be reconsidered in the comparison between the information networks of the two groups of patients.

As previously mentioned, the different behavior of the P3 channel is to be considered within a wider framework. If we consider, in fact, the only incoming information in the various channels, it can be noted how P3 channel is only one element (the most evident) of two pairs of channels which differ in their behavior with respect to the other.

These pairs are primarily located in the parietal area, one of them in the right hemisphere and the other in the left: these pairs of channels are, in the left hemisphere, CP5 and P3, while in the right one we can find P2 and CP4. Even within these areas, the situation is particular: while the second pair clearly distinguishes MIGR by CONT (ie, it represents an area in which the input information is up to 17% larger for both total quantity of information exchanged and for the number of channels from which it comes), the first looks like an input "information dipole": CP5 infact stands in favor of MIGR, P3 instead distinguishes much less the two populations (at most

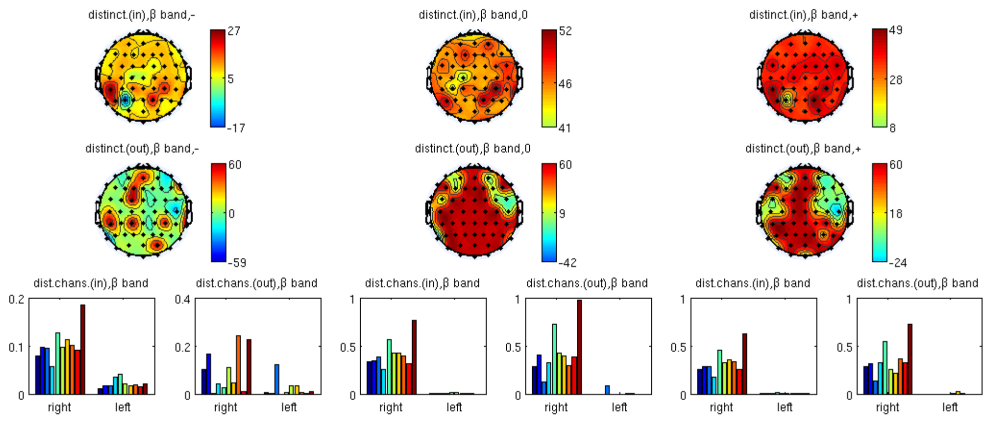

Figure 5.19: TE analysis in β band.

a dozen channels), and always maintains the information levels 15% lower with respect to the surrounding channels, until in β band starts distinguishing about twenty channels in favor of the CONT, as already mentioned.

With the availability of the EEG prior to stimulation, it was possible to make a comparison between the two phases, before and after, by means of a two-way ANOVA test, post-hoc corrected with a Pearson test. This comparison shows how the interested areas in the elaboration of stimulation always remain the same: what changes is simply their extension; in the lower bands (δ and θ) these distinguishing areas tend to shrink of about 10%, while in the higher tend to stretch of the same quantity. However, the phenomenon already seen of the two pairs of channels, CP5-P3 and CP4-P2, is still present, although less evident than in the stimulated case which, of course, amplifies the phenomenon.

5.7.3 Functional Analysis: Synchronization Entropy

The analysis of phase synchronization, conducted by imposing the two weights m and n both equal to 1 (the time series are of the same nature), shows a clear difference in pre- and post-stimulation connectivity, with a further dif-

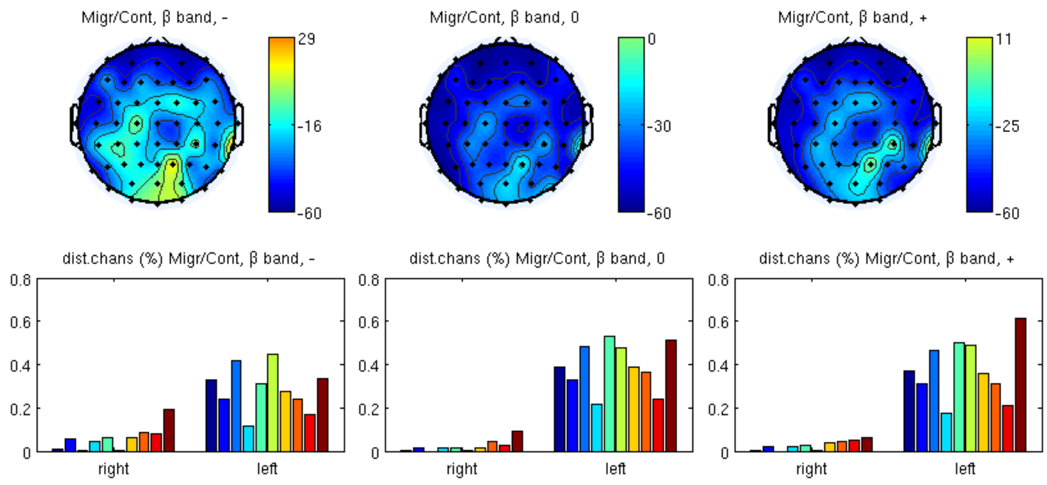

Figure 5.20: analysis of the SE in β band. A “pincer” structure in the parietal-occipital areas in $L^{(-)}$ and $L^{(+)}$ stimulations is evident.

ferentiation on the basis of frequency bands.

In general, in the first three bands (0.5 to 12 Hz), the phase preceding the stimulation shows an higher synchronization level of CONT compared to MIGR (about 30%), with the exception of the two areas around the couples CP5-P3 and CP4-P2, which differs much less the two populations; in particular, when the stimulation $L^{(-)}$ is delivered, most of these areas are activated, and when the suggestion is correct ($L^{(0)}$) or is of the type $L^{(+)}$, in those same areas, the synchronization is smaller, but still in favor of MIGR in intensity.

After stimulation, the latter are uniformly more synchronized compared to CONT, showing a strong synchronization in the areas around the usual channels CP4-P2 and CP5-P3: in this case each of the channels in this areas synchronizes with on average with about 60-70% of the other channels.

However this strong variation of the post-stimulation compared to the pre-stimulation is much more evident when stimulation is preceded by misleading advice; but when the stimulation is preceded by a proper notice, the

cortex synchronizes virtually the same areas that were prepared to recipe the painful impulse in the pre-stimulation, simply increasing the synchronization probability. Many of these features will come back when the response of the information network to stimulation will be discussed.

The β band, however, shows a milder difference between pre- and post-stimulation: the only areas in which this step increases the synchronization of MIGR are the ones surroundings the before mentioned CP4-P2 and CP5-P3 channels, while all the remaining areas are always more synchronized in CONT (figure 5.20).

In any case, even in this band the difference between the stimulation preceded by wrong suggestion produces large effects on synchronization than if preceded by a proper suggestion.

Being such an obvious difference between the pre and post stimulation and between the two populations, in addition to the usual analysis with the t Student test, a multivariate two-way ANOVA test was also performed, considering the two populations as group variables and pre- and post- stimulation as factors, to study the correlation and the possible interaction (figure 5.21).

In this case, all stimulations ($L^{(-)}$, $L^{(0)}$ and $L^{(+)}$) show that the transition from one population to the other (indipendentently on the type of stimulation) activates the usual areas around CP4-P2 and CP5-P3, and other two sites in the front area: the one around FC3 and the one around F4, which are much more synchronized in MIGR. The rest of the scalp is constantly more synchronized in CONT.

The transition, however, from the pre to the post stimulation phase shows a uniform increase in synchronization activity of $L^{(-)}$ and $L^{(+)}$ stimulations in all bands, while in $L^{(0)}$ an increased activity in the phase preceeding the stimulation is evident.

The analysis of the interaction between the two effects (groups $\times$ factors) does show an uniform (ie, over the whole scalp) correlation between them,

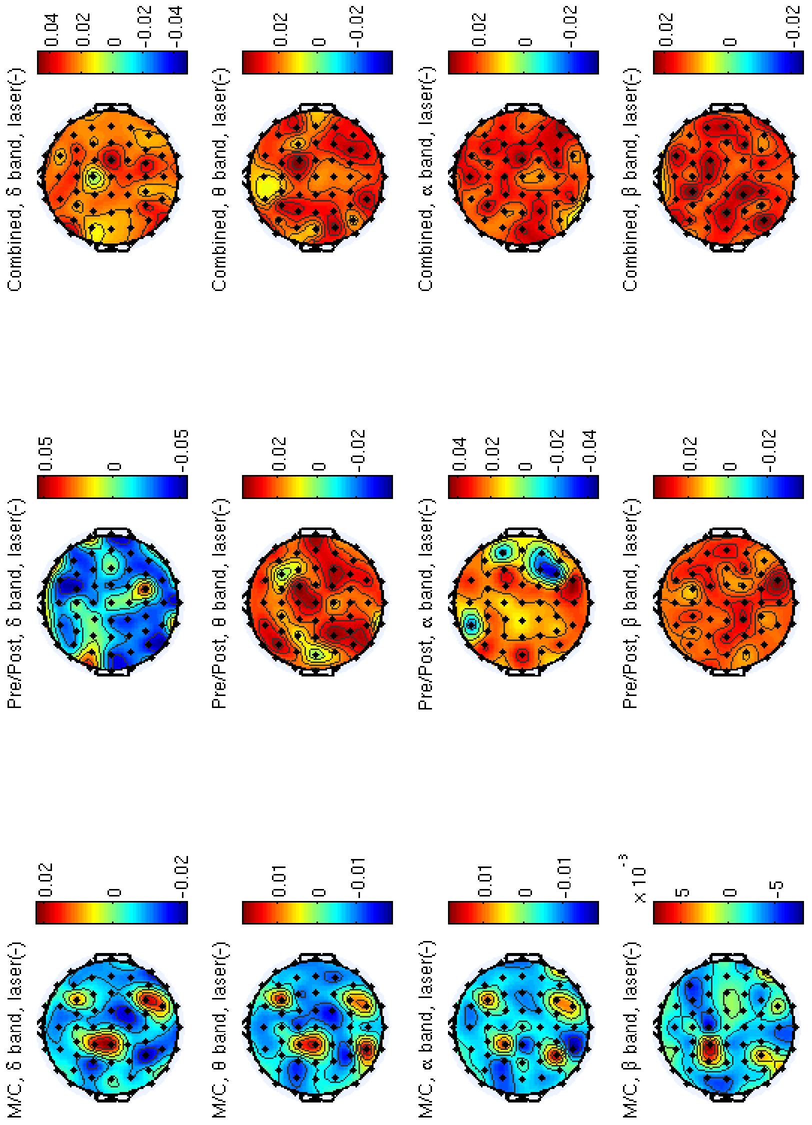

Figure 5.21: Two-way ANOVA for $L^{(-)}$ stimulation.

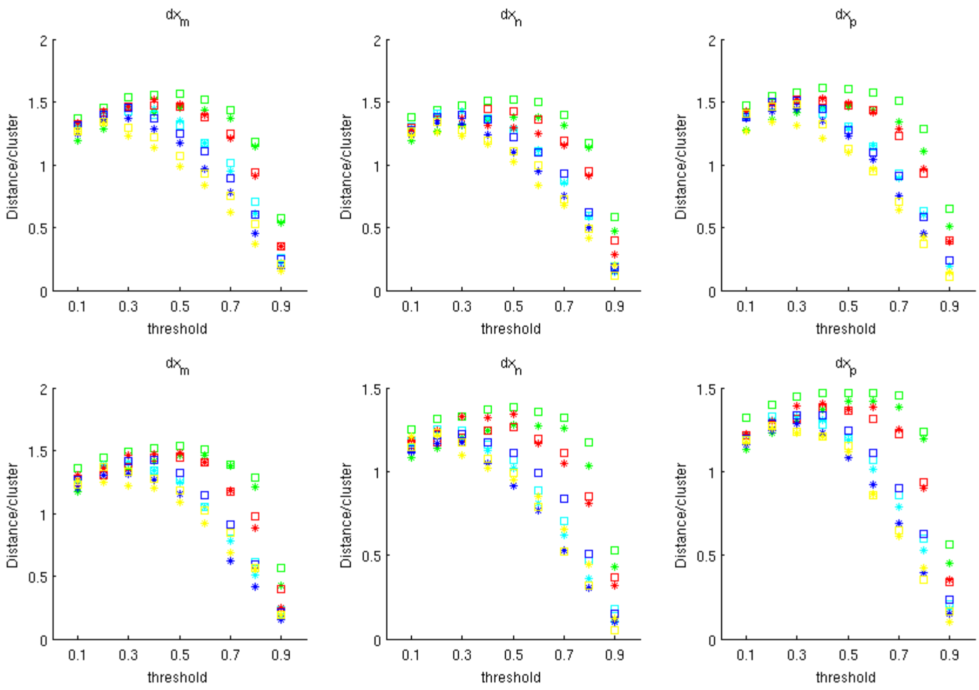

Figure 5.22: trend of the mean distance between nodes in the two populations and in the three stimulations as a function of the threshold. Each point indicates the average distance of a cluster (considered as the area of the cortex) compared to all other in α band. At the top the MIGR, at the bottom the CONT.

even not showing a defined recurrency pattern.

5.7.4 Brain Network Analysis

The choice of the threshold to be applied to connection matrices has been made considering the behavior of different quantities with the threshold the same. Those who did not show an ascending or descending monotonic trend (such as degree, clustering, efficiency and so on) showed a maximum at the adaptive threshold of 60% (figure 5.22): this means that any element of the activation matrix that is located below this value will be null, and the link

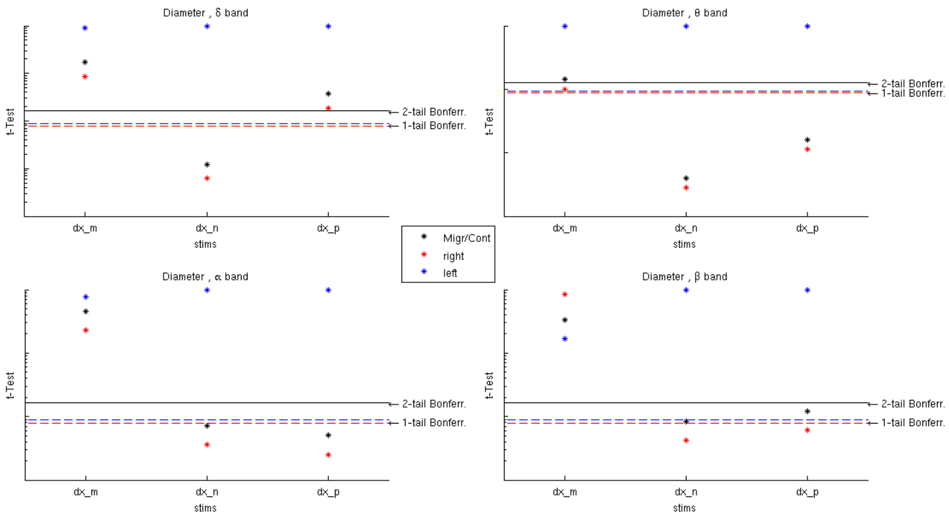

Figure 5.23: comparison between MIGR and CONT on the basis of the average characteristic length of the links in networks.

between the relative nodes will be removed from the network.

Integration measures The analysis of Characteristic Path Length (CPL) shows a larger average length in MIGR connections (about 30%) compared to CONT (figure 5.23), as well as radius and diameter, pointing out the fact that information, in order to be delivered from a node to another, has to follow longer paths, making MIGR for sure less integrated than CONT. Moreover, the analysis of *eccentricity* (a measure of the isotropy in information distribution among all possible paths in the network) shows that, in the surroundings of the structure consisting of CP5-P3 and CP4-P2 channels, a larger eccentricity in MIGR is present, reaching a difference of about 25-27% (figure 5.24). This phenomenon is evident in all frequency bands and in all stimulations.

Particularly interesting is the analysis of global efficiency, showing that, regardless of the frequency bands and the stimulation, the two populations specialize different cortical areas: the MIGR specialize the left area (parti-

cularly the frontal ones) while CONT specialize the whole right area. But while the latter are able to let the two hemispheres to communicate between them, the former do not show as much efficiency in inter-communication (figure 5.25).

This effect is particularly evident when analyzing the $L^{(0)}$ stimulation, while in the other two this effect is less evident.

In the analysis of the different behavior between pre-stimulation and post-stimulation, the integration does not show fundamental differences between the first and the second phase, if not for the fact that the areas with larger global efficiency reduce their estension in the passage through.

Segregation measures The analysis of the clustering coefficient between shows a widespread superiority in the number of *triangles* in MIGR compared to CONT (in percentage, from 29% of the base to 22% of the stimuli), which however show a larger coefficient with respet to MIGR especially in the higher bands and across the two hemispheres, particularly in the posterior lobe of the cortex, at a rate of 6-7% approximately. Furthermore, depending on the stimulation, their extension changes in a sensible manner, extending of about 5% as the suggestion was preventing more intense stimulation. In any case, the extension of these areas is reduced with the frequency bands and the right hemisphere is much more interested than the left.

The simultaneous analysis of the local efficiency shows that, especially in the uppermost band and in those same areas, MIGR are more efficient than CONT of about 10%, while the latter are 15% more efficient in the areas across the two hemispheres, mainly in the $L^{(+)}$ stimulation.

The combined effect of this analysis seems to suggest that MIGR, during stimulations, specialize areas at the right and left side of the longitudinal midline of the cortex, but without showing the same efficiency of the CONT across that line, generating a reduced ability of intercommunication between the two hemispheres.

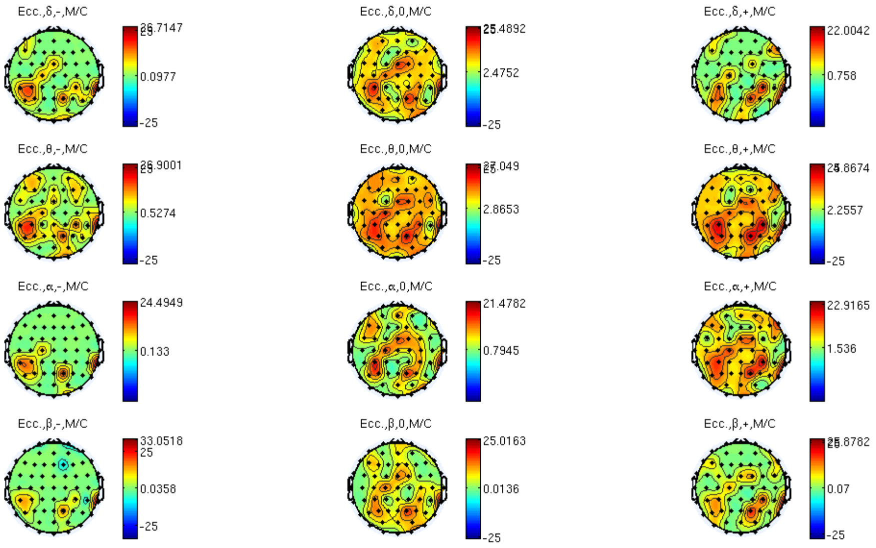

Figure 5.24: trend of the distinction of the CPL "eccentricity" variant on the scalp.

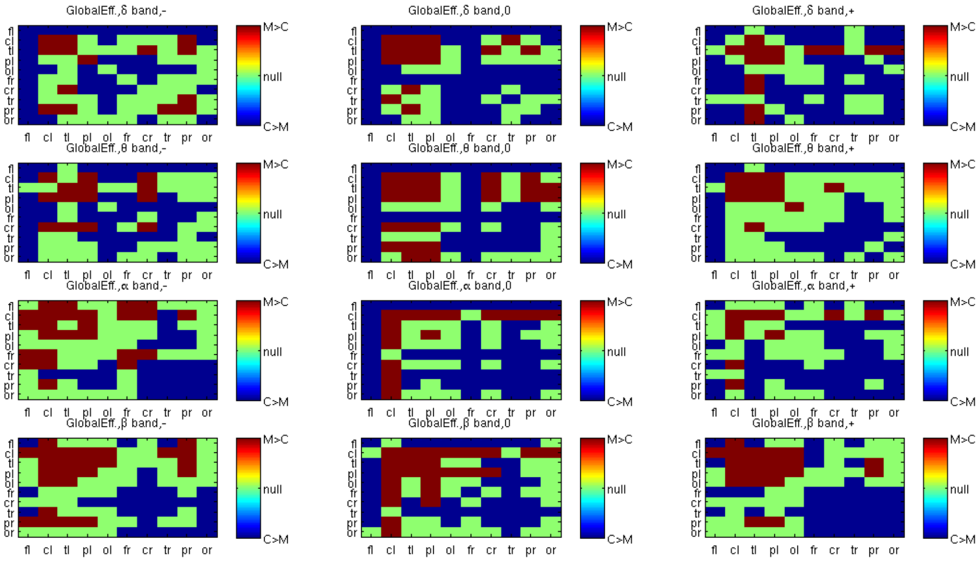

Figure 5.25: trend of the distinction of the global efficiency in cortical areas.

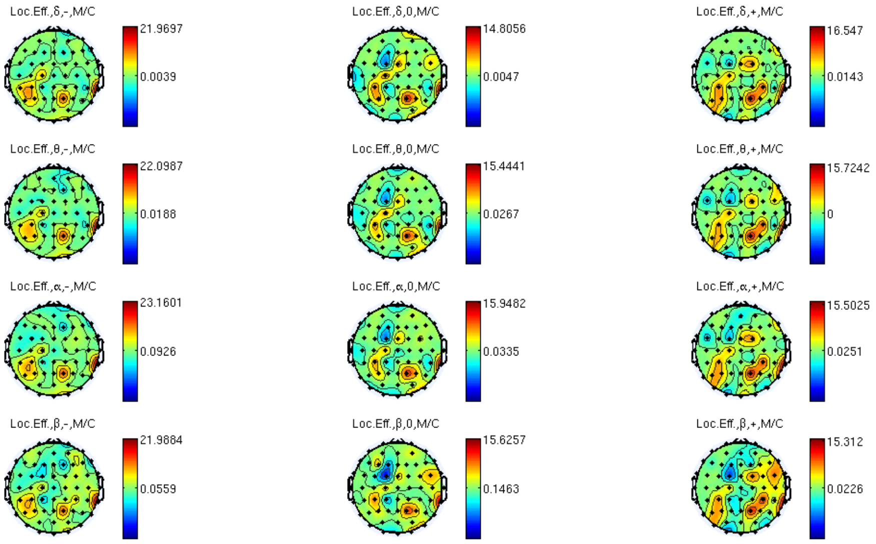

Figure 5.26: trends of the local efficiency distinction on the scalp.

Very interesting is the comparison between the pre-and post-stimulation in relation to the segregation analysis. Basically during $L^{(0)}$ and $L^{(+)}$ stimulation there is no difference between the two phases (ie, the structured areas from verbal stimulation are those actually used to process the painful stimulation), while in $L^{(-)}$ is evident how the cortical structured areas before the stimulation (specialized or not) just increase their size, without altering their shape, of a dozen percentual points.

Centrality measures The analysis the Vertex betweeness does not show significant results, but only a few channels of the right central area presenting a larger coefficient in CONT (about 20%) and in the uppermost band, but that do not follow an unit pattern. The same is true for EBC.

The centrality analysis highlights, particularly in $L^{(0)}$, a higher Z-score of the left central area in MIGR (about 9%), while in the right front areas

the CONT show an increased centrality by approximately 25%. The same occurs in the uppermost band of the other stimulations.

The simultaneous analysis of the participation coefficient shows that in the same areas and the same bands CONT are more *participatory* of MIGR of about 20%. This effect indicates that the CONT have an higher tendency to centralize the left central area with respect to MIGR using it to sort the information coming from other cortex areas.

At the same time, the MIGR always have a larger participation coefficient of in the frontal areas, particularly those on the right lobe, with percentages ranging from 12 to 19% (figure 5.27).

Even in this case, the difference between the phase of verbal stimulation and that of painful stimulation is only in $L^{(-)}$, in which, once again, the areas tending to segregation and cenrtality simply expand themselves, while in the other two stimulations there is no difference between the first and the second phase.

Resilience measures The analysis of the assortativity coefficient and of the network degree distribution indicates, especially during $L^{(0)}$ and $L^{(+)}$ stimulations, that CONT shows an increased ability to restructure the pattern followed by information (figure 5.29). Even more interesting is the fact that this happens regardless of the verbal stimulation that precedes the painful strike. It is therefore a factor independent of the particular response of healty patients.

5.7.5 Conclusion

The analysis *double stimulation* sequence (verbal + painful) showed many interesting differences in the two populations' behavior. First of all, it showed how MIGR anticipate the activity in θ band before the arrival of the painful stimulation in the largest part of the cortex regardless of the warning on its intensity (held constant).

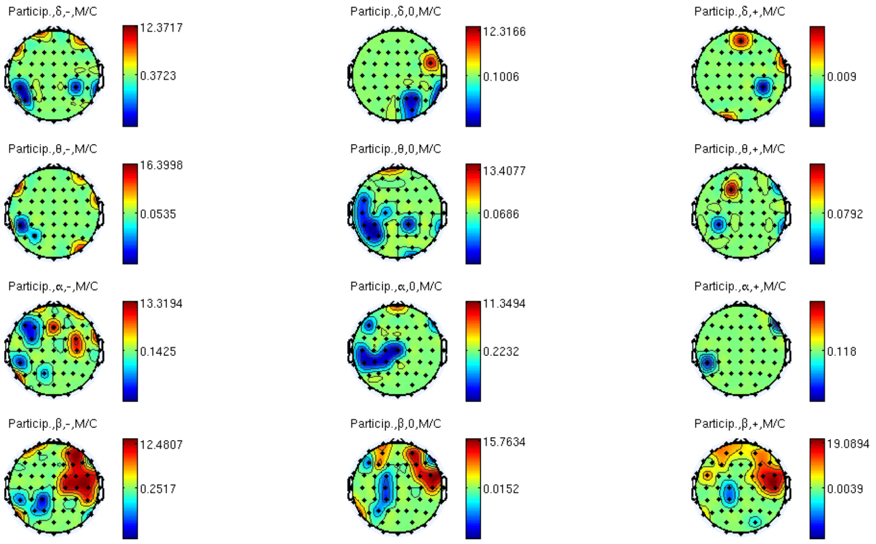

Figure 5.27: distinction of the participation coefficient on the scalp among MIGR and CONT.

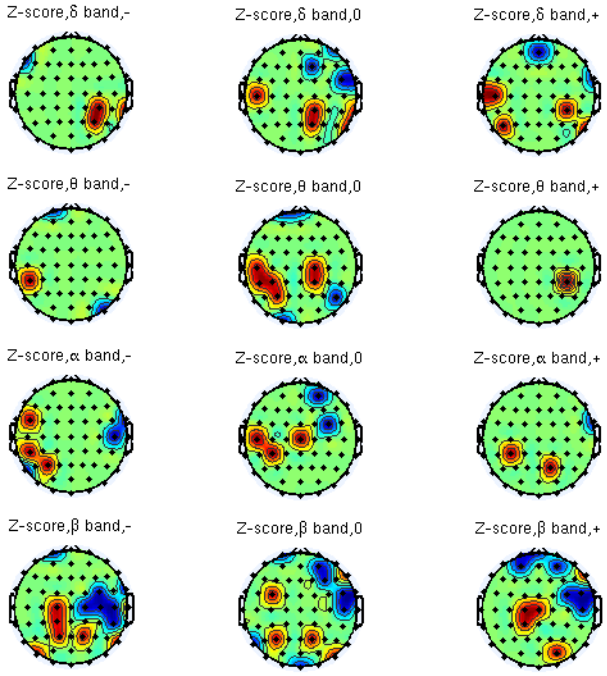

Figure 5.28: distinction of the Z-score coefficient on the scalp among MIGR and CONT.

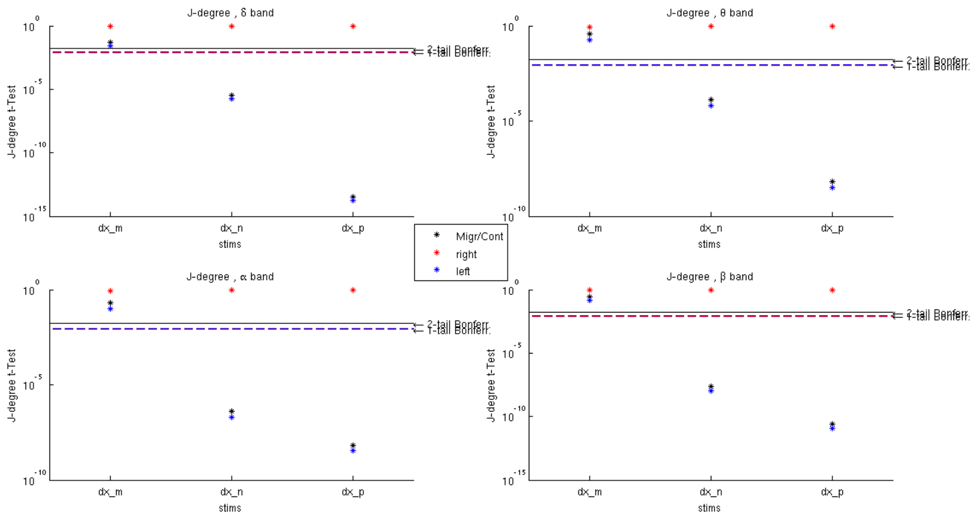

Figure 5.29: differences in the Joint Degree distribution in the basal activity and during stimulation.

The analysis of effective connectivity showed that the amount of information exchanged between the various cortex areas is different in input and output and depends on the type of warning preceding the painful stimulation: in the first three frequency bands, the TE level is significantly higher in MIGR, but while the distinction in input is almost uniform over the entire scalp, the outgoing TE levels differ depending on the stimulation and on the bands; in any case, the distinction areas seem to be expanding as the notice was advising for highest intensity stimulation.

In β band, however, it appears to be a channel (P3) showing, in contrast with the generally higher TE levels in MIGR compared to CONT, a similar behavior for the incoming information in both populations. In output, however, the distinction between the two populations covers a wide area across the two hemispheres, the extent of which increases as the migranic patient is suggested to be hit with a more painful stimulation.

The comparison between the pre-and post-stimulation has also highlighted as areas that verbal stimulation has somehow "prepared" are then actually used in the processing of the painful stimulus itself. The only difference between the two phases consists in the extension of these areas, that in the transition between the two phases tend to either retire (δ and θ bands) or expand (α and β).

In this context, P3, CP5, CP4 and P2 channels form a structure in the form of a "pincer" that from the parietal-occipital area goes up to the central one, and mark a significant difference in the functional connectivity of the two groups: such a structure, altough well-known in neurological literature, in which the SE levels are higher in MIGR than in CONT, is always similar in shape to itself in all bands, in all stimulations and in all comparisons between the two populations.

The analysis of the characteristics of the averaged networks of the two populations show a wider integration of CONT compared with MIGR, having the last a longer average length of the connections. However, it is also evident that the two populations make efficient different areas, regardless of the type of stimulation: the MIGR acts on the left areas of the cortex, the CONT acts on the right and the inter-hemispheric connections. These areas tend to shrink themselves in the transition from the first (verbal stimulation) to the second phase (laser stimulation).

At the same time, the reduced ability of MIGR to specialize areas across the median line of the cortex (the one separating the right and the left hemisphere), which is a peculiar feature of the CONT, suggests a reduced ability of communication between the two hemispheres of the MIGR compared to healthy patients. In this context, the transition from verbal to painful stimulation highlights the fact that, while in the stimulations $L^{(0)}$ and $L^{(+)}$ the suggestion-structurated areas are the ones the populations actually used during painful stimulation, in the $L^{(-)}$ such areas just increase their extension.

The CONT, for their part, show a marked tendency to centralize, in order to sort information, the left central area with respect to MIGR. Once again,

finally, it is clear from the analysis of the resilience that the CONT have a higher resilience than the MIGR, regardless of verbal or painful stimulation.

We want to emphasize, here, that the behavior of MIGR patients in the two types of studies considered (SVEPs and laser) is in no way comparable, since the two forms of stimulation are completely different: the first is related to the *striated associative* areas, while the second is related to the *brainstem* activity.

Even in this case, the neurologist opinion is that most of the features pointed out in this work are consistent with the known ones from medical literature, confirming once again that the model and the results of the application of it on real biological data can fournish more and important new insights of neuroelectrical dynamics.

Conclusions

At the end of this work, it can be affirmed that the performances and the results of the different indicators of the brain activity/connectivity are openly a useful tool to investigate celebral dynamics, as its results have always been confirmed to be correct on the basis of the actual neurological knowledge.

Moreover, they have stresed the fact that, in addiction to the already known distinguishing features of the different phenotypes of migraine, they are also capable of pointing out particular new aspects of connectivity and specialization of cortical lobes and, when it is possible, of smaller scalp areas, that is more restricted neural clusters.

The improvement of computing performances and of spatial resolution in EEG recordings will be capable of obtaining new tools (as the *information storage*, the still-under-evaluation evolution of transfer entropy) for the connectivity investigation and more powerful features of brain networking, such as the Rentian Scaling, so that to have a more complete picture of the brain dynamics.

Bibliography

[1] C.E. Shannon, *A mathematical theory of communication*, Bell System Tech. J. 27 (1948) 379-423.

[2] T. Schreiber, *Measuring information transfer*, Physical review letters, vol. 85, no. 2, pp. 461-464, 2000.

[3] C.E. Shannon, *The bandwagon*, IRE Transactions on Information Theory, vol. 2, no. 3, p. 3, 1956.

[4] Lee, Joon et al. *Transfer Entropy Estimation and Directional Coupling Change Detection in Biomedical Time Series*, BioMedical Engineering OnLine 11.1 (2012): 19. ©2012 BioMed Central Ltd

[5] M. Lindner, R. Vicente, V. Priesemann, M. Wibral, *Information flow in time series data with transfer entropy*, BMC Neuroscience 2011, 12:119 doi:10.1186/1471-2202-12-119

[6] B.W. Silverman, *Circulation*, Volume 26 of Monographs on Statistics and Applied Probability. Chapman and Hall.

[7] W. Zucchini, *Applied Smoothing Techniques*, 1st Edition, October 2003, McGraw-Hill

[8] T. Schreiber, Phys. Rev. Lett. 85, 461 (2000).

[9] A. Kaiser, T. Schreiber, Physica D, **166**, 43 (2002).

[10] Rosenblum, Pikovsky, Kurths, Schafer, Tass: *Phase synchronization: from theory to data analysis*, Handbook of Biological Physics, Elsevier

Science, Series Editor A.J. Hoff, Vol. 4, Neuro-informatics, Editors: F. Moss and S. Gielen, Chapter 9, pp. 279-321, 2001.

[11] D. Gabor, J. IEE London **93**, 429-457 (1946).

[12] M. Smith and R. Mersereau, *Introduction to Digital Signal Processing. A Computer Laboratory Textbook* (Wiley, New York, 1992)

[13] L. Rabiner and B. Gold, *Theory and Application of Digital Signal Processing* (Prentice-Hall, Englewood Cliffs, NJ, 1975).

[14] P. Panter, *Modulation, Noise, and Spectral Analysis* (McGraw-Hill, New York, 1965).

[15] B. Boashash, Proc. of the IEEE **80**, 520-568 (1992).

[16] T. Schurmann 2004 *Bias analysis in entropy estimation*, J. Phys. A: Math. Gen. **37** L295âL301.

[17] G. Strang, T. Nguyen, "Wavelets and Filter Banks", Wellesley-Cambridge Press, 1996.

[18] J. Lewalle, M. Farge, K. Shneider, "Wavelet Transforms", Academic Press, New York, 1980

[19] C.L. Liu, "A Tutorial of the Wavelet Transform". February 23, 2010.

[20] W. Wu, "Extracting Signal frequency information in time/frequency domain by means of continuous wavelet transform", International Conference on Control, Automation and Systems 2007.

[21] P.M. Bentley and J.T.E. McDonnel, "Wavelet transforms: an introduction", in ELECTRONICS B COMMUNICATION ENGINEERING JOURNAL, AUGUST 1994.

[22] A. Cohen, R. Ryan, "Wavelets and Multiscale Signal Processing", Chapman and Hall, London, 1995.

[23] S. Mallat, "A Wavelet Tour of Signal Processing", Academic Press, New York, 1998.

[24] C.W.J. Granger, "Investigating causal relations by econometric models and cross-spectral methods", Econometrica, 37(3):424-38, July 1969.

[25] R.Q. Quiroga, J. Arnhold, P. Grassberger, "Learning driver-response relationships from synchronization patterns", Phys. Rev. E, 61(5):5142-5148, May 2000.

[26] M.G. Rosenblum and A.S. Pikovsky, "Detecting direction of coupling in interacting oscillators", Phys. Rev. E, 64(4):045202, Sep 2001.

[27] C. Schafer, M.G. Rosenblum, H.H. Abel, J. Kurths "Synchronization in the human cardiorespiratory system", Phys Rev E Stat Phys Plasmas Fluids Relat Interdiscip Topics, 60(1):857-870, Jul 1999. 2.3.2.

[28] S. Akselrod, D. Gordon, F.A. Ubel, D.C. Shannon, A.C. Berger, R.J. Cohen, " Power spectrum analysis of heart rate fluctuation: a quantitative probe of beat-to-beat cardiovascular control", Science, 213(4504):220-222, Jul 1981.

[29] P. Tass, M.G. Rosenblum, J. Weule, J. Kurths, A. Pikovsky, J. Volkmann, A. Schnitzler, H.J.Freund, "Detection of n:m phase locking from noisy data: Application to magnetoencephalography. Phys. Rev. Lett., 81(15):3291-3294, 1998.

[30] N. Ancona, D. Marinazzo, S. Stramaglia, "Radial basis function approaches to nonlinear granger causality of time series", Phys Rev E 2004;70:056221.

[31] Y. Chen et al., Phys. Lett. A 324, 26 (2004).

[32] J.D. Farmer and J.J. Sidorowich, Phys. Rev. Lett. 59, 845 (1987).

[33] C.R.Rao, S.K. Mitra, "Generalized Inverse of Matrices and Its Applications", John Wiley, New York, 1971.

[34] D. Marinazzo, M. Pellicoro, and S. Stramaglia, “Kernel method for non-linear Granger causality”, Phys. Rev. Lett. 100, 144103 (2008).

[35] T. Poggio, F. Girosi, Science 247, 978 (1990).

[36] J. Hutchinson, “A Radial Basis Function Approach to Financial Time Series Analysis”, Ph.D. Thesis, Massachusetts Institute of Technology, Department of Electrical Engineering and Computer Science (1994).

[37] J. Geweke “Measurement of linear dependence and feedback between multiple time series”, J Am Stat Assoc 1982;77(378):304-13.

[38] L. Barnett, A.B. Barnett, A.K. Seth, “Granger causality and transfer entropy are equivalent for Gaussian variables”, PACS numbers: 87.10.Mn, 87.19.L, 87.19.lj, 87.19.lo, 89.70.Cf, 2009.

[39] A.K. Seth, “Granger causal connectivity analysis”, Journal of Neuroscience Methods 186 (2010) 262-273

[40] H. Akaike, “A new look at the statistical model identification”, IEEE Trans Autom Control 1974;19:716-23.

[41] G. Schwartz, “Estimating the dimension of a model”, Ann Stat 1978;5(2):461-4.

[42] A. Papoulis and S. Pillai, “Probability, random variables, and stochastic processes”, McGraw-Hill, New York, NY, 2002, 4th edition.

[43] R.A. Horn and C.R. Johnson, “Matrix Analysis”, Cambridge University Press, 1985.

[44] M. Rubinov, O. Sporns, “Complex Network Measures of Brain Connectivity: Uses and Interpretations”, NeuroImage 52 (2010). 1059-1069.

[45] K.J. Friston, “Functional and effective connectivity in neuroimaging: a synthesis”, Hum. Brain Mapp., 2, 56-78, 1994.

[46] B. Horwitz, “The elusice concept of brain connectivity”, NeuroImage 19, 466-470, 2003.

[47] C.T. Butts, "Revisiting the concept of network analysis", Science 325, 414-416, 2009.

[48] J. Wang, L. Wang, Y. Zang, H. Yang, Q. Gong, Z. Chen, C. Zhu, Y. He, "Parcellation-dependent small-world brain functional networks in children with attention deficit/Hyperactivity disorder", Hum.Brain Mapp. 30, 1511-1523, 2009.

[49] C.J. Honey, O. Sporns, L. Cammoun, X. Gigandet, J.P. Thiran, R. Meruli, P. Hagmann, "Predicting human resting-state functional connectivity from structural connectivity", Proc.Natl.Acad.Sci. U.S.A. 106, 2035-2040, 2009.

[50] D. Zhou, W.K. Thompson, G. Siegle, "Matlab toolbox for functional connectivity", NeuroImage 2009 Oct 1; 47(4): 1590-1607. Epub jun. 2009.

[51] K.J. Friston, L. Harrison, W. Penny, "Dynamic Causal Modelling", NeuroImage 19, 1273-1302, 2003.

[52] J. Saramaki, M. Kivela, J.P. Onnela, K. Kaski, J. Kertesz, "Generalizations of the clustering coefficient to weighted complex networks", Phys.Rev.,E Stat. Nonlinear Soft Matter Phys. 75, 027105, 2007.

[53] C.J. Honey, R. Kotter, M. Breakspear, O. Sporns, "Network structure of celebral cortex shapes functional connectivity on multiple time scales", Proc.Natl.Acad.Sci. U.S.A. 104, 10240-10245, 2007.

[54] S. Maslov, K. Sneppen, "Specificity and stability in topology of protein networks", Science 296, 910-913, 2002.

[55] D.J. Watts, S.H. Strogatz, "Collective dynamics of small-world networks", Nature 393, 440-442, 1998.

[56] M.E.J. Newmann, "The structure and the function of complex Networks", SIAM Rev.45, 167-256, 2003.

[57] M. Grivan, M.E.J. Newmann, "Community structure in Social and Biological Networks", Proc.Natl.Acad.Sci. U.S.A. 99, 7821-7826, 2002.

[58] M.E.J. Newmann, "Analysis of weighted networks", Phys.Rev.,E Stat. Nonlinear Soft Matter Phys. 70, 056131, 2004.

[59] L. Danon, A. Diaz-Guilera, J. Duch, A. Arenas, "Comparing community structure identification", J.Stat.Mech. 2005, P09008.

[60] M.E.J. Newmann, "Modularity and community structure in networks", Proc.Natl.Acad.Sci. U.S.A. 103, 8577-8582, 2006

[61] V.D. Blondel, J.-L. Guillaume, R. Lambiotte, E. Lefebvre, "Fast unfolding of communities in large networks", J.Stat.Mech. 2008, P10008.

[62] G. Palla, I. Derenyi, I. Farkas, T. Vicsek, "Uncovering the overlapping community of structure of complex networks in nature and society", Nature 435, 814-818, 2005.

[63] V. Latora, M. Marchiori, "Efficient Behavior of small-world networks", Phys.Rev.Lett. 87, 198701, 2001.

[64] S. Archard, E. Bullmore, "Efficiency and cost of economical brain functional networks", PLoS Comput.Biol. 3, e17, 2007.

[65] D.S. Bassett, E.T. Bullmore, "Small-world brain networks", Neuroscientist 12, 512-523, 2006.

[66] M.D. Humphries, K. Gurnay, "Network 'small-world-ness', a quantitative method for determining Canonical network equivalence", PLoS ONE 3, e0002051, 2008.

[67] O. Sporns, R. Kotter, "Motifs in brain ntworks", PLoS Biol. 2, e369, 2004.

[68] R. Gumiera, L.A.M. Amaral, "Cartography of Complex networks: modules and universal roles", J.Stat.Mech. 2005, P02001.

[69] U. Brandes, "A faster algorithm for betweeness centrality", J.Math.Sociol. 25, 163-177, 2001.

[70] S. Kintali, "Betweeness centrality: algorithms and lower bounds", Georgia Tech, College of Computing Technical Report GT-CS-09-10, 2008.

[71] J. Alstott, M. Breakspear, P. Hagmann, L. Cammoun, O. Sporns, "Modelling the impact of lesions in the human brain", PLoS Comput. Biol. 5, e1000408, 2009.

[72] A.L. Barbasi, R. Albert, "Emergence of scaling in random networks", Science 286, 509-512, 1999.

[73] R. Pastor-Satorras, A. Vasquez, A. Vespignani, "Dynamical and correlation properties of the internet", Phys.Rev.Lett. 87, 258701, 2001.

[74] M.R. de Feo, O. MMWArelli, "Testo-Atlante di Elettroencefalografia Clinica" (2001, Marrapese Ed.- Roma).

[75] Headache Classification Committee. The International Classification of Headache Disorders II. Cephalalgia 2004; 24: 24â136.

[76] L. Angelini, M. de Tommaso, M. Guido et al., "Steady-state visual evoked potentials and phase synchronization in migraine patients". Phys Rev Lett 2004; 93: 038103.

[77] M. de Tommaso, V. Sciruicchio, M. Guido et al., "Steady-state visual-evoked potentials in headache: Diagnostic value in migraine and tension-type headache patients". Cephalalgia 1999; 19: 23-26.

[78] D. Genco, M. de Tommaso, A.M. Prudenzano et al., "EEG features in juvenile migraine: Topographic analysis of spontaneous and visual evoked brain electrical activity: A comparison with adult migraine". Cephalalgia 1994; 14: 41-46.

[79] R.H. Simon, A.W. Zimmerman, A. Tasman et al., "Spectral analysis of photic stimulation in migraine". Electroencephalogr Clin Neurophysiol 1982; 53: 270-276.

[80] K. Shibata, K. Yamane, Y. Nishimura et al., "Spatial frequency differentially affects habituation in migraineurs: A steady-state visual-evoked potential study". Doc Ophthalmol 2011; 123: 65-73.

[81] C. Ayata, "Cortical Spreading Depression Triggers Migraine Attack", Pro Headache 2010; 4: 725-730.

[82] N. Hadjikhani, M. Sanchez Del Rio, O. Wu, D. Schwartz, D. Bakker, B. Fischl, et al. "Mechanisms of migraine aura revealed by functional MRI in human visual cortex". Proc Natl Acad Sci U S A. 2001;98:4687-4692.

[83] M. de Tommaso, S. Stramaglia, D. Marinazzo, G. Trotta and M. Pellicoro, "Functional and effective connectivity in EEG alpha and beta bands during intermittent flash stimulation in migraine with and without aura", Cephalgia 2013, DOI: 10.1177/0333102413477741.

[84] G. Trotta, S. Stramaglia, M. Pellicoro, R. Bellotti, D. Marinazzo, M. de Tommaso, "Effective connectivity and cortical information flow under visual stimulation in Migraine with Aura", IWASI2013 Official Acts.
